# Supplementary material for: Sensitivity analysis enlightens effects of connectivity in a Neural Mass Model under Control-Target mode
Source: PLoS Comput Biol. 2026 Mar 23;22(3):e1014035. doi: 10.1371/journal.pcbi.1014035 (PMC13008111; doi:10.1371/journal.pcbi.1014035)
Supplement: S1 File — (PDF) [file pcbi.1014035.s001.pdf]

**Parameter**	**Value**	**Unit**
β^E	6.6	s^{-1}
α^E	72	$s^{-1}mM^{-1}$
T_{glu}	0.008	$mM\ s$
β^I	180	s^{-1}
α^I	530	$s^{-1}mM^{-1}$
T_{gaba}	0.003	$mM\ s$
a_E	310	nC^{-1}
b_E	125	Hz
d_E	0.16	s
a_I	615	nC^{-1}
b_I	177	Hz
d_I	0.087	s
W_E	1	—
W_I	0.7	—
I_0	0.382	nA
W_+	1.4	—
J_{nmda}	0.15	nA
J_{gaba_i}	[0.25;3.0]	nA
G	0.69	—
J_-	1	nA
