# Supplementary material for: Sensitivity analysis enlightens effects of connectivity in a Neural Mass Model under Control-Target mode
Source: PLoS Comput Biol. 2026 Mar 23;22(3):e1014035. doi: 10.1371/journal.pcbi.1014035 (PMC13008111; doi:10.1371/journal.pcbi.1014035)
Supplement: S2 File — (PDF) [file pcbi.1014035.s002.pdf]

Sensitivity analysis enlightens effects of connectivity in a Neural Mass Model under Control-Target mode: SI Formal developments for sensitivities and linear stability analysis

Vallet Anaïs[1], Blanco Stéphane[2], Chevallier Coline[2,3], Eustache Francis[1], Gautrais Jacques[2,3,*], Grandpeix Jean-Yves[4], Joly Jean-Louis[2], Segobin Shailendra[1], Gagnepain Pierre[1]

1 Normandie Univ, UNICAEN, PSL Research University, EPHE, INSERM, U1077, CHU de Caen, GIP Cyceron, Neuropsychologie et Imagerie de la Mémoire Humaine, 14000 Caen, France

2 LAPLACE, Université de Toulouse, CNRS, INPT, UPS, Toulouse, France

3 Centre de Recherches sur la Cognition Animale (CRCA), Centre de Biologie Intégrative (CBI), Université de Toulouse, CNRS, UPS, France

4 LMD/IPSL, Sorbonne Université, CNRS, École Polytechnique, ENS, Paris, France

* jacques.gautrais@cnrs.fr

This SI exposes the formal developments to build sensitivities in the coupled system as a hierarchy of nested sensitivities.

Contents

1 Sensitivity analysis for the isolated pools **4**

1.1 Isolated excitatory pool 4

1.2 Isolated inhibitory pool 5

1.3 Functional expressions of the sensitivities for the isolated excitatory and inhibitory pools 7

2 Sensitivity analysis for one isolated area **7**

2.1 Splitting x_{inter} from x_{intra} 8

2.2 Intermediate variable elimination 9

2.3 Expressing sensitivities w.r.t. forcings 10

2.4 Expressing Open Loop Sensitivities 12

2.5 Using Isolated Pool Sensitivities 13

2.6 Closed Loop Sensitivities as functions of Open Loop Sensitivities 14

2.7 Functional expressions of closed loop sensitivities 15

3 Two coupled area system **15**

3.1 Splitting x_{inter} from x_{intra} 16

3.2 Intermediate variables elimination 17

3.3 Expressing sensitivities w.r.t. forcings 18

3.3.1 Perturbation of one forcing B 18

3.3.2 Expressing Open Loop Sensitivities 20

3.4 Using Isolated Area Sensitivities 22

3.5 Sensitivities in a Target-Control system 26

4 Linear stability analysis **28**

4.1 For the isolated pools 28

4.1.1 Excitatory pool 29

4.1.2 Inhibitory pool 31

4.2 For one isolated area using Open Loop Sensitivities 33
4.2.1 Dynamic model 33
4.2.2 Splitting x_{inter} from x_{intra} 33
4.2.3 Intermediate variables elimination 34
4.2.4 Expressing the propagation matrix as a function of Open Loop Sensitivities . 35
4.3 For two coupled areas using Open Loop Sensitivities 39
4.3.1 Dynamic model 39
4.3.2 Splitting x_{inter} from x_{intra} 39
4.3.3 Intermediate variables elimination 40
4.3.4 Expressing the propagation matrix as a function of Open Loop Sensitivities . 42
4.3.5 Propagation matrix in a Control-Target system 51

1 Sensitivity analysis for the isolated pools

We consider in this section one isolated excitatory pool and one isolated inhibitory pool. Each pool contains an excitatory or inhibitory recurrent coupling and an excitatory forcing. Here, we are interested in the sensitivity of each excitatory and inhibitory pool activity to excitatory forcing.

1.1 Isolated excitatory pool

At the fixed point, the system reads:

$$\begin{cases} 0 = -\beta^E sn^* + \alpha^E T_{glu}(1 - sn^*)rn^* \equiv fn(sn^*, rn^*) \\ rn^* = \dfrac{a_E xn^* - b_E}{1 - e^{-d_E(a_E xn^* - b_E)}} \equiv hn(xn^*) \\ xn^* = W_+ J_{nmda} sn^* + z_E \equiv wn(sn^*, z_E) \end{cases} \tag{1}$$

With the perturbation of z_E, we get the new model:

$$\begin{cases} fn(sn^*, rn^*) = 0 \\ rn^* = hn(xn^*) \\ xn^* = wn(sn^*, z_E + \delta z_E) \end{cases} \tag{2}$$

We search the formal expression for the sensitivity $\frac{\delta sn}{\delta z_E}$.

The linearization for the perturbation around fixed point sn^* yields:

$$\begin{cases} \dfrac{\partial fn}{\partial sn}\delta sn + \dfrac{\partial fn}{\partial rn}\delta rn = 0 \\ \delta rn = \dfrac{\mathrm{d}hn}{\mathrm{d}xn}\delta xn \equiv hn' \delta xn \\ \delta xn = \dfrac{\partial wn}{\partial sn}\delta sn + \dfrac{\partial wn}{\partial z_E}\delta z_E \end{cases} \tag{3}$$

Plugging the last two equations into the first, we get:

$$\left(\frac{\partial fn}{\partial sn} + \frac{\partial fn}{\partial rn}\frac{\mathrm{d}hn}{\mathrm{d}xn}\frac{\partial wn}{\partial sn}\right)\delta sn = -\frac{\partial fn}{\partial rn}\frac{\mathrm{d}hn}{\mathrm{d}xn}\frac{\partial wn}{\partial z_E}\delta z_E \tag{4}$$

Rearranging to read how δsn depends upon δz_E:

$$\Big(1+\frac{\partial fn}{\partial sn}^{-1}\frac{\partial fn}{\partial rn}\frac{\mathrm{d}hn}{\mathrm{d}xn}\frac{\partial wn}{\partial sn}\Big)\delta sn=-\frac{\partial fn}{\partial sn}^{-1}\frac{\partial fn}{\partial rn}\frac{\mathrm{d}hn}{\mathrm{d}xn}\frac{\partial wn}{\partial z_E}\delta z_E \tag{5}$$

Then we get:

$$\frac{\delta sn}{\delta z_E}=\frac{-\frac{\partial fn}{\partial sn}^{-1}\frac{\partial fn}{\partial rn}\frac{\mathrm{d}hn}{\mathrm{d}xn}\frac{\partial wn}{\partial z_E}}{1+\frac{\partial fn}{\partial sn}^{-1}\frac{\partial fn}{\partial rn}\frac{\mathrm{d}hn}{\mathrm{d}xn}\frac{\partial wn}{\partial sn}} \tag{6}$$

$$=-\frac{1}{\frac{\partial fn}{\partial sn}\frac{\partial fn}{\partial rn}^{-1}\frac{\mathrm{d}hn}{\mathrm{d}xn}^{-1}\frac{\partial wn}{\partial z_E}^{-1}+\frac{\partial wn}{\partial sn}\frac{\partial wn}{\partial z_E}^{-1}} \tag{7}$$

Considering that

$$\frac{\partial fn}{\partial sn}=-\beta^E-\alpha^E T_{glu} rn^* \tag{8}$$

$$\frac{\partial fn}{\partial rn}=\alpha^E T_{glu}(1-sn^*) \tag{9}$$

$$\frac{\partial wn}{\partial sn}=W_+J_{nmda} \tag{10}$$

$$\frac{\partial wn}{\partial z_E}=1 \tag{11}$$

We can calculate:

$$\frac{\delta sn}{\delta z_E}=\frac{1}{\frac{\beta^E+\alpha^E T_{glu} rn^*}{\alpha^E T_{glu}(1-sn^*)\frac{\mathrm{d}hn}{\mathrm{d}xn}}-W_+J_{nmda}} \tag{12}$$

1.2 Isolated inhibitory pool

At the fixed point, the system reads:

$$\begin{cases} 0=-\beta^I sg^*+\alpha^I T_{gaba}(1-sg^*)rg^*\equiv fg(sg^*,rg^*) \\ rg^*=\dfrac{a_I xg^*-b_I}{1-e^{-d_I(a_I xg^*-b_I)}}\equiv hg(xg^*) \\ xg^*=-J_- sg^*+z_I\equiv wg(sg^*,z_I) \end{cases} \tag{13}$$

With the perturbation of z_I, we get the new model:

$$
\begin{cases}
fg(sg^*, rg^*) = 0 \\
rg^* = hg(xg^*) \\
xg^* = wg(sg^*, z_I + \delta z_I)
\end{cases}
\tag{14}
$$

We search the formal expression for the sensitivity $\frac{\delta sg}{\delta z_I}$.

The linearization for the perturbation around fixed point sg^* yields:

$$
\begin{cases}
\dfrac{\partial fg}{\partial sg}\delta sg + \dfrac{\partial fg}{\partial rg}\delta rg = 0 \\
\delta rg = \dfrac{\mathrm{d}hg}{\mathrm{d}xg}\delta xg \equiv hg'\delta xg \\
\delta xg = \dfrac{\partial wg}{\partial sg}\delta sg + \dfrac{\partial wg}{\partial z_I}\delta z_I
\end{cases}
\tag{15}
$$

Plugging the last two equations into the first, we get:

$$
(\frac{\partial fg}{\partial sg} + \frac{\partial fg}{\partial rg}\frac{\mathrm{d}hg}{\mathrm{d}xg}\frac{\partial wg}{\partial sg})\delta sg = -\frac{\partial fg}{\partial rg}\frac{\mathrm{d}hg}{\mathrm{d}xg}\frac{\partial wg}{\partial z_I}\delta z_I \tag{16}
$$

Rearranging to read how δsg depends upon δz_I:

$$
\big(1 + \frac{\partial fg}{\partial sg}^{-1}\frac{\partial fg}{\partial rg}\frac{\mathrm{d}hg}{\mathrm{d}xg}\frac{\partial wg}{\partial sg}\big)\delta sg = -\frac{\partial fg}{\partial sg}^{-1}\frac{\partial fg}{\partial rg}\frac{\mathrm{d}hg}{\mathrm{d}xg}\frac{\partial wg}{\partial z_I}\delta z_I \tag{17}
$$

Then we get:

$$
\frac{\delta sg}{\delta z_I} = \frac{-\frac{\partial fg}{\partial sg}^{-1}\frac{\partial fg}{\partial rg}\frac{\mathrm{d}hg}{\mathrm{d}xg}\frac{\partial wg}{\partial z_I}}{1 + \frac{\partial fg}{\partial sg}^{-1}\frac{\partial fg}{\partial rg}\frac{\mathrm{d}hg}{\mathrm{d}xg}\frac{\partial wg}{\partial sg}} \tag{18}
$$

$$
= -\frac{1}{\frac{\partial fg}{\partial sg}\frac{\partial fg}{\partial rg}^{-1}\frac{\mathrm{d}hg}{\mathrm{d}xg}^{-1}\frac{\partial wg}{\partial z_I}^{-1} + \frac{\partial wg}{\partial sg}\frac{\partial wg}{\partial z_I}^{-1}} \tag{19}
$$

Considering that

$$\frac{\partial fg}{\partial sg} = -\beta^I - \alpha^I T_{gaba} rg^* \tag{20}$$

$$\frac{\partial fg}{\partial rg} = \alpha^I T_{gaba}(1 - sg^*) \tag{21}$$

$$\frac{\partial wg}{\partial sg} = -J_- \tag{22}$$

$$\frac{\partial wg}{\partial z_I} = 1 \tag{23}$$

We can calculate:

$$\frac{\delta sg}{\delta z_I} = \frac{1}{\frac{\beta^I + \alpha^I T_{gaba} rg^*}{\alpha^I T_{gaba}(1-sg^*)\frac{\mathrm{d}hg}{\mathrm{d}xg}} + J_-} \tag{24}$$

1.3 Functional expressions of the sensitivities for the isolated excitatory and inhibitory pools

We define the function $\varphi n_{_E}$ and $\varphi g_{_I}$ to explicitly express the dependencies of the sensitivities for an isolated excitatory pool and an isolated inhibitory pool:

$$\begin{cases} \varphi n_{_E}(sn^*, z_E) \equiv \left(\dfrac{\beta^E + \alpha^E T_{glu} hn(wn(sn^*, z_E))}{\alpha^E T_{glu}(1 - sn^*) hn'(wn(sn^*, z_E))} - W_+ J_{nmda} \right)^{-1} \\ \varphi g_{_I}(sg^*, z_I) \equiv \left(\dfrac{\beta^I + \alpha^I T_{gaba} hg(wg(sg^*, z_I))}{\alpha^I T_{gaba}(1 - sg^*) hg'(wg(sg^*, z_I))} + J_- \right)^{-1} \end{cases} \tag{25}$$

where

$$h\square'(x) = \frac{(ae^{d(ax-b)}(e^{d(ax-b)} - adx + bd - 1))}{(e^{d(ad-b)} - 1)^2} \tag{26}$$

with a, b and d to be taken accordingly.

2 Sensitivity analysis for one isolated area

At the fixed point, the system with the two coupled pools reads:

$$\begin{cases} 0 = -\beta^E sn^* + \alpha^E T_{glu}(1 - sn^*)rn^* \equiv fn(sn^*, rn^*) \\ 0 = -\beta^I sg^* + \alpha^I T_{gaba}(1 - sg^*)rg^* \equiv fg(sg^*, rg^*) \end{cases} \tag{27}$$

with

$$\begin{cases} rn^* = \dfrac{a_E xn^* - b_E}{1 - e^{-d_E(a_E xn^* - b_E)}} \equiv hn(xn^*) \\ rg^* = \dfrac{a_I xg^* - b_I}{1 - e^{-d_I(a_I xg^* - b_I)}} \equiv hg(xg^*) \end{cases} \tag{28}$$

in which xn^* and xg^* represent the respective total input currents:

$$\begin{cases} xn^* = W_+ J_{nmda} sn^* - J_{gaba} sg^* + x_E \\ xg^* = J_{nmda} sn^* - J_- sg^* + x_I \end{cases} \tag{29}$$

where x_E and x_I represent basal forcings (effective external inputs), that we will respectively perturbate.

2.1 Splitting x_{inter} from x_{intra}

In order to express closed loop sensitivities of the pools as a function of their open loop sensitivities, we explicitly split the total input currents between an internal current, representing the effect of intra-pool recurrences:

$$\begin{cases} xn^*_{intra} = W_+ J_{nmda} sn^* \equiv wn_{intra}(sn^*) \\ xg^*_{intra} = -J_- sg^* \equiv wg_{intra}(sg^*) \end{cases} \tag{30}$$

and an external current, representing the total amount of forcing, due to the basic external forcing and the feedback current from the alternate pool:

$$\begin{cases} xn^*_{inter} = -J_{gaba} sg^* + x_E \equiv wn_{inter}(sg^*, x_E) \\ xg^*_{inter} = J_{nmda} sn^* + x_I \equiv wg_{inter}(sn^*, x_I) \end{cases} \tag{31}$$

so we have:

$$\begin{cases} xn^* = xn^*_{intra} + xn^*_{inter} \equiv wn(xn^*_{intra}, xn^*_{inter}) \\ xg^* = xg^*_{intra} + xg^*_{inter} \equiv wg(xg^*_{intra}, xg^*_{inter}) \end{cases} \tag{32}$$

2.2 Intermediate variable elimination

We define

$$\vec{s} = \begin{pmatrix} sn^* \\ sg^* \end{pmatrix}, \quad \vec{r} = \begin{pmatrix} rn^* \\ rg^* \end{pmatrix}, \quad \vec{x} = \begin{pmatrix} xn^* \\ xg^* \end{pmatrix}, \quad \overrightarrow{x_{intra}} = \begin{pmatrix} xn^*_{intra} \\ xg^*_{intra} \end{pmatrix}, \quad \overrightarrow{x_{inter}} = \begin{pmatrix} xn^*_{inter} \\ xg^*_{inter} \end{pmatrix} \tag{33}$$

so that we can summarize the fixed point as:

$$\begin{cases} \vec{f}(\vec{s}, \vec{r}) = \vec{0} \\ \vec{r} = \vec{h}(\vec{x}) \\ \vec{x} = \vec{w}(\overrightarrow{x_{intra}}, \overrightarrow{x_{inter}}) \\ \overrightarrow{x_{intra}} = \overrightarrow{w_{intra}}(\vec{s}) \\ \overrightarrow{x_{inter}} = \overrightarrow{w_{inter}}(\vec{s}, x_E, x_I) \end{cases} \tag{34}$$

Plugging intermediate variables $\vec{r}$, $\vec{x}$, $\overrightarrow{x_{intra}}$ into the first equation, Eq 34 can be rewritten as:

$$\begin{cases} \vec{F}(\vec{s}, \overrightarrow{x_{inter}}) = \vec{0} \\ \overrightarrow{x_{inter}} = \overrightarrow{w_{inter}}(\vec{s}, x_E, x_I) \end{cases} \tag{35}$$

where

$$\begin{cases} Fn(sn^*, xn^*_{inter}) \equiv -\beta^E sn^* + \alpha^E T_{glu}(1 - sn^*) \dfrac{a_E(W_+ J_{nmda} sn^* + xn^*_{inter}) - b_E}{1 - e^{-d_E[a_E(W_+ J_{nmda} sn^* + xn^*_{inter}) - b_E]}} \\ Fg(sg^*, xg^*_{inter}) = -\beta^I sg^* + \alpha^I T_{gaba}(1 - sg^*) \dfrac{a_I(-J_- sg^* + xg^*_{inter}) - b_I}{1 - e^{-d_I[a_I(-J_- sg^* + xg^*_{inter}) - b_I]}} \end{cases} \tag{36}$$

and where $\overrightarrow{x_{inter}}$, given by Eq 31, will be the support of information transfer between the two

pools (hence, denoted *transfer variables*).

2.3 Expressing sensitivities w.r.t. forcings

We start from Eq 35 where we consider the dependency to x_E.

With the perturbation, we get the new model:

$$\begin{cases} \vec{F}(\vec{s}, \overrightarrow{x_{inter}}) = \vec{0} \\ \overrightarrow{x_{inter}} = \overrightarrow{w_{inter}}(\vec{s}, x_E + \delta x_E, x_I) \end{cases} \tag{37}$$

We search the formal expression for:

$$\begin{pmatrix} \mathscr{A}_{sn,x_E} \\ \\ \mathscr{A}_{sg,x_E} \end{pmatrix} \equiv \begin{pmatrix} \frac{\delta sn}{\delta x_E} \\ \\ \frac{\delta sg}{\delta x_E} \end{pmatrix} = \frac{\overrightarrow{\delta s}}{\delta x_E} \tag{38}$$

The linearization for the perturbation around fixed points $\vec{s^*} = (sn^*, sg^*)$ and $\overrightarrow{x_{inter}} = (xn^*_{inter}, xg^*_{inter})$ yields:

$$\begin{cases} \overline{\overline{\frac{\partial F}{\partial s}}}\overrightarrow{\delta s} + \overline{\overline{\frac{\partial F}{\partial x_{inter}}}}\overrightarrow{\delta x_{inter}} = \vec{0} \\ \overrightarrow{\delta x_{inter}} = \overline{\overline{\frac{\partial w_{inter}}{\partial s}}}\overrightarrow{\delta s} + \overrightarrow{\frac{\partial w_{inter}}{\partial x_E}}\delta x_E \end{cases} \tag{39}$$

Plugging the last expression into the first, we get:

$$\overline{\overline{\frac{\partial F}{\partial s}}}\overrightarrow{\delta s} + \overline{\overline{\frac{\partial F}{\partial x_{inter}}\frac{\partial w_{inter}}{\partial s}}}\overrightarrow{\delta s} + \overline{\overline{\frac{\partial F}{\partial x_{inter}}}}\overrightarrow{\frac{\partial w_{inter}}{\partial x_E}}\delta x_E = \vec{0} \tag{40}$$

Rearranging to read how $\overrightarrow{\delta s}$ depends upon δx_E:

$$(\overline{\overline{\mathbb{I}}} + \overline{\overline{\frac{\partial F}{\partial s}}}^{-1}\overline{\overline{\frac{\partial F}{\partial x_{inter}}\frac{\partial w_{inter}}{\partial s}}})\overrightarrow{\delta s} = -\overline{\overline{\frac{\partial F}{\partial s}}}^{-1}\overline{\overline{\frac{\partial F}{\partial x_{inter}}}}\overrightarrow{\frac{\partial w_{inter}}{\partial x_E}}\delta x_E \tag{41}$$

Defining

$$\overline{\overline{S}} = -\overline{\overline{\frac{\partial F}{\partial s}}}^{-1} \overline{\overline{\frac{\partial F}{\partial x_{inter}}}} \tag{42}$$

we get:

$$\left(\overline{\overline{\mathbb{I}}} - \overline{\overline{S}}\,\overline{\overline{\frac{\partial w_{inter}}{\partial s}}}\right)\overrightarrow{\delta s} = \overline{\overline{S}}\,\overrightarrow{\frac{\partial w_{inter}}{\partial x_E}}\,\delta x_E \tag{43}$$

so that:

$$\begin{pmatrix} \mathscr{A}_{sn,x_E} \\ \\ \mathscr{A}_{sg,x_E} \end{pmatrix} = \overrightarrow{\frac{\delta s}{\delta x_E}} = \left(\overline{\overline{\mathbb{I}}} - \overline{\overline{S}}\,\overline{\overline{\frac{\partial w_{inter}}{\partial s}}}\right)^{-1}\overline{\overline{S}}\,\overrightarrow{\frac{\partial w_{inter}}{\partial x_E}} \tag{44}$$

Now turning to x_I, we can proceed the same way, expressing at fixed point the model with pertubation::

$$\begin{cases} \vec{F}(\vec{s}, \overrightarrow{x_{inter}}) = \vec{0} \\ \overrightarrow{x_{inter}} = \overrightarrow{w_{inter}}(\vec{s}, x_E, x_I + \delta x_I) \end{cases} \tag{45}$$

and we obtain:

$$\begin{pmatrix} \mathscr{A}_{sn,x_I} \\ \\ \mathscr{A}_{sg,x_I} \end{pmatrix} = \overrightarrow{\frac{\delta s}{\delta x_I}} = \left(\overline{\overline{\mathbb{I}}} - \overline{\overline{S}}\,\overline{\overline{\frac{\partial w_{inter}}{\partial s}}}\right)^{-1}\overline{\overline{S}}\,\overrightarrow{\frac{\partial w_{inter}}{\partial x_I}} \tag{46}$$

Using that:

$$\overrightarrow{\frac{\partial w_{inter}}{\partial x_E}} = \begin{bmatrix} 1 \\ 0 \end{bmatrix} \quad \text{and} \quad \overrightarrow{\frac{\partial w_{inter}}{\partial x_I}} = \begin{bmatrix} 0 \\ 1 \end{bmatrix} \tag{47}$$

we finally get a compact expression for the matrix of sensitivities to excitatory perturbations upon external forcings in either excitatory or inhibitory pool:

$$\begin{pmatrix} \mathscr{A}_{sn,x_E} & \mathscr{A}_{sn,x_I} \\ \mathscr{A}_{sg,x_E} & \mathscr{A}_{sg,x_I} \end{pmatrix} = \left(\overline{\overline{\mathbb{I}}} - \overline{\overline{S}}\,\overline{\overline{\frac{\partial w_{inter}}{\partial s}}}\right)^{-1}\overline{\overline{S}} \tag{48}$$

2.4 Expressing Open Loop Sensitivities

In the closed loop situation, from Eq 31, we have that:

$$\overline{\overline{\frac{\partial w_{inter}}{\partial s}}} = \begin{pmatrix} 0 & -J_{gaba} \\ J_{nmda} & 0 \end{pmatrix} \tag{49}$$

and in the open loop situation, we set: $\overline{\overline{\frac{\partial w_{inter}}{\partial s}}} = \overline{\overline{0}}$.

Under perturbation upon x_E, Eq 43 is

$$(\overline{\overline{\mathbb{I}}} - \underbrace{\overline{\overline{S}}\,\overline{\overline{\frac{\partial w_{inter}}{\partial s}}}}_{\overline{\overline{G}}})\overrightarrow{\delta s} = \overline{\overline{S}}\,\overrightarrow{\frac{\partial w_{inter}}{\partial x_E}}\delta x_E \tag{50}$$

where $\overline{\overline{G}}$ is the matrix of feedback gain due to the closed loop between the two pools.

In the open loop situation, $\overline{\overline{G}}$ is then nullified while the r.h.s. remains untouched.

In this case,

$$\overrightarrow{\delta s}^O = \overline{\overline{S}}\,\overrightarrow{\frac{\partial w_{inter}}{\partial x_E}}\delta x_E \tag{51}$$

represents the effect of perturbation upon fixed point values when the feedback loop has been opened.

The *legible* form can then be written as:

$$(\overline{\overline{\mathbb{I}}} - \overline{\overline{G}})\overrightarrow{\delta s} = \overrightarrow{\delta s}^O \tag{52}$$

We have:

$$\frac{\overrightarrow{\delta s}^O}{\delta x_E} = \overline{\overline{S}}\,\underbrace{\overrightarrow{\frac{\partial w_{inter}}{\partial x_E}}}_{\begin{bmatrix} 1 \\ 0 \end{bmatrix}} = \begin{pmatrix} \mathscr{A}^O_{sn,x_E} \\ 0 \end{pmatrix} \tag{53}$$

Obviously, in the open loop condition, the inhibitory pool is not affected by a perturbation upon x_E.

The same way, considering perturbation upon x_I, we get:

$$\frac{\overrightarrow{\delta s}^O}{\delta x_I} = \overline{\overline{S}} \underbrace{\frac{\overrightarrow{\partial w_{inter}}}{\partial x_I}}_{\begin{bmatrix} 0 \\ 1 \end{bmatrix}} = \begin{pmatrix} 0 \\ \mathscr{A}^O_{sg,x_I} \end{pmatrix} \tag{54}$$

In the open loop condition gain, the excitatory pool is not affected by a perturbation upon x_I. Hence $\overline{\overline{S}}$ reads:

$$\overline{\overline{S}} = \begin{pmatrix} \mathscr{A}^O_{sn,x_E} & 0 \\ 0 & \mathscr{A}^O_{sg,x_I} \end{pmatrix} \tag{55}$$

and represent the matrix of open loop sensitivities.

2.5 Using Isolated Pool Sensitivities

Now, lets consider z_E as:

$$z_E = -J_{gaba} sg^* + x_E = xn_{inter} = wn_{inter}(sg^*) \tag{56}$$

It represents the total amount of forcing at excitatory pool, due to the basic external forcing and the feedback current from the inhibitory pool.

Considering that perturbing here x_E is the same thing as perturbing z_E in the excitatory pool considered as an isolated pool, and that the same is true for the inhibitory pool, we then have

$$\begin{cases} \mathscr{A}^O_{sn,x_E} = \varphi n_{_E}(sn^*, -J_{gaba} sg^* + x_E) \\ \mathscr{A}^O_{sg,x_I} = \varphi g_{_I}(sg^*, J_{nmda} sn^* + x_I) \end{cases} \tag{57}$$

where $\varphi n_{_E}$ and $\varphi g_{_I}$ are given by Eq 25 and are to be evaluated at the fixed points sn^* and sg^* yielded by the closed loop system, and taking into account the total amount of external forcing.

2.6 Closed Loop Sensitivities as functions of Open Loop Sensitivities

We have

$$\frac{\overline{\overline{\partial w_{inter}}}}{\partial s} = \begin{bmatrix} \frac{\partial wn_{inter}}{\partial sn} & \frac{\partial wn_{inter}}{\partial sg} \\ \frac{\partial wg_{inter}}{\partial sn} & \frac{\partial wg_{inter}}{\partial sg} \end{bmatrix} = \begin{bmatrix} 0 & -J_{gaba} \\ J_{nmda} & 0 \end{bmatrix} \tag{58}$$

so, using Eq 55, we get:

$$(\bar{\bar{\mathbb{I}}} - \bar{\bar{S}}\frac{\overline{\overline{\partial w_{inter}}}}{\partial s}) = \bar{\bar{\mathbb{I}}} - \begin{bmatrix} \mathscr{A}^O_{sn,x_E} & 0 \\ 0 & \mathscr{A}^O_{sg,x_I} \end{bmatrix} \begin{bmatrix} 0 & -J_{gaba} \\ J_{nmda} & 0 \end{bmatrix} \tag{59}$$

$$= \bar{\bar{\mathbb{I}}} - \begin{bmatrix} 0 & -J_{gaba}\mathscr{A}^O_{sn,x_E} \\ J_{nmda}\mathscr{A}^O_{sg,x_I} & 0 \end{bmatrix} \tag{60}$$

$$= \begin{bmatrix} 1 & J_{gaba}\mathscr{A}^O_{sn,x_E} \\ -J_{nmda}\mathscr{A}^O_{sg,x_I} & 1 \end{bmatrix} \tag{61}$$

Inverting:

$$(\bar{\bar{\mathbb{I}}} - \bar{\bar{S}}\frac{\overline{\overline{\partial w_{inter}}}}{\partial s})^{-1} = \frac{1}{1 + J_{gaba}\mathscr{A}^O_{sn,x_E} J_{nmda}\mathscr{A}^O_{sg,x_I}} \begin{bmatrix} 1 & -J_{gaba}\mathscr{A}^O_{sn,x_E} \\ J_{nmda}\mathscr{A}^O_{sg,x_I} & 1 \end{bmatrix} \tag{62}$$

(63)

Finally,

$$\begin{pmatrix} \mathscr{A}_{sn,x_E} & \mathscr{A}_{sn,x_I} \\ \mathscr{A}_{sg,x_E} & \mathscr{A}_{sg,x_I} \end{pmatrix} = (\bar{\bar{\mathbb{I}}} - \bar{\bar{S}}\overline{\overline{\frac{\partial w_{inter}}{\partial s}}})^{-1}\bar{\bar{S}}$$
$$= \frac{1}{1 + J_{nmda}J_{gaba}\mathscr{A}^O_{sn,x_E}\mathscr{A}^O_{sg,x_I}} \times \begin{bmatrix} \mathscr{A}^O_{sn,x_E} & -J_{gaba}\mathscr{A}^O_{sn,x_E}\mathscr{A}^O_{sg,x_I} \\ J_{nmda}\mathscr{A}^O_{sn,x_E}\mathscr{A}^O_{sg,x_I} & \mathscr{A}^O_{sg,x_I} \end{bmatrix} \tag{64}$$

which expresses closed loop sensitivities (sensitivities for the pools when they are coupled) as functions of open loop sensitivities (sensitivities for the pools with only their recurrent coupling).

2.7 Functional expressions of closed loop sensitivities

We define the functions $\Phi n_{_E}$, $\Phi g_{_E}$, $\Phi n_{_I}$ and $\Phi g_{_I}$ to explicitly express the dependencies of the closed loop sensitivities for an isolated region:

$$\begin{cases} \mathscr{A}_{sn,x_E} = \Phi n_{_E}(\overrightarrow{s^*}, x_E, x_I) = \dfrac{\mathscr{A}^O_{sn,x_E}}{1 + J_{nmda}J_{gaba}\mathscr{A}^O_{sn,x_E}\mathscr{A}^O_{sg,x_I}} \\ \mathscr{A}_{sg,x_E} = \Phi g_{_E}(\overrightarrow{s^*}, x_E, x_I) = \dfrac{J_{nmda}\mathscr{A}^O_{sn,x_E}\mathscr{A}^O_{sg,x_I}}{1 + J_{nmda}J_{gaba}\mathscr{A}^O_{sn,x_E}\mathscr{A}^O_{sg,x_I}} \\ \mathscr{A}_{sn,x_I} = \Phi n_{_I}(\overrightarrow{s^*}, x_E, x_I) = \dfrac{-J_{gaba}\mathscr{A}^O_{sn,x_E}\mathscr{A}^O_{sg,x_I}}{1 + J_{nmda}J_{gaba}\mathscr{A}^O_{sn,x_E}\mathscr{A}^O_{sg,x_I}} \\ \mathscr{A}_{sg,x_I} = \Phi g_{_I}(\overrightarrow{s^*}, x_E, x_I) = \dfrac{\mathscr{A}^O_{sg,x_I}}{1 + J_{nmda}J_{gaba}\mathscr{A}^O_{sn,x_E}\mathscr{A}^O_{sg,x_I}} \end{cases} \tag{65}$$

where the open loop sensitivities are given in Eq 57.

3 Two coupled area system

We now turn to the coupling between two areas. We seek the expression of their closed loop sensitivities to perturbation as functions of their sensitivities when isolated, as it has been expressed

in the previous sections, hence, corresponding to their response to perturbation when the feedback loops are opened at the system scale.

3.1 Splitting x_{inter} from x_{intra}

At fixed point, the system can be expressed as:

$$\begin{cases} 0 = -\beta^E sn_i^* + \alpha^E T_{glu}(1 - sn_i^*) rn_i^* & \equiv fn_i(sn_i^*, rn_i^*) \\ 0 = -\beta^I sg_i^* + \alpha^I T_{gaba}(1 - sg_i^*) rg_i^* & \equiv fg_i(sn_i^*, rn_i^*) \end{cases} \tag{66}$$

with

$$\begin{cases} rn_i^* = \dfrac{a_E xn_i^* - b_E}{1 - e^{-d_E(a_E xn_i^* - b_E)}} & \equiv hn_i(xn_i^*) \\ rg_i^* = \dfrac{a_I xg_i^* - b_I}{1 - e^{-d_I(a_I xg_i^* - b_I)}} & \equiv hg_i(xg_i^*) \end{cases} \tag{67}$$

where $i \in \{1, 2\}$.

In Eq 67, xn_i^* and xg_i^* represent the respective total input current to area i. In order to express closed loop sensitivities of the areas as a function of their open loop sensitivities, we explicitly split this total input current between an internal current within an area, due to intra-pool recurrence, and coupling between pools:

$$\begin{cases} xn_{intra,i}^* = W_+ J_{nmda} sn_i^* - J_{gaba_i} sg_i^* & \equiv wn_{intra,i}(sn_i^*, sg_i^*) \\ xg_{intra,i}^* = J_{nmda} sn_i^* - J_- sg_i^* & \equiv wg_{intra,i}(sn_i^*, sg_i^*) \end{cases} \tag{68}$$

and external current due to the external inputs and the coupling between the two areas:

$$\begin{cases} xn_{inter,i}^* = k_{E_{ij}} \kappa_{ij} sn_j^* + B_{E_i} & \equiv wn_{inter,i}(sn_j^*) \quad j \neq i \\ xg_{inter,i}^* = (1 - k_{E_{ij}}) \kappa_{ij} sn_j^* + B_{I_i} & \equiv wg_{inter,i}(sn_j^*) \quad j \neq i \end{cases} \tag{69}$$

so that total input currents reads:

$$\begin{cases} xn_i^* = xn_{intra,i}^* + xn_{inter,i}^* = wn_i(xn_{intra,i}^*, xn_{inter,i}^*) \\ xg_i^* = xg_{intra,i}^* + xg_{inter,i}^* = wg_i(xg_{intra,i}^*, xg_{inter,i}^*) \end{cases} \tag{70}$$

3.2 Intermediate variables elimination

We define

$$\vec{s} = \begin{pmatrix} sn_1^* \\ sn_2^* \\ sg_1^* \\ sg_2^* \end{pmatrix}, \quad \vec{r} = \begin{pmatrix} rn_1^* \\ rn_2^* \\ rg_1^* \\ rg_2^* \end{pmatrix}, \quad \vec{x} = \begin{pmatrix} xn_1^* \\ xn_2^* \\ xg_1^* \\ xg_2^* \end{pmatrix}, \quad \overrightarrow{x_{intra}} = \begin{pmatrix} xn_{intra,1}^* \\ xn_{intra,2}^* \\ xg_{intra,1}^* \\ xg_{intra,2}^* \end{pmatrix}, \quad \overrightarrow{x_{inter}} = \begin{pmatrix} xn_{inter,1}^* \\ xn_{inter,2}^* \\ xg_{inter,1}^* \\ xg_{inter,2}^* \end{pmatrix} \tag{71}$$

and

$$\overrightarrow{B_E} = \begin{pmatrix} B_{E_1} \\ B_{E_2} \end{pmatrix}, \quad \overrightarrow{B_I} = \begin{pmatrix} B_{I_1} \\ B_{I_2} \end{pmatrix} \tag{72}$$

so that we can summarize the fixed point as:

$$\begin{cases} \vec{f}(\vec{s}, \vec{r}) = \vec{0} \\ \vec{r} = \vec{h}(\vec{x}) \\ \vec{x} = \vec{w}(\overrightarrow{x_{intra}}, \overrightarrow{x_{inter}}) \\ \overrightarrow{x_{intra}} = \overrightarrow{w_{intra}}(\vec{s}) \\ \overrightarrow{x_{inter}} = \overrightarrow{w_{inter}}(\vec{s}, \overrightarrow{B_E}, \overrightarrow{B_I}) \end{cases} \tag{73}$$

Plugging intermediate variables $\vec{r}$, $\vec{x}$, $\overrightarrow{x_{intra}}$ into the first equation, Eq 73 can be rewritten as:

$$\begin{cases} \overrightarrow{F}(\overrightarrow{s}, \overrightarrow{x_{inter}}) = \overrightarrow{0} \\ \overrightarrow{x_{inter}} = \overrightarrow{w_{inter}}(\overrightarrow{s}, \overrightarrow{B_E}, \overrightarrow{B_I}) \end{cases} \tag{74}$$

where

$$\begin{cases} Fn_i(sn_i^*, sg_i^*, xn_{inter,i}^*) = -\beta^E sn_i^* \\ \qquad + \alpha^E T_{glu}(1 - sn_i^*) \dfrac{a_E(W_+ J_{nmda} sn_i^* - J_{gaba_i} sg_i^* + xn_{inter,i}^*) - b_E}{1 - e^{-d_E[a_E(W_+ J_{nmda} sn_i^* - J_{gaba_i} sg_i^* + xn_{inter,i}^*) - b_E]}} \\ Fg_i(sn_i^*, sg_i^*, xg_{inter,i}^*) = -\beta^I sg_i^* \\ \qquad + \alpha^I T_{gaba}(1 - sg_i^*) \dfrac{a_I(J_{nmda} sn_i^* - J_- sg_i^* + xg_{inter,i}^*) - b_I}{1 - e^{-d_I[a_I(J_{nmda} sn_i^* - J_- sg_i^* + xg_{inter,i}^*) - b_I]}} \end{cases} \tag{75}$$

and where $\overrightarrow{x_{inter}}$, given by Eq 69, will be the transfer variables.

3.3 Expressing sensitivities w.r.t. forcings

3.3.1 Perturbation of one forcing B

Here, we build the general expression of the sensitivity, for any given forcing B among B_{E_1}, B_{E_2}, B_{I_1} or B_{I_2}.

The perturbed form of system 74 reads:

$$\begin{cases} \overrightarrow{F}(\overrightarrow{s}, \overrightarrow{x_{inter}}) = \overrightarrow{0} \\ \overrightarrow{x_{inter}} = \overrightarrow{w_{inter}}(\overrightarrow{s}, B + \delta B) \end{cases} \tag{76}$$

By linearization at fixed point, we get:

$$\begin{cases} \overline{\overline{\frac{\partial F}{\partial s}}}\overrightarrow{\delta s_B} + \overline{\overline{\frac{\partial F}{\partial x_{inter}}}}\overrightarrow{\delta x_{inter B}} = \overrightarrow{0} \\ \overrightarrow{\delta x_{inter B}} = \overline{\overline{\frac{\partial w_{inter}}{\partial s}}}\overrightarrow{\delta s_B} + \overrightarrow{\frac{\partial w_{inter}}{\partial B}}\delta B \end{cases} \tag{77}$$

Plugging the second equation into the first, we have:

$$(\overline{\overline{\frac{\partial F}{\partial s}}} + \overline{\overline{\frac{\partial F}{\partial x_{inter}}\frac{\partial w_{inter}}{\partial s}}})\overrightarrow{\delta s_B} = -\overline{\overline{\frac{\partial F}{\partial x_{inter}}}}\overrightarrow{\frac{\partial w_{inter}}{\partial B}}\delta B \tag{78}$$

Denoting

$$\overline{\overline{S}} = -\overline{\overline{\frac{\partial F}{\partial s}}}^{-1}\overline{\overline{\frac{\partial F}{\partial x_{inter}}}} \tag{79}$$

expression 78 can be written under the *legible* form as:

$$(\overline{\overline{\mathbb{I}}} - \overline{\overline{S}}\,\overline{\overline{\frac{\partial w_{inter}}{\partial s}}})\overrightarrow{\delta s_B} = \overline{\overline{S}}\overrightarrow{\frac{\partial w_{inter}}{\partial B}}\delta B \tag{80}$$

In this expression $\overline{\overline{\frac{\partial w_{inter}}{\partial s}}}$ represents the coupling between the two areas, and is:

$$\overline{\overline{\frac{\partial w_{inter}}{\partial s}}} = \begin{pmatrix} 0 & \frac{\partial wn_{inter,1}}{\partial sn_2} & 0 & 0 \\ \frac{\partial wn_{inter,2}}{\partial sn_1} & 0 & 0 & 0 \\ 0 & \frac{\partial wg_{inter,1}}{\partial sn_2} & 0 & 0 \\ \frac{\partial wg_{inter,2}}{\partial sn_1} & 0 & 0 & 0 \end{pmatrix} = \begin{pmatrix} 0 & k_{E_{12}}\kappa_{12} & 0 & 0 \\ k_{E_{21}}\kappa_{21} & 0 & 0 & 0 \\ 0 & (1-k_{E_{12}})\kappa_{12} & 0 & 0 \\ (1-k_{E_{21}})\kappa_{21} & 0 & 0 & 0 \end{pmatrix} \tag{81}$$

If we set $\overline{\overline{\frac{\partial w_{inter}}{\partial s}}} = \overline{\overline{0}}$ while the r.h.s. remains untouched, we then obtain the effect of perturbation in the open loop case:

$$\overrightarrow{\delta s_B^O} = \overline{\overline{S}}\overrightarrow{\frac{\partial w_{inter}}{\partial B}}\delta B \tag{82}$$

The *legible* expression then reads:

$$\boxed{(\bar{\bar{\mathbb{I}}} - \bar{\bar{G}})\overrightarrow{\delta s_B} = \overrightarrow{\delta s_B^O}} \tag{83}$$

where

$$\bar{\bar{G}} = -\overline{\overline{\frac{\partial F}{\partial s}}}^{-1} \overline{\overline{\frac{\partial F}{\partial x_{inter}} \frac{\partial w_{inter}}{\partial s}}} = \bar{\bar{S}} \overline{\overline{\frac{\partial w_{inter}}{\partial s}}} \tag{84}$$

is the feedback gain matrix.

3.3.2 Expressing Open Loop Sensitivities

Perturbations are operated upon transfer variables, given by $\overrightarrow{w_{inter}}$.

Let $\overrightarrow{x_{inter}}^O$ denote the transfer variables in open loop condition when $\overline{\overline{\frac{\partial w_{inter}}{\partial s}}} = \bar{\bar{0}}$ (i.e. $\kappa_{12} = \kappa_{21} = 0$):

$$\overrightarrow{x_{inter}}^O = \begin{pmatrix} B_{E_1} \\ B_{E_2} \\ B_{I_1} \\ B_{I_2} \end{pmatrix} \tag{85}$$

Since forcing parameters are independent, we have: $\overline{\overline{\frac{\partial w_{inter}}{\partial x^O_{inter}}}} = \bar{\bar{\mathbb{I}}}$.

Hence considering each perturbation one by one, the operator $\overrightarrow{\frac{\partial w_{inter}}{\partial B}}$ acts as a selector of a column of $\bar{\bar{S}}$, so that we can express the sensitivities in closed loop condition as function of sensitivities in open loop condition.

For instance, perturbing B_{E_1}, we get:

$$\overrightarrow{\delta s}^{O}_{B_{E_1}} = \overline{\overline{S}}\,\overrightarrow{\frac{\partial w_{inter}}{\partial B_{E_1}}}\delta B_{E_1} \Longleftrightarrow \frac{\overrightarrow{\delta s}^{O}_{B_{E_1}}}{\delta B_{E_1}} = \overline{\overline{S}}\,\underbrace{\overrightarrow{\frac{\partial w_{inter}}{\partial B_{E_1}}}}_{\begin{bmatrix}1\\0\\0\\0\end{bmatrix}} \tag{86}$$

hence the open loop sensitivities of both areas to a perturbation upon the excitatory pool of the first area yields the first column of $\overline{\overline{S}}$.

Obviously, in the open loop condition, the second area is not perturbed at all.

Furthermore, considering that perturbing here B_{E_1} is the same thing as perturbing x_{E_1} in the first area considered as an isolated area, we have, by definition given in Eq 38, that:

$$\overrightarrow{\delta s}^{O}_{B_{E_1}} = \begin{pmatrix} \delta sn_1 \\ \delta sn_2 \\ \delta sg_1 \\ \delta sg_2 \end{pmatrix}^{O}_{B_{E_1}} = \begin{pmatrix} \mathscr{A}_{sn_1,x_{E_1}}\delta B_{E_1} \\ 0 \\ \mathscr{A}_{sg_1,x_{E_1}}\delta B_{E_1} \\ 0 \end{pmatrix} \tag{87}$$

hence the first column of $\overline{\overline{S}}$ is given by:

$$\frac{\overrightarrow{\delta s}^{O}_{B_{E_1}}}{\delta B_{E_1}} = \begin{pmatrix} \mathscr{A}_{sn_1,x_{E_1}} \\ 0 \\ \mathscr{A}_{sg_1,x_{E_1}} \\ 0 \end{pmatrix} \tag{88}$$

Following the same lines of reasoning for the three other perturbations, we finally obtain:

$$\overline{\overline{S}} = \begin{pmatrix} \overline{\overline{\mathscr{A}_{sn,x_E}}} & \overline{\overline{\mathscr{A}_{sn,x_I}}} \\ \overline{\overline{\mathscr{A}_{sg,x_E}}} & \overline{\overline{\mathscr{A}_{sg,x_I}}} \end{pmatrix} \tag{89}$$

with

$$\overline{\overline{\mathscr{A}_{sn,x_E}}} = \begin{pmatrix} \mathscr{A}_{sn_1,x_{E_1}} & 0 \\ 0 & \mathscr{A}_{sn_2,x_{E_2}} \end{pmatrix}, \qquad \overline{\overline{\mathscr{A}_{sn,x_I}}} = \begin{pmatrix} \mathscr{A}_{sn_1,x_{I_1}} & 0 \\ 0 & \mathscr{A}_{sn_2,x_{I_2}} \end{pmatrix} \tag{90}$$

$$\overline{\overline{\mathscr{A}_{sg,x_E}}} = \begin{pmatrix} \mathscr{A}_{sg_1,x_{E_1}} & 0 \\ 0 & \mathscr{A}_{sg_2,x_{E_2}} \end{pmatrix}, \qquad \overline{\overline{\mathscr{A}_{sg,x_I}}} = \begin{pmatrix} \mathscr{A}_{sg_1,x_{I_1}} & 0 \\ 0 & \mathscr{A}_{sg_2,x_{I_2}} \end{pmatrix} \tag{91}$$

3.4 Using Isolated Area Sensitivities

In the same spirit as in Sec 2.5, these open loop sensitivities of areas can be expressed by the analytical expression of their sensitivities when considered isolated, yet to be evaluated respectively at fixed points $\overrightarrow{s_1}^*$ and $\overrightarrow{s_2}^*$ yielded by the closed loop system and taking into account the total amount of external forcing, so we write:

$$\begin{cases} \mathscr{A}_{sn_i,x_{E_i}} = \Phi n_{_E}(\overrightarrow{s^*} = \overrightarrow{s_i^*}, x_E = xn^*_{inter,i}, x_I = xg^*_{inter,i}) \\ \mathscr{A}_{sg_i,x_{E_i}} = \Phi g_{_E}(\overrightarrow{s^*} = \overrightarrow{s_i^*}, x_E = xn^*_{inter,i}, x_I = xg^*_{inter,i}) \\ \mathscr{A}_{sn_i,x_{I_i}} = \Phi n_{_I}(\overrightarrow{s^*} = \overrightarrow{s_i^*}, x_E = xn^*_{inter,i}, x_I = xg^*_{inter,i}) \\ \mathscr{A}_{sg_i,x_{I_i}} = \Phi g_{_I}(\overrightarrow{s^*} = \overrightarrow{s_i^*}, x_E = xn^*_{inter,i}, x_I = xg^*_{inter,i}) \end{cases} \tag{92}$$

where $xn^*_{inter,i}$ and $x_I = xg^*_{inter,i}$ are defined in Eq 69. Analytical expression for the functions $\Phi n_{_E}$, $\Phi g_{_E}$, $\Phi n_{_I}$ and $\Phi g_{_I}$ are explicitly given in section 2.7.

Closed Loop Sensitivities as functions of Open Loop Sensitivities

From definition 84 for $\overline{\overline{G}}$, we have:

$$\overline{\overline{G}} = \begin{pmatrix} \mathscr{A}_{sn_1,x_{E_1}} & 0 & \mathscr{A}_{sn_1,x_{I_1}} & 0 \\ 0 & \mathscr{A}_{sn_2,x_{E_2}} & 0 & \mathscr{A}_{sn_2,x_{I_2}} \\ \mathscr{A}_{sg_1,x_{E_1}} & 0 & \mathscr{A}_{sg_1,x_{I_1}} & 0 \\ 0 & \mathscr{A}_{sg_2,x_{E_2}} & 0 & \mathscr{A}_{sg_2,x_{I_2}} \end{pmatrix} \begin{bmatrix} 0 & k_{E_{12}}\kappa_{12} & 0 & 0 \\ k_{E_{21}}\kappa_{21} & 0 & 0 & 0 \\ 0 & (1-k_{E_{12}})\kappa_{12} & 0 & 0 \\ (1-k_{E_{21}})\kappa_{21} & 0 & 0 & 0 \end{bmatrix} \tag{93}$$

that we will write as:

$$\overline{\overline{G}} = \begin{bmatrix} 0 & G_{12} & 0 & 0 \\ G_{21} & 0 & 0 & 0 \\ 0 & G_{32} & 0 & 0 \\ G_{41} & 0 & 0 & 0 \end{bmatrix} \tag{94}$$

with

$$\begin{cases} G_{12} = \mathscr{A}_{sn_1,x_{E_1}} k_{E_{12}}\kappa_{12} + \mathscr{A}_{sn_1,x_{I_1}}(1-k_{E_{12}})\kappa_{12} \\ G_{21} = \mathscr{A}_{sn_2,x_{E_2}} k_{E_{21}}\kappa_{21} + \mathscr{A}_{sn_2,x_{I_2}}(1-k_{E_{21}})\kappa_{21} \\ G_{32} = \mathscr{A}_{sg_1,x_{E_1}} k_{E_{12}}\kappa_{12} + \mathscr{A}_{sg_1,x_{I_1}}(1-k_{E_{12}})\kappa_{12} \\ G_{41} = \mathscr{A}_{sg_2,x_{E_2}} k_{E_{21}}\kappa_{21} + \mathscr{A}_{sg_2,x_{I_2}}(1-k_{E_{21}})\kappa_{21} \end{cases} \tag{95}$$

For the l.h.s. term in expression 83, we then have:

$$\bar{\bar{\mathbb{I}}} - \overline{\overline{G}} = \left(\begin{array}{cc|cc} 1 & -G_{12} & 0 & 0 \\ -G_{21} & 1 & 0 & 0 \\ \hline 0 & -G_{32} & 1 & 0 \\ -G_{41} & 0 & 0 & 1 \end{array}\right) \tag{96}$$

that we will write as:

$$\bar{\bar{\mathbb{I}}} - \overline{\overline{G}} = \left(\begin{array}{c|c} \overline{\overline{B_1}} & \overline{\overline{B_2}} \\ \hline \overline{\overline{B_3}} & \overline{\overline{B_4}} \end{array}\right) \tag{97}$$

Considering the property that

$$\text{If } \overline{\overline{M}} = \begin{pmatrix} \overline{\overline{A}} & \overline{\overline{B}} \\ \overline{\overline{C}} & \overline{\overline{D}} \end{pmatrix} \text{ with } \overline{\overline{D}} \text{ invertible}$$

$$\text{then } \overline{\overline{M}}^{-1} = \begin{pmatrix} \overline{\overline{R}} & \overline{\overline{S}} \\ \overline{\overline{T}} & \overline{\overline{U}} \end{pmatrix} \text{ with } \begin{cases} \overline{\overline{R}} = \left(\overline{\overline{A}} - \overline{\overline{BD}}^{-1}\overline{\overline{C}}\right)^{-1} \\ \overline{\overline{S}} = -\overline{\overline{RBD}}^{-1} \\ \overline{\overline{T}} = -\overline{\overline{D}}^{-1}\overline{\overline{CR}} \\ \overline{\overline{U}} = \overline{\overline{D}}^{-1}\left(\bar{\bar{\mathbb{I}}} - \overline{\overline{CS}}\right) \end{cases}$$

we obtain

$$
\left(\overline{\overline{\mathbb{I}}} - \overline{\overline{G}}\right)^{-1} = \left(\begin{array}{c|c} \overline{\overline{C_1}} & \overline{\overline{C_2}} \\ \hline \overline{\overline{C_3}} & \overline{\overline{C_4}} \end{array}\right) \text{ where } \begin{cases} \overline{\overline{C_1}} = \left(\overline{\overline{B_1}} - \overline{\overline{B_2}}(\overline{\overline{B_4}}^{-1})\overline{\overline{B_3}}\right)^{-1} = \overline{\overline{B_1}}^{-1} \\ \overline{\overline{C_2}} = -\overline{\overline{C_1 B_2}}(\overline{\overline{B_4}}^{-1}) = \overline{\overline{0}} \\ \overline{\overline{C_3}} = -\overline{\overline{B_4}}^{-1}\overline{\overline{B_3 C_1}} = -\overline{\overline{B_3 C_1}} = -\overline{\overline{B_3 B_1}}^{-1} \\ \overline{\overline{C_4}} = \overline{\overline{B_4}}^{-1}\left(\overline{\overline{\mathbb{I}}} - \overline{\overline{B_3 C_2}}\right) = \overline{\overline{\mathbb{I}}} \end{cases} \tag{98}
$$

$$
= \left(\begin{array}{c|c} \overline{\overline{B_1}}^{-1} & \overline{\overline{0}} \\ \hline -\overline{\overline{B_3 B_1}}^{-1} & \overline{\overline{\mathbb{I}}} \end{array}\right) \tag{99}
$$

To recover sensitivities for the closed loop condition, we then consider:

$$
(\overline{\overline{\mathbb{I}}} - \overline{\overline{G}})^{-1}\overline{\overline{S}} = \left(\begin{array}{c|c} \overline{\overline{B_1}}^{-1} & \overline{\overline{0}} \\ \hline -\overline{\overline{B_3 B_1}}^{-1} & \overline{\overline{\mathbb{I}}} \end{array}\right) \left(\begin{array}{c|c} \overline{\overline{\mathscr{A}_{sn,x_E}}} & \overline{\overline{\mathscr{A}_{sn,x_I}}} \\ \hline \overline{\overline{\mathscr{A}_{sg,x_E}}} & \overline{\overline{\mathscr{A}_{sg,x_I}}} \end{array}\right) \tag{100}
$$

$$
= \left(\begin{array}{c|c} \overline{\overline{B_1}}^{-1}\overline{\overline{\mathscr{A}_{sn,x_E}}} & \overline{\overline{B_1}}^{-1}\overline{\overline{\mathscr{A}_{sn,x_I}}} \\ \hline -\overline{\overline{B_3 B_1}}^{-1}\overline{\overline{\mathscr{A}_{sn,x_E}}} + \overline{\overline{\mathscr{A}_{sg,x_E}}} & -\overline{\overline{B_3 B_1}}^{-1}\overline{\overline{\mathscr{A}_{sn,x_I}}} + \overline{\overline{\mathscr{A}_{sg,x_I}}} \end{array}\right) \tag{101}
$$

so that

$$
\boxed{\frac{\overrightarrow{\delta s_B}}{\delta B} = \left(\overline{\overline{\mathbb{I}}} - \overline{\overline{G}}\right)^{-1}\overline{\overline{S}}\,\frac{\overrightarrow{\partial w_{inter}}}{\partial B}} \tag{102}
$$

expresses, in full generality for the two-area system, the matrix of sensitivities to a perturbation upon either forcing B. They are expressed as functions of the sensitivities in single-area system, which are in turn expressed as functions of the sensitivities in the single-pool system.

3.5 Sensitivities in a Target-Control system

We now focus on the question of how sensitivities would drive the response of the excitatory pool of one area to the activation of the excitatory pool of the other one, depending on the connectivity between the two areas. Hence, we attribute a role to each area: the area 1 which excitatory pool is positively perturbed will be called "Control" area (denoted by C), and the area 2 will be called "Target area" (denoted by T).

From now on, the observable will then be denoted as:

$$\vec{s} = \begin{pmatrix} sn_C \\ sn_T \\ sg_C \\ sg_T \end{pmatrix} \tag{103}$$

and the transfer variables as:

$$\vec{x} = \begin{pmatrix} xn_{intra,C} & = & B_{E_C} & + & k_{E_{CT}}\kappa_{CT} sn_T \\ xn_{intra,T} & = & B_{E_T} & + & k_{E_{TC}}\kappa_{TC} sn_C \\ xg_{intra,C} & = & B_{I_C} & + & (1 - k_{E_{CT}})\kappa_{CT} sn_T \\ xg_{intra,T} & = & B_{I_T} & + & (1 - k_{E_{TC}})\kappa_{TC} sn_C \end{pmatrix} \tag{104}$$

and we focus on:

$$\begin{pmatrix} \delta sn_T \\ \delta sn_C \end{pmatrix} \tag{105}$$

in response to δB_{E_C}.

To extract the situation of interest, from the general result above, we then pick the case:

$$\overrightarrow{\delta s}_{B_{E_C}} = (\bar{\bar{\mathbb{I}}} - \overline{\overline{G}})^{-1}\overline{\overline{S}}\,\overrightarrow{\frac{\partial w_{inter}}{\partial B_{E_C}}}\,\delta B_{E_C} \tag{106}$$

$$= \left(\begin{array}{c|c} \overline{\overline{B_1}}^{-1}\overline{\overline{\mathscr{A}_{sn,x_E}}} & \overline{\overline{B_1}}^{-1}\overline{\overline{\mathscr{A}_{sn,x_I}}} \\ \hline -\overline{\overline{B_3 B_1}}^{-1}\overline{\overline{\mathscr{A}_{sn,x_E}}} + \overline{\overline{\mathscr{A}_{sg,x_E}}} & -\overline{\overline{B_3 B_1}}^{-1}\overline{\overline{\mathscr{A}_{sn,x_I}}} + \overline{\overline{\mathscr{A}_{sg,x_I}}} \end{array}\right) \left(\begin{array}{c} 1 \\ 0 \\ \hline 0 \\ 0 \end{array}\right) \delta B_{E_C} \tag{107}$$

$$= \left(\begin{array}{c} \overline{\overline{B_1}}^{-1}\overline{\overline{\mathscr{A}_{sn,x_E}}} \begin{pmatrix} 1 \\ 0 \end{pmatrix} \\ \hline \left(-\overline{\overline{B_3 B_1}}^{-1}\overline{\overline{\mathscr{A}_{sn,x_E}}} + \overline{\overline{\mathscr{A}_{sg,x_E}}}\right) \begin{pmatrix} 1 \\ 0 \end{pmatrix} \end{array}\right) \delta B_{E_C} \tag{108}$$

$$\equiv \left(\begin{array}{c} \delta sn_C \\ \delta sn_T \\ \hline \delta sg_C \\ \delta sg_T \end{array}\right) \tag{109}$$

where the perturbations of interest are in the upper part, and we have:

$$\begin{pmatrix} \delta sn_C \\ \delta sn_T \end{pmatrix} = \overline{\overline{B_1}}^{-1}\overline{\overline{\mathscr{A}_{sn,x_E}}} \begin{pmatrix} 1 \\ 0 \end{pmatrix} \delta B_{E_C} \tag{110}$$

From definition 97, we get

$$\overline{\overline{B_1}}^{-1} = \begin{pmatrix} 1 & -G_{12} \\ -G_{21} & 1 \end{pmatrix}^{-1} = \frac{1}{1 - G_{12}G_{21}} \begin{pmatrix} 1 & G_{12} \\ G_{21} & 1 \end{pmatrix} \tag{111}$$

hence

$$\begin{pmatrix} \delta sn_C \\ \delta sn_T \end{pmatrix} = \frac{1}{1 - G_{12}G_{21}} \begin{pmatrix} 1 & G_{12} \\ G_{21} & 1 \end{pmatrix} \begin{pmatrix} \mathscr{A}_{sn_C,x_{E_C}} & 0 \\ 0 & \mathscr{A}_{sn_T,x_{E_T}} \end{pmatrix} \begin{pmatrix} 1 \\ 0 \end{pmatrix} \delta B_{E_C} \tag{112}$$

$$= \frac{1}{1 - G_{12}G_{21}} \begin{pmatrix} \mathscr{A}_{sn_C,x_{E_C}} & G_{12}\mathscr{A}_{sn_T,x_{E_T}} \\ G_{21}\mathscr{A}_{sn_C,x_{E_C}} & \mathscr{A}_{sn_T,x_{E_T}} \end{pmatrix} \begin{pmatrix} 1 \\ 0 \end{pmatrix} \delta B_{E_C} \tag{113}$$

$$= \frac{1}{1 - G_{12}G_{21}} \begin{pmatrix} \mathscr{A}_{sn_C,x_{E_C}} \\ G_{21}\mathscr{A}_{sn_C,x_{E_C}} \end{pmatrix} \delta B_{E_C} \tag{114}$$

with

$$\begin{cases} G_{12} = \mathscr{A}_{sn_C,x_{E_C}} k_{E_{CT}} \kappa_{CT} + \mathscr{A}_{sn_C,x_{I_C}} (1 - k_{E_{CT}}) \kappa_{CT} \\ G_{21} = \mathscr{A}_{sn_T,x_{E_T}} k_{E_{TC}} \kappa_{TC} + \mathscr{A}_{sn_T,x_{I_T}} (1 - k_{E_{TC}}) \kappa_{TC} \end{cases} \tag{115}$$

For the sake of clarity, G_{12} and G_{21} are denoted G_{CT} and G_{TC} in the main text.

4 Linear stability analysis

We present the formal developments for a linear stability analysis in the cases of isolated pools, one region and two regions. For one and two regions, we construct the propagation matrix using open loop sensitivities expression (presented in 2 and 3), thus leveraging our nested levels methodology. For one region, we show in **??** that the propagation matrix expressed as a function of open loop sensitivities is exactly equal to the one built with traditional Jacobian-based developments.

Stability of fixed points has then been checked numerically. The real part of eigenvalues are all negative, so fixed points presented in the article are stable in all cases.

4.1 For the isolated pools

4.1.1 Excitatory pool

The dynamic system reads:

$$\begin{cases} \dfrac{dsn(t)}{dt} = -\beta^E sn(t) + \alpha^E T_{glu}(1 - sn(t))rn(t) \equiv fn(sn(t), rn(t)) \\ rn(t) = \dfrac{a_E xn(t) - b_E}{1 - e^{-d_E(a_E xn(t) - b_E)}} \equiv hn(xn(t)) \\ xn(t) = W_+ J_{nmda} sn(t) + z_E \equiv wn(sn(t), z_E) \end{cases} \tag{116}$$

We stand near the fixed point: $sn = sn^* + \delta sn$.

With the perturbation of δsn, we get the new model:

$$\begin{cases} \dfrac{d}{dt}(sn^* + \delta sn) = fn(sn^* + \delta sn, rn(t)) \\ rn(t) = hn(xn(t)) \\ xn(t) = wn(sn(t), z_E) \end{cases} \tag{117}$$

By linearization near the fixed point, we get:

$$\begin{cases} \dfrac{d}{dt}(\delta sn) = \dfrac{\partial fn}{\partial sn}\delta sn + \dfrac{\partial fn}{\partial rn}\delta rn \\ \delta rn = \dfrac{\partial hn}{\partial xn}\delta xn \\ \delta xn = \dfrac{\partial wn}{\partial sn}\delta sn \end{cases} \tag{118}$$

Plugging the two last equations into the first, we have:

$$\frac{d}{dt}(\delta sn) = \left(\frac{\partial fn}{\partial sn} + \frac{\partial fn}{\partial rn}\frac{\partial hn}{\partial xn}\frac{\partial wn}{\partial sn} \right) \delta sn \tag{119}$$

Considering that

$$\frac{\partial fn}{\partial sn} = -\beta^E - \alpha^E T_{glu} rn^* \tag{120}$$

$$\frac{\partial fn}{\partial rn} = \alpha^E T_{glu}(1 - sn^*) \tag{121}$$

$$\frac{\partial wn}{\partial sn} = W_+ J_{nmda} \tag{122}$$

$$\tag{123}$$

We can calculate:

$$\frac{d}{dt}(\delta sn) \tag{124}$$

$$= \left[-(\beta^E + \alpha^E T_{glu} rn^*) + \alpha^E T_{glu}(1 - sn^*) hn'(xn^*) W_+ J_{nmda}\right] \delta sn \tag{125}$$

$$\equiv \lambda_n \delta sn \tag{126}$$

The sign of the real part of λ_n indicates the stability of the fixed point. λ_n is real then if $\lambda_n < 0$, the fixed point is stable and if $\lambda_n > 0$ the fixed point is unstable.

if $sn^* \neq 0$, $\alpha^E T_{glu}(1 - sn^*) hn'(xn^*) > 0$, then:

$$\lambda_n = -(\beta^E + \alpha^E T_{glu} rn^*) + \alpha^E T_{glu}(1 - sn^*) hn'(xn^*) W_+ J_{nmda} < 0 \tag{127}$$

$$\iff -\frac{\beta^E + \alpha^E T_{glu} rn^*}{\alpha^E T_{glu}(1 - sn^*) hn'(xn^*)} + W_+ J_{nmda} < 0 \tag{128}$$

$$\iff \frac{\beta^E + \alpha^E T_{glu} rn^*}{\alpha^E T_{glu}(1 - sn^*) hn'(xn^*)} - W_+ J_{nmda} > 0 \tag{129}$$

We have shown in section 1.2 that the sensitivity of the fixed point with respect to the forcing z_E is written as:

$$\mathscr{P}_{sn,z_E} = \left(\frac{\beta^E + \alpha^E T_{glu} hn(wn(sn^*, z_E))}{\alpha^E T_{glu}(1-sn^*)hn'(wn(sn^*, z_E))} - W_+ J_{nmda}\right)^{-1} = \left(\frac{\beta^E + \alpha^E T_{glu} rn^*}{\alpha^E T_{glu}(1-sn^*)hn'(xn^*)} - W_+ J_{nmda}\right)^{-1} \tag{130}$$

So we have

$$\lambda_n < 0 \Longleftrightarrow \mathscr{P}^{-1}_{sn,z_E} > 0 \tag{131}$$

We show in Fig **??** that, with the tested sets of parameters, the sensitivity of the excitatory pool with autocoupling to a perturbation of the forcing z_E, i.e. $\mathscr{P}_{sn,z_E}$, is strictly positive. We deduce that $\mathscr{P}^{-1}_{sn,z_E}$ is also strictly positive.

Thus, $\lambda_n < 0$ for the tested sets of parameters. The fixed point is stable.

4.1.2 Inhibitory pool

The dynamic system reads:

$$\begin{cases} \dfrac{dsg(t)}{dt} = -\beta^I sg(t) + \alpha^I T_{gaba}(1 - sg(t))rg(t) \equiv fg(sg(t), rg(t)) \\ rg(t) = \dfrac{a_I xg(t) - b_I}{1 - e^{-d_I(a_I xg(t) - b_I)}} \equiv hg(xg(t)) \\ xg(t) = -J_- sg(t) + z_I \equiv wg(sg(t), z_I) \end{cases} \tag{132}$$

We stand near the fixed point: $sg = sg^* + \delta sg$.

With the perturbation of δsg, we get the new model:

$$\begin{cases} \dfrac{d}{dt}(sg^* + \delta sg) = fg(sg^* + \delta sg, rg(t)) \\ rg(t) = hg(xg(t)) \\ xg(t) = wn(sg(t), z_I) \end{cases} \tag{133}$$

By linearization near the fixed point, we get:

$$\begin{cases} \frac{d}{dt}(\delta sg) = \frac{\partial fg}{\partial sg}\delta sg + \frac{\partial fg}{\partial rg}\delta rg \\ \delta rg = \frac{\partial hg}{\partial xg}\delta xg \\ \delta xg = \frac{\partial wg}{\partial sg}\delta sg \end{cases} \quad (134)$$

Plugging the two last equations into the first, we have:

$$\frac{d}{dt}(\delta sg) = \left(\frac{\partial fg}{\partial sg} + \frac{\partial fg}{\partial rg}\frac{\partial hg}{\partial xg}\frac{\partial wg}{\partial sg} \right) \delta sg \quad (135)$$

Considering that

$$\frac{\partial fg}{\partial sg} = -\beta^I - \alpha^I T_{gaba} rg^* \quad (136)$$

$$\frac{\partial fg}{\partial rg} = \alpha^I T_{gaba}(1 - sg^*) \quad (137)$$

$$\frac{\partial wg}{\partial sg} = -J_- \quad (138)$$

$$(139)$$

We can calculate:

$$\frac{d}{dt}(\delta sg) = \left[-(\beta^I + \alpha^I T_{gaba} rg^*) - \alpha^I T_{gaba}(1 - sg^*) hg'(xg^*) J_- \right] \delta sg \quad (140)$$

$$\equiv \lambda_g \delta sg \quad (141)$$

The sign of the real part of λ_g indicates the stability of the fixed point. λ_g is real then if $\lambda_g < 0$, the fixed point is stable and if $\lambda_g > 0$ the fixed point is unstable.

All the terms of λ_g are negative, so $\lambda_g < 0$ and the fixed point is always stable.

4.2 For one isolated area using Open Loop Sensitivities

4.2.1 Dynamic model

The dynamic system can be expressed as:

$$\begin{cases} \dfrac{dsn(t)}{dt} = -\beta^E sn(t) + \alpha^E T_{glu}(1 - sn(t))rn(t) & \equiv fn(sn(t), rn(t)) \\ \dfrac{dsg(t)}{dt} = -\beta^I sg(t) + \alpha^I T_{gaba}(1 - sg(t))rg(t) & \equiv fg(sn(t), rn(t)) \end{cases} \tag{142}$$

with

$$\begin{cases} rn(t) = \dfrac{a_E xn(t) - b_E}{1 - e^{-d_E(a_E xn(t) - b_E)}} & \equiv hn(xn(t)) \\ rg(t) = \dfrac{a_I xg(t) - b_I}{1 - e^{-d_I(a_I xg(t) - b_I)}} & \equiv hg(xg(t)) \end{cases} \tag{143}$$

In Eq 143, $xn(t)$ and $xg(t)$ represent the respective total input current:

$$\begin{cases} xn(t) = W_+ J_{nmda} sn(t) - J_{gaba} sg(t) + x_E \\ xg(t) = J_{nmda} sn(t) - J_- sg(t) + x_I \end{cases} \tag{144}$$

where x_E and x_I represent basal forcings (effective external inputs).

4.2.2 Splitting x_{inter} from x_{intra}

In section 2, in order to express closed loop sensitivities of the area as a function of its open loop sensitivities, we explicitly split this total input current between an internal current within an area (due to intra-pool recurrence and coupling between pools) and an external current (due to the external inputs and the coupling between the two areas). Here, we can do the same in ordre to write the propagation matrix as a function of these open loop sensitivities:

$$\begin{cases} xn_{intra}(t) = W_+ J_{nmda} sn(t) \equiv wn_{intra}(sn(t)) \\ xg_{intra}(t) = -J_- sg(t) \equiv wg_{intra}(sg(t)) \end{cases} \tag{145}$$

$$\begin{cases} xn_{inter}(t) = -J_{gaba} sg(t) + x_E \equiv wn_{inter}(sg(t), x_E) \\ xg_{inter}(t) = J_{nmda} sn(t) + x_I \equiv wg_{inter}(sn(t), x_I) \end{cases} \tag{146}$$

so that total input currents reads:

$$\begin{cases} xn(t) = xn_{intra}(t) + xn_{inter}(t) \equiv wn(xn_{intra}(t), xn_{inter}(t)) \\ xg(t) = xg_{intra}(t) + xg_{inter}(t) \equiv wg(xg_{intra}(t), xg_{inter}(t)) \end{cases} \tag{147}$$

4.2.3 Intermediate variables elimination

We define

$$\vec{s}(t) = \begin{pmatrix} sn(t) \\ sg(t) \end{pmatrix}, \quad \vec{r}(t) = \begin{pmatrix} rn(t) \\ rg(t) \end{pmatrix}, \quad \vec{x}(t) = \begin{pmatrix} xn(t) \\ xg(t) \end{pmatrix}, \quad \overrightarrow{x_{intra}}(t) = \begin{pmatrix} xn_{intra}(t) \\ xg_{intra}(t) \end{pmatrix}, \quad \overrightarrow{x_{inter}}(t) = \begin{pmatrix} xn_{inter}(t) \\ xg_{inter}(t) \end{pmatrix} \tag{148}$$

The dynamic model can thus be rewritten in vectorised form:

$$\begin{cases} \frac{d\vec{s}}{dt} = \vec{f}(\vec{s}, \vec{r}) \\ \vec{r} = \vec{h}(\vec{x}) \\ \vec{x} = \vec{w}(\overrightarrow{x_{intra}}, \overrightarrow{x_{inter}}) \\ \overrightarrow{x_{intra}} = \overrightarrow{w_{intra}}(\vec{s}) \\ \overrightarrow{x_{inter}} = \overrightarrow{w_{inter}}(\vec{s}, x_E, x_I) \end{cases} \tag{149}$$

Plugging intermediate variables $\vec{r}$, $\vec{x}$, $\overrightarrow{x_{intra}}$ into the first equation, Eq 149 can be rewritten as:

$$\begin{cases} \dfrac{d\vec{s}}{dt} = \vec{F}(\vec{s}, \overrightarrow{x_{inter}}) \\ \overrightarrow{x_{inter}} = \overrightarrow{w_{inter}}(\vec{s}, x_E, x_I) \end{cases} \tag{150}$$

where

$$\begin{cases} Fn(sn(t), xn_{inter}(t)) \equiv -\beta^E sn(t) + \alpha^E T_{glu}(1 - sn(t)) \dfrac{a_E(W_+ J_{nmda} sn(t) + xn_{inter}(t)) - b_E}{1 - e^{-d_E[a_E(W_+ J_{nmda} sn(t) + xn_{inter}(t)) - b_E]}} \\ Fg(sg(t), xg_{inter}(t)) = -\beta^I sg(t) + \alpha^I T_{gaba}(1 - sg(t)) \dfrac{a_I(-J_- sg(t) + xg_{inter}(t)) - b_I}{1 - e^{-d_I[a_I(-J_- sg(t) + xg_{inter}(t)) - b_I]}} \end{cases} \tag{151}$$

and where $\overrightarrow{x_{inter}}$, given by Eq 146, will be the support of information transfer between the two pools (hence, denoted *transfer variables*).

4.2.4 Expressing the propagation matrix as a function of Open Loop Sensitivities

We stand near the fixed point: $\vec{s} = \vec{s^*} + \vec{\delta s}$.

The perturbed form of system 150 reads:

$$\begin{cases} \dfrac{d}{dt}(\vec{s^*} + \vec{\delta s}) = \vec{F}(\vec{s^*} + \vec{\delta s}, \overrightarrow{x_{inter}}) \\ \overrightarrow{x_{inter}} = \overrightarrow{w_{inter}}(\vec{s^*} + \vec{\delta s}, x_E, x_I) \end{cases} \tag{152}$$

By linearization near the fixed point, we get:

$$\begin{cases} \dfrac{d}{dt}(\vec{\delta s}) = \overline{\overline{\dfrac{\partial F}{\partial s}}}^* \vec{\delta s} + \overline{\overline{\dfrac{\partial F}{\partial x_{inter}}}}^* \overrightarrow{\delta x_{inter}} \\ \overrightarrow{\delta x_{inter}} = \overline{\overline{\dfrac{\partial w_{inter}}{\partial s}}}^* \vec{\delta s} \end{cases} \tag{153}$$

Plugging the second equation into the first, we have:

$$
\begin{aligned}
\frac{d}{dt}(\overrightarrow{\delta s}) &= \overline{\overline{\frac{\partial F}{\partial s}^*}}\,\overrightarrow{\delta s} + \overline{\overline{\frac{\partial F}{\partial x_{inter}}^* \frac{\partial w_{inter}}{\partial s}^*}}\,\overrightarrow{\delta s} && (154)\\
&= \left(\overline{\overline{\frac{\partial F}{\partial s}^*}} + \overline{\overline{\frac{\partial F}{\partial x_{inter}}^* \frac{\partial w_{inter}}{\partial s}^*}} \right) \overrightarrow{\delta s} && (155)\\
&= \overline{\overline{\frac{\partial F}{\partial s}^*}} \left[\overline{\overline{\mathbb{I}}} - \left(- \overline{\overline{\frac{\partial F}{\partial s}^*}}^{-1} \overline{\overline{\frac{\partial F}{\partial x_{inter}}^*}} \right) \overline{\overline{\frac{\partial w_{inter}}{\partial s}^*}} \right] \overrightarrow{\delta s} && (156)
\end{aligned}
$$

In section 2, where we have written the sensitivities of the fixed point with respect to the forcings x_E and x_I splitting between pools, we have set $\overline{\overline{S}}$ as:

$$
\overline{\overline{S}} = - \overline{\overline{\frac{\partial F}{\partial s}^*}}^{-1} \overline{\overline{\frac{\partial F}{\partial x_{inter}}^*}} \tag{157}
$$

We have shown that $\overline{\overline{S}}$ contains the open loop sensitivities of the area, i.e. the sensitivities of the pools w.r.t. forcings (denoted $\mathscr{A}^O$), evaluated at the fixed point of the closed loop system:

$$
\overline{\overline{S}} = \begin{pmatrix} \mathscr{A}^O_{sn,x_E} & 0 \\ 0 & \mathscr{A}^O_{sg,x_I} \end{pmatrix} \tag{158}
$$

with

$$
\begin{cases}
\mathscr{A}^O_{sn,x_E} = \varphi n_{_E}(sn^*, z_E = -J_{gaba} sg^* + x_E) \\
\mathscr{A}^O_{sg,x_I} = \varphi g_{_I}(sg^*, z_I = J_{nmda} sn^* + x_I)
\end{cases} \tag{159}
$$

where $\varphi n_{_E}$ and $\varphi g_{_I}$ are given by Eq 25 and are to be evaluated at the fixed points sn^* and sg^* yielded by the closed loop system, and taking into account the total amount of external forcing.

Furthermore, from 157 we have:

$$
\overline{\overline{\frac{\partial F}{\partial s}^*}}^{-1} = - \overline{\overline{S}}\, \overline{\overline{\frac{\partial F}{\partial x_{inter}}^*}}^{-1} \tag{160}
$$

and so we can deduce:

$$\overline{\overline{\frac{\partial F}{\partial s}}}^* = -\left(\overline{\overline{S}}\,\overline{\overline{\frac{\partial F}{\partial x_{inter}}}}^{*\,-1}\right)^{-1} = -\overline{\overline{\frac{\partial F}{\partial x_{inter}}}}^*\,\overline{\overline{S}}^{-1} \tag{161}$$

with:

$$\overline{\overline{\frac{\partial F}{\partial x_{inter}}}}^* = \overline{\overline{\frac{\partial f}{\partial r}}}^*\overline{\overline{\frac{\partial h}{\partial x}}}^*\underbrace{\overline{\overline{\frac{\partial w}{\partial x_{inter}}}}^*}_{\overline{\overline{\mathbb{I}}}} \tag{162}$$

Taking equation 156 and substituting, we have:

$$\begin{aligned}
\frac{d}{dt}(\overrightarrow{\delta s}) &= \overline{\overline{\frac{\partial F}{\partial s}}}^*\left(\overline{\overline{\mathbb{I}}} - \overline{\overline{S}}\,\overline{\overline{\frac{\partial w_{inter}}{\partial s}}}^*\right)\overrightarrow{\delta s} && (163)\\
&= -\overline{\overline{\frac{\partial F}{\partial x_{inter}}}}^*\,\overline{\overline{S}}^{-1}\left(\overline{\overline{\mathbb{I}}} - \overline{\overline{S}}\,\overline{\overline{\frac{\partial w_{inter}}{\partial s}}}^*\right)\overrightarrow{\delta s} && (164)\\
&= -\overline{\overline{\frac{\partial F}{\partial x_{inter}}}}^*\left(\overline{\overline{S}}^{-1} - \overline{\overline{\frac{\partial w_{inter}}{\partial s}}}^*\right)\overrightarrow{\delta s} && (165)\\
&= -\overline{\overline{\frac{\partial f}{\partial r}}}^*\overline{\overline{\frac{\partial h}{\partial x}}}^*\left(\overline{\overline{S}}^{-1} - \overline{\overline{\frac{\partial w_{inter}}{\partial s}}}^*\right)\overrightarrow{\delta s} && (166)
\end{aligned}$$

We denote $\overline{\overline{J}}$ the propagation matrix, whose eigenvalues we want to know in order to study the stability of the fixed point $\vec{s}^*$.

$$\overline{\overline{J}} \equiv -\overline{\overline{\frac{\partial f}{\partial r}}}^*\overline{\overline{\frac{\partial h}{\partial x}}}^*\left(\overline{\overline{S}}^{-1} - \overline{\overline{\frac{\partial w_{inter}}{\partial s}}}^*\right) \tag{167}$$

This propagation matrix is written as a function of the open loop sensitivities of the area, i.e. the sensitivities of the pools w.r.t. forcings, evaluated at the fixed point of the area.

We have:

$$\overline{\overline{\frac{\partial f}{\partial r}}}^* = \begin{bmatrix} \frac{\partial fn}{\partial rn}^* & 0 \\ 0 & \frac{\partial fg}{\partial rg}^* \end{bmatrix} = \begin{pmatrix} \alpha^E T_{glu}(1 - sn^*) & 0 \\ 0 & \alpha^I T_{gaba}(1 - sg^*) \end{pmatrix} \tag{168}$$

$$\overline{\overline{\frac{\partial h}{\partial x}}}^* = \begin{bmatrix} \frac{\partial hn}{\partial xn}^* & 0 \\ 0 & \frac{\partial hg}{\partial xg} \end{bmatrix} = \begin{bmatrix} hn'(xn^*) & 0 \\ 0 & hg'(xg^*) \end{bmatrix} \tag{169}$$

$$\overline{\overline{\frac{\partial w_{inter}}{\partial s}}} = \begin{bmatrix} \frac{\partial wn_{inter}}{\partial sn} & \frac{\partial wn_{inter}}{\partial sg} \\ \frac{\partial wg_{inter}}{\partial sn} & \frac{\partial wg_{inter}}{\partial sg} \end{bmatrix} = \begin{bmatrix} 0 & -J_{gaba} \\ J_{nmda} & 0 \end{bmatrix} \tag{170}$$

$$\overline{\overline{S}}^{-1} = \begin{pmatrix} (\mathscr{A}^O_{sn,x_E})^{-1} & 0 \\ 0 & (\mathscr{A}^O_{sg,x_I})^{-1} \end{pmatrix} \tag{171}$$

Thus, we can calculate the propagation matrix:

$$\overline{\overline{J}} = -\overline{\overline{\frac{\partial f}{\partial r}^* \frac{\partial h}{\partial x}^*}} \left(\overline{\overline{S}}^{-1} - \overline{\overline{\frac{\partial w_{inter}}{\partial s}^*}} \right) \tag{172}$$

$$= - \begin{bmatrix} \alpha^E T_{glu}(1 - sn^*) hn'(xn^*) & 0 \\ 0 & \alpha^I T_{gaba}(1 - sg^*) hg'(xg^*) \end{bmatrix} \begin{bmatrix} (\mathscr{A}^O_{sn,x_E})^{-1} & J_{gaba} \\ -J_{nmda} & (\mathscr{A}^O_{sg,x_I})^{-1} \end{bmatrix} \tag{173}$$

$$= - \begin{bmatrix} \alpha^E T_{glu}(1 - sn^*) hn'(xn^*) (\mathscr{A}^O_{sn,x_E})^{-1} & \alpha^E T_{glu}(1 - sn^*) hn'(xn^*) J_{gaba} \\ -\alpha^I T_{gaba}(1 - sg^*) hg'(xg^*) J_{nmda} & \alpha^I T_{gaba}(1 - sg^*) hg'(xg^*) (\mathscr{A}^O_{sg,x_I})^{-1} \end{bmatrix} \tag{174}$$

$$= \begin{bmatrix} -\alpha^E T_{glu}(1 - sn^*) hn'(xn^*) (\mathscr{A}^O_{sn,x_E})^{-1} & -\alpha^E T_{glu}(1 - sn^*) hn'(xn^*) J_{gaba} \\ \alpha^I T_{gaba}(1 - sg^*) hg'(xg^*) J_{nmda} & -\alpha^I T_{gaba}(1 - sg^*) hg'(xg^*) (\mathscr{A}^O_{sg,x_I})^{-1} \end{bmatrix} \tag{175}$$

We have an analytical expression of the propagation matrix $\overline{\overline{J}}$ as a function of the open loop sensitivities of the area, i.e. the sensitivities of the pools. We are interested in its eigenvalues. If all

the real parts of the eigenvalues are negative, then the fixed point is stable. If there is at least one eigenvalue whose the real part is positive, then the fixed point is unstable. Here, all the real parts of eigenvalues are negative.

4.3 For two coupled areas using Open Loop Sensitivities

4.3.1 Dynamic model

The dynamic system can be expressed as:

$$\begin{cases} \dfrac{dsn_i(t)}{dt} = -\beta^E sn_i(t) + \alpha^E T_{glu}(1 - sn_i(t))rn_i(t) & \equiv fn_i(sn_i(t), rn_i(t)) \\ \dfrac{dsg_i(t)}{dt} = -\beta^I sg_i(t) + \alpha^I T_{gaba}(1 - sg_i(t))rg_i(t) & \equiv fg_i(sn_i(t), rn_i(t)) \end{cases} \tag{176}$$

with

$$\begin{cases} rn_i(t) = \dfrac{a_E xn_i(t) - b_E}{1 - e^{-d_E(a_E xn_i(t) - b_E)}} & \equiv hn_i(xn_i(t)) \\ rg_i(t) = \dfrac{a_I xg_i(t) - b_I}{1 - e^{-d_I(a_I xg_i(t) - b_I)}} & \equiv hg_i(xg_i(t)) \end{cases} \tag{177}$$

where $i \in \{1, 2\}$.

In Eq 177, $xn_i(t)$ and $xg_i(t)$ represent the respective total input current to area i.

$$\begin{cases} xn_i(t) = W_+ J_{nmda} sn_i(t) - J_{gaba_i} sg_i(t) + B_{E_i} + k_{E_{ij}} \kappa_{ij} sn_j(t) & j \neq i \\ xg_i(t) = J_{nmda} sn_i(t) - J_- sg_i(t) + B_{I_i} + (1 - k_{E_{ij}})\kappa_{ij} sn_j(t) & j \neq i \end{cases} \tag{178}$$

where B_{E_i} and B_{I_i} represent basal forcings (effective external inputs).

4.3.2 Splitting x_{inter} from x_{intra}

In section 3, in order to express closed loop sensitivities of the areas as a function of their open loop sensitivities, we explicitly split this total input current between an internal current within an area (due to intra-pool recurrence and coupling between pools) and an external current (due to the external inputs and the coupling between the two areas). Here, we can do the same in ordre to write the propagation matrix as a function of these open loop sensitivities:

$$\begin{cases} xn_{intra,i}(t) = W_+ J_{nmda} sn_i(t) - J_{gaba_i} sg_i(t) & \equiv wn_{intra,i}(sn_i(t), sg_i(t)) \\ xg_{intra,i}(t) = J_{nmda} sn_i(t) - J_- sg_i(t) & \equiv wg_{intra,i}(sn_i(t), sg_i(t)) \end{cases} \tag{179}$$

$$\begin{cases} xn_{inter,i}(t) = k_{E_{ij}} \kappa_{ij} sn_j(t) + B_{E_i} & \equiv wn_{inter,i}(sn(t)_j) \quad j \neq i \\ xg_{inter,i}(t) = (1 - k_{E_{ij}}) \kappa_{ij} sn_j(t) + B_{I_i} & \equiv wg_{inter,i}(sn(t)_j) \quad j \neq i \end{cases} \tag{180}$$

so that total input currents reads:

$$\begin{cases} xn_i(t) = xn_{intra,i}(t) + xn_{inter,i}(t) \equiv wn_i(xn_{intra,i}(t), xn_{inter,i}(t)) \\ xg_i(t) = xg_{intra,i}(t) + xg_{inter,i}(t) \equiv wg_i(xg_{intra,i}(t), xg_{inter,i}(t)) \end{cases} \tag{181}$$

4.3.3 Intermediate variables elimination

We define

$$\vec{s}(t) = \begin{pmatrix} sn_1(t) \\ sn_2(t) \\ sg_1(t) \\ sg_2(t) \end{pmatrix}, \quad \vec{r}(t) = \begin{pmatrix} rn_1(t) \\ rn_2(t) \\ rg_1(t) \\ rg_2(t) \end{pmatrix}, \tag{182}$$

$$\vec{x}(t) = \begin{pmatrix} xn_1(t) \\ xn_2(t) \\ xg_1(t) \\ xg_2(t) \end{pmatrix}, \quad \overrightarrow{x_{intra}}(t) = \begin{pmatrix} xn_{intra,1}(t) \\ xn_{intra,2}(t) \\ xg_{intra,1}(t) \\ xg_{intra,2}(t) \end{pmatrix}, \quad \overrightarrow{x_{inter}}(t) = \begin{pmatrix} xn_{inter,1}(t) \\ xn_{inter,2}(t) \\ xg_{inter,1}(t) \\ xg_{inter,2}(t) \end{pmatrix} \tag{183}$$

and

$$\overrightarrow{B_E} = \begin{pmatrix} B_{E_1} \\ B_{E_2} \end{pmatrix}, \quad \overrightarrow{B_I} = \begin{pmatrix} B_{I_1} \\ B_{I_2} \end{pmatrix} \tag{184}$$

The dynamic model can thus be rewritten in vectorised form:

$$\begin{cases} \dfrac{d\vec{s}}{dt} = \vec{f}(\vec{s}, \vec{r}) \\ \vec{r} = \vec{h}(\vec{x}) \\ \vec{x} = \vec{w}(\overrightarrow{x_{intra}}, \overrightarrow{x_{inter}}) \\ \overrightarrow{x_{intra}} = \overrightarrow{w_{intra}}(\vec{s}) \\ \overrightarrow{x_{inter}} = \overrightarrow{w_{inter}}(\vec{s}, \overrightarrow{B_E}, \overrightarrow{B_I}) \end{cases} \tag{185}$$

Plugging intermediate variables $\vec{r}$, $\vec{x}$, $\overrightarrow{x_{intra}}$ into the first equation, Eq 185 can be rewritten as:

$$\begin{cases} \dfrac{d\vec{s}}{dt} = \vec{F}(\vec{s}, \overrightarrow{x_{inter}}) \\ \overrightarrow{x_{inter}} = \overrightarrow{w_{inter}}(\vec{s}, \overrightarrow{B_E}, \overrightarrow{B_I}) \end{cases} \tag{186}$$

where

$$\begin{cases} Fn_i(sn_i(t), sg_i(t), xn_{inter,i}(t)) = -\beta^E sn_i(t) \\ \qquad + \alpha^E T_{glu}(1 - sn_i(t)) \dfrac{a_E(W_+ J_{nmda} sn_i(t) - J_{gaba_i} sg_i(t) + xn_{inter,i}(t)) - b_E}{1 - e^{-d_E[a_E(W_+ J_{nmda} sn_i(t) - J_{gaba_i} sg_i(t) + xn_{inter,i}(t)) - b_E]}} \\ Fg_i(sn_i(t), sg_i(t), xg_{inter,i}(t)) = -\beta^I sg_i(t) \\ \qquad + \alpha^I T_{gaba}(1 - sg_i(t)) \dfrac{a_I(J_{nmda} sn_i(t) - J_- sg_i(t) + xg_{inter,i}(t)) - b_I}{1 - e^{-d_I[a_I(J_{nmda} sn_i(t) - J_- sg_i(t) + xg_{inter,i}(t)) - b_I]}} \end{cases} \tag{187}$$

and where $\overrightarrow{x_{inter}}$, given by Eq 180, will be the transfer variables.

4.3.4 Expressing the propagation matrix as a function of Open Loop Sensitivities

We stand near the fixed point: $\vec{s} = \vec{s^*} + \vec{\delta s}$.

The perturbed form of system 186 reads:

$$\begin{cases} \dfrac{d}{dt}(\vec{s^*} + \vec{\delta s}) = \vec{F}(\vec{s^*} + \vec{\delta s}, \overrightarrow{x_{inter}}) \\ \overrightarrow{x_{inter}} = \overrightarrow{w_{inter}}(\vec{s^*} + \vec{\delta s}, \overrightarrow{B_E}, \overrightarrow{B_I}) \end{cases} \tag{188}$$

By linearization near the fixed point, we get:

$$\begin{cases} \dfrac{d}{dt}(\vec{\delta s}) = \overline{\overline{\dfrac{\partial F}{\partial s}}}^* \vec{\delta s} + \overline{\overline{\dfrac{\partial F}{\partial x_{inter}}}}^* \overrightarrow{\delta x_{inter}} \\ \overrightarrow{\delta x_{inter}} = \overline{\overline{\dfrac{\partial w_{inter}}{\partial s}}}^* \vec{\delta s} \end{cases} \tag{189}$$

Plugging the second equation into the first, we have:

$$\frac{d}{dt}(\overrightarrow{\delta s}) = \overline{\overline{\frac{\partial F}{\partial s}^*}}\overrightarrow{\delta s} + \overline{\overline{\frac{\partial F}{\partial x_{inter}}^* \frac{\partial w_{inter}}{\partial s}^*}}\overrightarrow{\delta s} \tag{190}$$

$$= \left(\overline{\overline{\frac{\partial F}{\partial s}^*}} + \overline{\overline{\frac{\partial F}{\partial x_{inter}}^* \frac{\partial w_{inter}}{\partial s}^*}}\right)\overrightarrow{\delta s} \tag{191}$$

$$= \overline{\overline{\frac{\partial F}{\partial s}^*}}\left[\overline{\overline{\mathbb{I}}} - \left(-\overline{\overline{\frac{\partial F}{\partial s}^*}}^{-1}\overline{\overline{\frac{\partial F}{\partial x_{inter}}^*}}\right)\overline{\overline{\frac{\partial w_{inter}}{\partial s}^*}}\right]\overrightarrow{\delta s} \tag{192}$$

In section 3, where we have written the sensitivities of the fixed point with respect to the forcings $\overrightarrow{B_E}$ and $\overrightarrow{B_I}$ splitting between the two areas, we have set $\overline{\overline{S}}$ as:

$$\overline{\overline{S}} = -\overline{\overline{\frac{\partial F}{\partial s}^*}}^{-1}\overline{\overline{\frac{\partial F}{\partial x_{inter}}^*}} \tag{193}$$

We have shown that $\overline{\overline{S}}$ contains the the open loop sensitivities of the areas, i.e. the sensitivities of each area w.r.t. forcings (denoted $\mathscr{A}$) evaluated at the fixed point of the closed loop system:

$$\overline{\overline{S}} = \begin{pmatrix} \overline{\overline{\mathscr{A}_{sn,x_E}}} & \overline{\overline{\mathscr{A}_{sn,x_I}}} \\ \overline{\overline{\mathscr{A}_{sg,x_E}}} & \overline{\overline{\mathscr{A}_{sg,x_I}}} \end{pmatrix} = \left(\begin{array}{cc|cc} \mathscr{A}_{sn_1,x_{E_1}} & 0 & \mathscr{A}_{sn_1,x_{I_1}} & 0 \\ 0 & \mathscr{A}_{sn_2,x_{E_2}} & 0 & \mathscr{A}_{sn_2,x_{I_2}} \\ \hline \mathscr{A}_{sg_1,x_{E_1}} & 0 & \mathscr{A}_{sg_1,x_{I_1}} & 0 \\ 0 & \mathscr{A}_{sg_2,x_{E_2}} & 0 & \mathscr{A}_{sg_2,x_{I_2}} \end{array}\right) \tag{194}$$

In the same spirit as in Sec 2.5, these open loop sensitivities of areas can be expressed by the analytical expression of their sensitivities when considered isolated, yet to be evaluated respectively at fixed points $\overrightarrow{s_1}^*$ and $\overrightarrow{s_2}^*$ yielded by the closed loop system and taking into account the total amount of external forcing, so we write:

$$\begin{cases} \mathscr{A}_{sn_i,x_{E_i}} = \Phi n_{_E}(\overrightarrow{s^*} = \overrightarrow{s_i^*}, x_E = xn^*_{inter,i}, x_I = xg^*_{inter,i}) \\ \mathscr{A}_{sg_i,x_{E_i}} = \Phi g_{_E}(\overrightarrow{s^*} = \overrightarrow{s_i^*}, x_E = xn^*_{inter,i}, x_I = xg^*_{inter,i}) \\ \mathscr{A}_{sn_i,x_{I_i}} = \Phi n_{_I}(\overrightarrow{s^*} = \overrightarrow{s_i^*}, x_E = xn^*_{inter,i}, x_I = xg^*_{inter,i}) \\ \mathscr{A}_{sg_i,x_{I_i}} = \Phi g_{_I}(\overrightarrow{s^*} = \overrightarrow{s_i^*}, x_E = xn^*_{inter,i}, x_I = xg^*_{inter,i}) \end{cases} \tag{195}$$

where

$$xn^*_{inter,1} = \kappa_{12} k_{E_{12}} sn^*_2 + B_{E_1} \tag{196}$$

$$xg^*_{inter,1} = \kappa_{12}(1 - k_{E_{12}}) sn^*_2 + B_{I_1} \tag{197}$$

$$xn^*_{inter,2} = \kappa_{21} k_{E_{21}} sn^*_1 + B_{E_2} \tag{198}$$

$$xg^*_{inter,2} = \kappa_{21}(1 - k_{E_{21}}) sn^*_1 + B_{I_2} \tag{199}$$

Analytical expression for the functions $\Phi n_{_E}$, $\Phi g_{_E}$, $\Phi n_{_I}$ and $\Phi g_{_I}$ are explicitly given in section 2.7.

Furthermore, from 193 we have:

$$\overline{\overline{\frac{\partial F}{\partial s}^*}}^{-1} = -\overline{\overline{S}}\,\overline{\overline{\frac{\partial F}{\partial x_{inter}}^*}}^{-1} \tag{200}$$

and so we can deduce:

$$\overline{\overline{\frac{\partial F}{\partial s}^*}} = -\left(\overline{\overline{S}}\,\overline{\overline{\frac{\partial F}{\partial x_{inter}}^*}}^{-1}\right)^{-1} = -\overline{\overline{\frac{\partial F}{\partial x_{inter}}^*}}\,\overline{\overline{S}}^{-1} \tag{201}$$

with:

$$\overline{\overline{\frac{\partial F}{\partial x_{inter}}^*}} = \overline{\overline{\frac{\partial f}{\partial r}^* \frac{\partial h}{\partial x}^*}} \underbrace{\overline{\overline{\frac{\partial w}{\partial x_{inter}}^*}}}_{\overline{\overline{\mathbb{I}}}} \tag{202}$$

Taking equation 192 and substituting, we have:

$$\frac{d}{dt}(\overrightarrow{\delta s}) = \overline{\overline{\frac{\partial F}{\partial s}^*}} \left(\overline{\overline{\mathbb{I}}} - \overline{\overline{S}} \, \overline{\overline{\frac{\partial w_{inter}}{\partial s}^*}} \right) \overrightarrow{\delta s} \tag{203}$$

$$= -\overline{\overline{\frac{\partial F}{\partial x_{inter}}^*}} \, \overline{\overline{S}}^{-1} \left(\overline{\overline{\mathbb{I}}} - \overline{\overline{S}} \, \overline{\overline{\frac{\partial w_{inter}}{\partial s}^*}} \right) \overrightarrow{\delta s} \tag{204}$$

$$= -\overline{\overline{\frac{\partial F}{\partial x_{inter}}^*}} \left(\overline{\overline{S}}^{-1} - \overline{\overline{\frac{\partial w_{inter}}{\partial s}^*}} \right) \overrightarrow{\delta s} \tag{205}$$

$$= -\overline{\overline{\frac{\partial f}{\partial r}^* \frac{\partial h}{\partial x}^*}} \left(\overline{\overline{S}}^{-1} - \overline{\overline{\frac{\partial w_{inter}}{\partial s}^*}} \right) \overrightarrow{\delta s} \tag{206}$$

We denote $\overline{\overline{J}}$ the propagation matrix, whose eigenvalues are needed in order to study the stability of the fixed point $\overrightarrow{s}^*$.

$$\overline{\overline{J}} \equiv -\overline{\overline{\frac{\partial f}{\partial r}^* \frac{\partial h}{\partial x}^*}} \left(\overline{\overline{S}}^{-1} - \overline{\overline{\frac{\partial w_{inter}}{\partial s}^*}} \right) \tag{207}$$

This propagation matrix is written as a function of the open loop sensitivities of the areas, i.e. the sensitivities of each area w.r.t. forcings, evaluated at the fixed point of the two coupled area system.

We have:

$$\overline{\overline{\frac{\partial f}{\partial r}^*}} = \begin{bmatrix} \overline{\overline{\frac{\partial fn}{\partial rn}^*}} & \overline{\overline{0}} \\ \overline{\overline{0}} & \overline{\overline{\frac{\partial fg}{\partial rg}^*}} \end{bmatrix} = \left(\begin{array}{cc|cc} \alpha^E T_{glu}(1-sn_1^*) & 0 & 0 & 0 \\ 0 & \alpha^E T_{glu}(1-sn_2^*) & 0 & 0 \\ \hline 0 & 0 & \alpha^I T_{gaba}(1-sg_1^*) & 0 \\ 0 & 0 & 0 & \alpha^I T_{gaba}(1-sg_2^*) \end{array}\right) \tag{208}$$

$$\overline{\overline{\frac{\partial h}{\partial x}^*}} = \begin{bmatrix} \overline{\overline{\frac{\partial hn}{\partial xn}^*}} & \overline{\overline{0}} \\ \overline{\overline{0}} & \overline{\overline{\frac{\partial hg}{\partial xg}^*}} \end{bmatrix} = \left(\begin{array}{cc|cc} hn_1'(xn_1^*) & 0 & 0 & 0 \\ 0 & hn_2'(xn_2^*) & 0 & 0 \\ \hline 0 & 0 & hg_1'(xg_1^*) & 0 \\ 0 & 0 & 0 & hg_2'(xg_2^*) \end{array}\right) \tag{209}$$

$$\overline{\overline{\frac{\partial w_{inter}}{\partial s}}} = \left(\begin{array}{cc|cc} 0 & k_{E_{12}}\kappa_{12} & 0 & 0 \\ k_{E_{21}}\kappa_{21} & 0 & 0 & 0 \\ \hline 0 & (1-k_{E_{12}})\kappa_{12} & 0 & 0 \\ (1-k_{E_{21}})\kappa_{21} & 0 & 0 & 0 \end{array}\right) \tag{210}$$

Furthermore, we want to calculate the inverse matrix of $\overline{\overline{S}}$. $\overline{\overline{S}}$ is a 2×2 block-defined matrix with diagonal blocks.

$$\overline{\overline{S}} = \begin{pmatrix} \overline{\overline{\mathscr{A}_{sn,x_E}}} & \overline{\overline{\mathscr{A}_{sn,x_I}}} \\ \overline{\overline{\mathscr{A}_{sg,x_E}}} & \overline{\overline{\mathscr{A}_{sg,x_I}}} \end{pmatrix} = \left(\begin{array}{cc|cc} \mathscr{A}_{sn_1,x_{E_1}} & 0 & \mathscr{A}_{sn_1,x_{I_1}} & 0 \\ 0 & \mathscr{A}_{sn_2,x_{E_2}} & 0 & \mathscr{A}_{sn_2,x_{I_2}} \\ \hline \mathscr{A}_{sg_1,x_{E_1}} & 0 & \mathscr{A}_{sg_1,x_{I_1}} & 0 \\ 0 & \mathscr{A}_{sg_2,x_{E_2}} & 0 & \mathscr{A}_{sg_2,x_{I_2}} \end{array}\right) \tag{211}$$

We denote:

$$\overline{\overline{S}}^{-1} \equiv \begin{bmatrix} (\overline{\overline{S}}^{-1})_{11} & (\overline{\overline{S}}^{-1})_{12} \\ (\overline{\overline{S}}^{-1})_{21} & (\overline{\overline{S}}^{-1})_{22} \end{bmatrix} \tag{212}$$

To calculate eack block of $\overline{\overline{S}}^{-1}$, we use the same property as the one exposed in section 3:

Considering the property that

$$\text{If } \overline{\overline{M}} = \begin{pmatrix} \overline{\overline{A}} & \overline{\overline{B}} \\ \overline{\overline{C}} & \overline{\overline{D}} \end{pmatrix} \text{ with } \overline{\overline{D}} \text{ invertible}$$

$$\text{then } \overline{\overline{M}}^{-1} = \begin{pmatrix} (\overline{\overline{A}} - \overline{\overline{BD}}^{-1}\overline{\overline{C}})^{-1} & -\overline{\overline{RBD}}^{-1} \\ -\overline{\overline{D}}^{-1}\overline{\overline{CR}} & \overline{\overline{D}}^{-1}(\overline{\overline{\mathbb{I}}} - \overline{\overline{CS}}) \end{pmatrix}$$

For the first block of $\overline{\overline{S}}^{-1}$, we have:

$$(\overline{\overline{S}}^{-1})_{11} = \left(\overline{\overline{\mathscr{A}_{sn,x_E}}} - \overline{\overline{\mathscr{A}_{sn,x_I}\mathscr{A}_{sg,x_I}}}^{-1}\overline{\overline{\mathscr{A}_{sg,x_E}}}\right)^{-1} \tag{213}$$

$$= \left(\begin{bmatrix} \mathscr{A}_{sn_1,x_{E_1}} & 0 \\ 0 & \mathscr{A}_{sn_2,x_{E_2}} \end{bmatrix} - \begin{bmatrix} \mathscr{A}_{sn_1,x_{I_1}}\mathscr{A}^{-1}_{sg_1,x_{I_1}}\mathscr{A}_{sg_1,x_{E_1}} & 0 \\ 0 & \mathscr{A}_{sn_2,x_{I_2}}\mathscr{A}^{-1}_{sg_2,x_{I_2}}\mathscr{A}_{sg_2,x_{E_2}} \end{bmatrix}\right)^{-1} \tag{214}$$

$$= \begin{bmatrix} \mathscr{A}_{sn_1,x_{E_1}} - \mathscr{A}_{sn_1,x_{I_1}}\mathscr{A}^{-1}_{sg_1,x_{I_1}}\mathscr{A}_{sg_1,x_{E_1}} & 0 \\ 0 & \mathscr{A}_{sn_2,x_{E_2}} - \mathscr{A}_{sn_2,x_{I_2}}\mathscr{A}^{-1}_{sg_2,x_{I_2}}\mathscr{A}_{sg_2,x_{E_2}} \end{bmatrix}^{-1} \tag{215}$$

$$= \begin{bmatrix} \frac{1}{\mathscr{A}_{sn_1,x_{E_1}} - \mathscr{A}_{sn_1,x_{I_1}}\mathscr{A}^{-1}_{sg_1,x_{I_1}}\mathscr{A}_{sg_1,x_{E_1}}} & 0 \\ 0 & \frac{1}{\mathscr{A}_{sn_2,x_{E_2}} - \mathscr{A}_{sn_2,x_{I_2}}\mathscr{A}^{-1}_{sg_2,x_{I_2}}\mathscr{A}_{sg_2,x_{E_2}}} \end{bmatrix} \tag{216}$$

We denote:

$$\begin{cases} \Delta\mathscr{A}_1 = \mathscr{A}_{sn_1,x_{E_1}} - \mathscr{A}_{sn_1,x_{I_1}}\mathscr{A}^{-1}_{sg_1,x_{I_1}}\mathscr{A}_{sg_1,x_{E_1}} \\ \Delta\mathscr{A}_2 = \mathscr{A}_{sn_2,x_{E_2}} - \mathscr{A}_{sn_2,x_{I_2}}\mathscr{A}^{-1}_{sg_2,x_{I_2}}\mathscr{A}_{sg_2,x_{E_2}} \end{cases} \tag{217}$$

So:

$$(\overline{\overline{S}}^{-1})_{11} = \begin{bmatrix} \frac{1}{\Delta\mathscr{A}_1} & 0 \\ 0 & \frac{1}{\Delta\mathscr{A}_2} \end{bmatrix} \tag{218}$$

For the second block of $\overline{\overline{S}}^{-1}$, we have:

$$(\overline{\overline{S}}^{-1})_{12} = -(\overline{\overline{S}}^{-1})_{11}\overline{\overline{\mathscr{A}_{sn,x_I}\mathscr{A}_{sg,x_I}}}^{-1} \tag{219}$$

$$= \begin{bmatrix} -\frac{\mathscr{A}_{sn_1,x_{I_1}}\mathscr{A}_{sg_1,x_{I_1}}^{-1}}{\Delta\mathscr{A}_1} & 0 \\ 0 & -\frac{\mathscr{A}_{sn_2,x_{I_2}}\mathscr{A}_{sg_2,x_{I_2}}^{-1}}{\Delta\mathscr{A}_2} \end{bmatrix} \tag{220}$$

The third block of $\overline{\overline{S}}^{-1}$ is equal to:

$$(\overline{\overline{S}}^{-1})_{21} = -\overline{\overline{\mathscr{A}_{sg,x_I}}}^{-1}\overline{\overline{\mathscr{A}_{sg,x_E}}}(\overline{\overline{S}}^{-1})_{11} \tag{221}$$

$$= \begin{bmatrix} -\frac{\mathscr{A}_{sg_1,x_{I_1}}^{-1}\mathscr{A}_{sg_1,x_{E_1}}}{\Delta\mathscr{A}_1} & 0 \\ 0 & -\frac{\mathscr{A}_{sg_2,x_{I_2}}^{-1}\mathscr{A}_{sg_2,x_{E_2}}}{\Delta\mathscr{A}_2} \end{bmatrix} \tag{222}$$

Finally the last block of $\overline{\overline{S}}^{-1}$ is:

$$(\overline{\overline{S}}^{-1})_{22} = \overline{\overline{\mathscr{A}_{sg,x_I}}}^{-1}\left(\overline{\overline{\mathbb{I}}} - \overline{\overline{\mathscr{A}_{sg,x_E}}}(\overline{\overline{S}}^{-1})_{12}\right) \tag{223}$$

$$= \overline{\overline{\mathscr{A}_{sg,x_I}}}^{-1}\left(\overline{\overline{\mathbb{I}}} - \overline{\overline{\mathscr{A}_{sg,x_E}}}(-(\overline{\overline{S}}^{-1})_{11}\overline{\overline{\mathscr{A}_{sn,x_I}\mathscr{A}_{sg,x_I}}}^{-1})\right) \tag{224}$$

$$= \overline{\overline{\mathscr{A}_{sg,x_I}}}^{-1}\left(\overline{\overline{\mathbb{I}}} + \overline{\overline{\mathscr{A}_{sg,x_E}}}(\overline{\overline{S}}^{-1})_{11}\overline{\overline{\mathscr{A}_{sn,x_I}\mathscr{A}_{sg,x_I}}}^{-1}\right) \tag{225}$$

$$= \begin{bmatrix} \mathscr{A}_{sg_1,x_{I_1}}^{-1} & 0 \\ 0 & \mathscr{A}_{sg_2,x_{I_2}}^{-1} \end{bmatrix} \begin{bmatrix} 1 + \frac{\mathscr{A}_{sg_1,x_{E_1}}\mathscr{A}_{sn_1,x_{I_1}}\mathscr{A}_{sg_1,x_{I_1}}^{-1}}{\Delta\mathscr{A}_1} & 0 \\ 0 & 1 + \frac{\mathscr{A}_{sg_2,x_{E_2}}\mathscr{A}_{sn_2,x_{I_2}}\mathscr{A}_{sg_2,x_{I_2}}^{-1}}{\Delta\mathscr{A}_2} \end{bmatrix} \tag{226}$$

$$= \begin{bmatrix} \mathscr{A}_{sg_1,x_{I_1}}^{-1}\left(1 + \frac{\mathscr{A}_{sg_1,x_{E_1}}\mathscr{A}_{sn_1,x_{I_1}}\mathscr{A}_{sg_1,x_{I_1}}^{-1}}{\Delta\mathscr{A}_1}\right) & 0 \\ 0 & \mathscr{A}_{sg_2,x_{I_2}}^{-1}\left(1 + \frac{\mathscr{A}_{sg_2,x_{E_2}}\mathscr{A}_{sn_2,x_{I_2}}\mathscr{A}_{sg_2,x_{I_2}}^{-1}}{\Delta\mathscr{A}_2}\right) \end{bmatrix} \tag{227}$$

$\overline{\overline{S}}^{-1}$ has the same structure as $\overline{\overline{S}}$. It is a 2×2 block-defined matrix with diagonal blocks.

$$\overline{\overline{S}}^{-1} = \tag{228}$$

$$\left[\begin{array}{cc|cc} \frac{1}{\Delta\mathscr{A}_1} & 0 & -\frac{\mathscr{A}_{sn_1,x_{I_1}}\mathscr{A}^{-1}_{sg_1,x_{I_1}}}{\Delta\mathscr{A}_1} & 0 \\ 0 & \frac{1}{\Delta\mathscr{A}_2} & 0 & -\frac{\mathscr{A}_{sn_2,x_{I_2}}\mathscr{A}^{-1}_{sg_2,x_{I_2}}}{\Delta\mathscr{A}_2} \\ \hline -\frac{\mathscr{A}^{-1}_{sg_1,x_{I_1}}\mathscr{A}_{sg_1,x_{E_1}}}{\Delta\mathscr{A}_1} & 0 & \mathscr{A}^{-1}_{sg_1,x_{I_1}}\left(1+\frac{\mathscr{A}_{sg_1,x_{E_1}}\mathscr{A}_{sn_1,x_{I_1}}\mathscr{A}^{-1}_{sg_1,x_{I_1}}}{\Delta\mathscr{A}_1}\right) & 0 \\ 0 & -\frac{\mathscr{A}^{-1}_{sg_2,x_{I_2}}\mathscr{A}_{sg_2,x_{E_2}}}{\Delta\mathscr{A}_2} & 0 & \mathscr{A}^{-1}_{sg_2,x_{I_2}}\left(1+\frac{\mathscr{A}_{sg_2,x_{E_2}}\mathscr{A}_{sn_2,x_{I_2}}\mathscr{A}^{-1}_{sg_2,x_{I_2}}}{\Delta\mathscr{A}_2}\right) \end{array}\right] \tag{229}$$

We denote:

$$\begin{cases} \alpha^E T_{glu}(1-sn_i^*)hn_i'(xn_i^*) \equiv \xi_{n_i} \\ \alpha^I T_{gaba}(1-sg_i^*)hg_i'(xg_i^*) \equiv \xi_{g_i} \end{cases} \tag{230}$$

So that:

$$-\overline{\overline{\frac{\partial f}{\partial r}^* \frac{\partial h}{\partial x}^*}} = \left(\begin{array}{cc|cc} -\xi_{n_1} & 0 & 0 & 0 \\ 0 & -\xi_{n_2} & 0 & 0 \\ \hline 0 & 0 & -\xi_{g_1} & 0 \\ 0 & 0 & 0 & -\xi_{g_2} \end{array}\right) \tag{231}$$

Furthermore, we have:

$$\overline{\overline{S}}^{-1} - \overline{\overline{\frac{\partial w_{inter}}{\partial s}}}^{*} = \tag{232}$$

$$\left[\begin{array}{cc|cc} \frac{1}{\Delta\mathscr{A}_1} & -k_{E_{12}}\kappa_{12} & -\frac{\mathscr{A}_{sn_1,x_{I_1}}\mathscr{A}_{sg_1,x_{I_1}}^{-1}}{\Delta\mathscr{A}_1} & 0 \\ -k_{E_{21}}\kappa_{21} & \frac{1}{\Delta\mathscr{A}_2} & 0 & -\frac{\mathscr{A}_{sn_2,x_{I_2}}\mathscr{A}_{sg_2,x_{I_2}}^{-1}}{\Delta\mathscr{A}_2} \\ \hline -\frac{\mathscr{A}_{sg_1,x_{I_1}}^{-1}\mathscr{A}_{sg_1,x_{E_1}}}{\Delta\mathscr{A}_1} & -(1-k_{E_{12}})\kappa_{12} & \mathscr{A}_{sg_1,x_{I_1}}^{-1}\left(1+\frac{\mathscr{A}_{sg_1,x_{E_1}}\mathscr{A}_{sn_1,x_{I_1}}\mathscr{A}_{sg_1,x_{I_1}}^{-1}}{\Delta\mathscr{A}_1}\right) & 0 \\ -(1-k_{E_{21}})\kappa_{21} & -\frac{\mathscr{A}_{sg_2,x_{I_2}}^{-1}\mathscr{A}_{sg_2,x_{E_2}}}{\Delta\mathscr{A}_2} & 0 & \mathscr{A}_{sg_2,x_{I_2}}^{-1}\left(1+\frac{\mathscr{A}_{sg_2,x_{E_2}}\mathscr{A}_{sn_2,x_{I_2}}\mathscr{A}_{sg_2,x_{I_2}}^{-1}}{\Delta\mathscr{A}_2}\right) \end{array}\right] \tag{233}$$

Which gives:

$$\overline{\overline{J}} = -\overline{\overline{\frac{\partial f}{\partial r}^{*}\frac{\partial h}{\partial x}^{*}}}\left(\overline{\overline{S}}^{-1} - \overline{\overline{\frac{\partial w_{inter}}{\partial s}}}^{*}\right) \equiv \begin{bmatrix} \overline{\overline{J_{11}}} & \overline{\overline{J_{12}}} \\ \overline{\overline{J_{21}}} & \overline{\overline{J_{22}}} \end{bmatrix} \tag{234}$$

with

$$\overline{\overline{J_{11}}} = \begin{bmatrix} -\xi_{n_1} & 0 \\ 0 & -\xi_{n_2} \end{bmatrix} \begin{bmatrix} \frac{1}{\Delta\mathscr{A}_1} & -k_{E_{12}}\kappa_{12} \\ -k_{E_{21}}\kappa_{21} & \frac{1}{\Delta\mathscr{A}_2} \end{bmatrix} \tag{235}$$

$$= \begin{bmatrix} -\xi_{n_1}\frac{1}{\Delta\mathscr{A}_1} & \xi_{n_1}k_{E_{12}}\kappa_{12} \\ \xi_{n_2}k_{E_{21}}\kappa_{21} & -\xi_{n_2}\frac{1}{\Delta\mathscr{A}_2} \end{bmatrix} \tag{236}$$

$$\overline{\overline{J_{12}}} = \begin{bmatrix} -\xi_{n_1} & 0 \\ 0 & -\xi_{n_2} \end{bmatrix} \begin{bmatrix} -\frac{\mathscr{A}_{sn_1,x_{I_1}}\mathscr{A}_{sg_1,x_{I_1}}^{-1}}{\Delta\mathscr{A}_1} & 0 \\ 0 & -\frac{\mathscr{A}_{sn_2,x_{I_2}}\mathscr{A}_{sg_2,x_{I_2}}^{-1}}{\Delta\mathscr{A}_2} \end{bmatrix} \tag{237}$$

$$= \begin{bmatrix} \xi_{n_1}\frac{\mathscr{A}_{sn_1,x_{I_1}}\mathscr{A}_{sg_1,x_{I_1}}^{-1}}{\Delta\mathscr{A}_1} & 0 \\ 0 & \xi_{n_2}\frac{\mathscr{A}_{sn_2,x_{I_2}}\mathscr{A}_{sg_2,x_{I_2}}^{-1}}{\Delta\mathscr{A}_2} \end{bmatrix} \tag{238}$$

$$\overline{\overline{J_{21}}} = \begin{bmatrix} -\xi_{g_1} & 0 \\ 0 & -\xi_{g_2} \end{bmatrix} \begin{bmatrix} -\frac{\mathscr{A}^{-1}_{sg_1,x_{I_1}} \mathscr{A}_{sg_1,x_{E_1}}}{\Delta\mathscr{A}_1} & -(1-k_{E_{12}})\kappa_{12} \\ -(1-k_{E_{21}})\kappa_{21} & -\frac{\mathscr{A}^{-1}_{sg_2,x_{I_2}} \mathscr{A}_{sg_2,x_{E_2}}}{\Delta\mathscr{A}_2} \end{bmatrix} \tag{239}$$

$$= \begin{bmatrix} \xi_{g_1} \frac{\mathscr{A}^{-1}_{sg_1,x_{I_1}} \mathscr{A}_{sg_1,x_{E_1}}}{\Delta\mathscr{A}_1} & \xi_{g_1}(1-k_{E_{12}})\kappa_{12} \\ \xi_{g_2}(1-k_{E_{21}})\kappa_{21} & \xi_{g_2} \frac{\mathscr{A}^{-1}_{sg_2,x_{I_2}} \mathscr{A}_{sg_2,x_{E_2}}}{\Delta\mathscr{A}_2} \end{bmatrix} \tag{240}$$

$$\overline{\overline{J_{22}}} = \begin{bmatrix} -\xi_{g_1} & 0 \\ 0 & -\xi_{g_2} \end{bmatrix} \begin{bmatrix} \mathscr{A}^{-1}_{sg_1,x_{I_1}} \left(1 + \frac{\mathscr{A}_{sg_1,x_{E_1}} \mathscr{A}_{sn_1,x_{I_1}} \mathscr{A}^{-1}_{sg_1,x_{I_1}}}{\Delta\mathscr{A}_1}\right) & 0 \\ 0 & \mathscr{A}^{-1}_{sg_2,x_{I_2}} \left(1 + \frac{\mathscr{A}_{sg_2,x_{E_2}} \mathscr{A}_{sn_2,x_{I_2}} \mathscr{A}^{-1}_{sg_2,x_{I_2}}}{\Delta\mathscr{A}_2}\right) \end{bmatrix} \tag{241}$$

$$= \begin{bmatrix} -\xi_{g_1} \mathscr{A}^{-1}_{sg_1,x_{I_1}} \left(1 + \frac{\mathscr{A}_{sg_1,x_{E_1}} \mathscr{A}_{sn_1,x_{I_1}} \mathscr{A}^{-1}_{sg_1,x_{I_1}}}{\Delta\mathscr{A}_1}\right) & 0 \\ 0 & -\xi_{g_2} \mathscr{A}^{-1}_{sg_2,x_{I_2}} \left(1 + \frac{\mathscr{A}_{sg_2,x_{E_2}} \mathscr{A}_{sn_2,x_{I_2}} \mathscr{A}^{-1}_{sg_2,x_{I_2}}}{\Delta\mathscr{A}_2}\right) \end{bmatrix} \tag{242}$$

We have an analytical expression of the propagation matrix $\overline{\overline{J}}$ as a function of the open loop sensitivities of the areas, i.e. the sensitivities of each area.

$\overline{\overline{J}}$ is a 2×2 block-defined matrix with two diagonal blocks ($\overline{\overline{J_{12}}}$ and $\overline{\overline{J_{22}}}$).

4.3.5 Propagation matrix in a Control-Target system

We attribute a role to each area: the area 1, receiving a perturbated input current, will be called "Control" area (denoted by C), and the area 2 will be called "Target area" (denoted by T).

We are then interested in the eigenvalues of the following propagation matrix:

$$\overline{\overline{J}} = -\overline{\overline{\frac{\partial f}{\partial r}^* \frac{\partial h}{\partial x}^*}} \left(\overline{\overline{S}}^{-1} - \overline{\overline{\frac{\partial w_{inter}}{\partial s}^*}} \right) \equiv \begin{bmatrix} \overline{\overline{J_{11}}} & \overline{\overline{J_{12}}} \\ \overline{\overline{J_{21}}} & \overline{\overline{J_{22}}} \end{bmatrix} \tag{243}$$

with

$$\begin{cases} \overline{\overline{J_{11}}} = \begin{bmatrix} -\xi_{n_C} \frac{1}{\Delta \mathscr{A}_C} & \xi_{n_C} k_{E_{CT}} \kappa_{CT} \\ \xi_{n_T} k_{E_{TC}} \kappa_{TC} & -\xi_{n_T} \frac{1}{\Delta \mathscr{A}_T} \end{bmatrix} \\ \overline{\overline{J_{12}}} = \begin{bmatrix} \xi_{n_C} \frac{\mathscr{A}_{sn_C, x_{I_C}} \mathscr{A}^{-1}_{sg_C, x_{I_C}}}{\Delta \mathscr{A}_C} & 0 \\ 0 & \xi_{n_T} \frac{\mathscr{A}_{sn_T, x_{I_T}} \mathscr{A}^{-1}_{sg_T, x_{I_T}}}{\Delta \mathscr{A}_T} \end{bmatrix} \\ \overline{\overline{J_{21}}} = \begin{bmatrix} \xi_{g_C} \frac{\mathscr{A}^{-1}_{sg_C, x_{I_C}} \mathscr{A}_{sg_C, x_{E_C}}}{\Delta \mathscr{A}_C} & \xi_{g_C} (1 - k_{E_{CT}}) \kappa_{CT} \\ \xi_{g_T} (1 - k_{E_{TC}}) \kappa_{TC} & \xi_{g_T} \frac{\mathscr{A}^{-1}_{sg_T, x_{I_T}} \mathscr{A}_{sg_T, x_{E_T}}}{\Delta \mathscr{A}_T} \end{bmatrix} \\ \overline{\overline{J_{22}}} = \begin{bmatrix} -\xi_{g_C} \mathscr{A}^{-1}_{sg_C, x_{I_C}} \left(1 + \frac{\mathscr{A}_{sg_C, x_{E_C}} \mathscr{A}_{sn_C, x_{I_C}} \mathscr{A}^{-1}_{sg_C, x_{I_C}}}{\Delta \mathscr{A}_C} \right) & 0 \\ 0 & -\xi_{g_T} \mathscr{A}^{-1}_{sg_T, x_{I_T}} \left(1 + \frac{\mathscr{A}_{sg_T, x_{E_T}} \mathscr{A}_{sn_T, x_{I_T}} \mathscr{A}^{-1}_{sg_T, x_{I_T}}}{\Delta \mathscr{A}_T} \right) \end{bmatrix} \end{cases} \tag{244}$$

Denoting

$$\begin{cases} \alpha^E T_{glu} (1 - sn^*_{C|T}) hn'_{C|T} (xn^*_{C|T}) \equiv \xi_{n_{C|T}} \\ \alpha^I T_{gaba} (1 - sg^*_{C|T}) hg'_{C|T} (xg^*_{C|T}) \equiv \xi_{g_{C|T}} \end{cases} \tag{245}$$

and

$$\begin{cases} \Delta \mathscr{A}_C = \mathscr{A}_{sn_C, x_{E_C}} - \mathscr{A}_{sn_C, x_{I_C}} \mathscr{A}^{-1}_{sg_C, x_{I_C}} \mathscr{A}_{sg_C, x_{E_C}} \\ \Delta \mathscr{A}_T = \mathscr{A}_{sn_T, x_{E_T}} - \mathscr{A}_{sn_T, x_{I_T}} \mathscr{A}^{-1}_{sg_T, x_{I_T}} \mathscr{A}_{sg_T, x_{E_T}} \end{cases} \tag{246}$$

We checked that all the real parts of eigenvalues are negative.
