# Supplementary figures and images for: Sensitivity analysis enlightens effects of connectivity in a Neural Mass Model under Control-Target mode
Source: PLoS Comput Biol. 2026 Mar 23;22(3):e1014035. doi: 10.1371/journal.pcbi.1014035 (PMC13008111; doi:10.1371/journal.pcbi.1014035)

# Connectivity EI

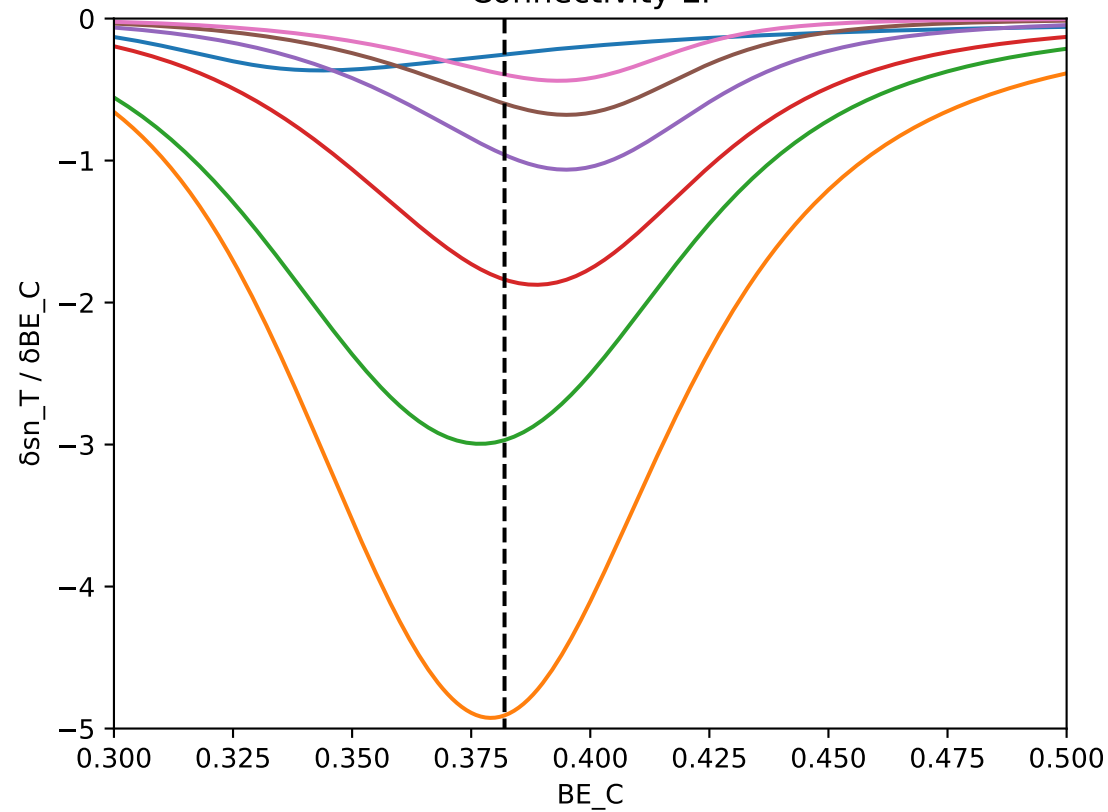

Supplement: S3 File — (ZIP) [file pcbi.1014035.s003.zip › 01-code/11-fig11/Sensi_snT_BEC_EI.pdf]

Connectivity EI

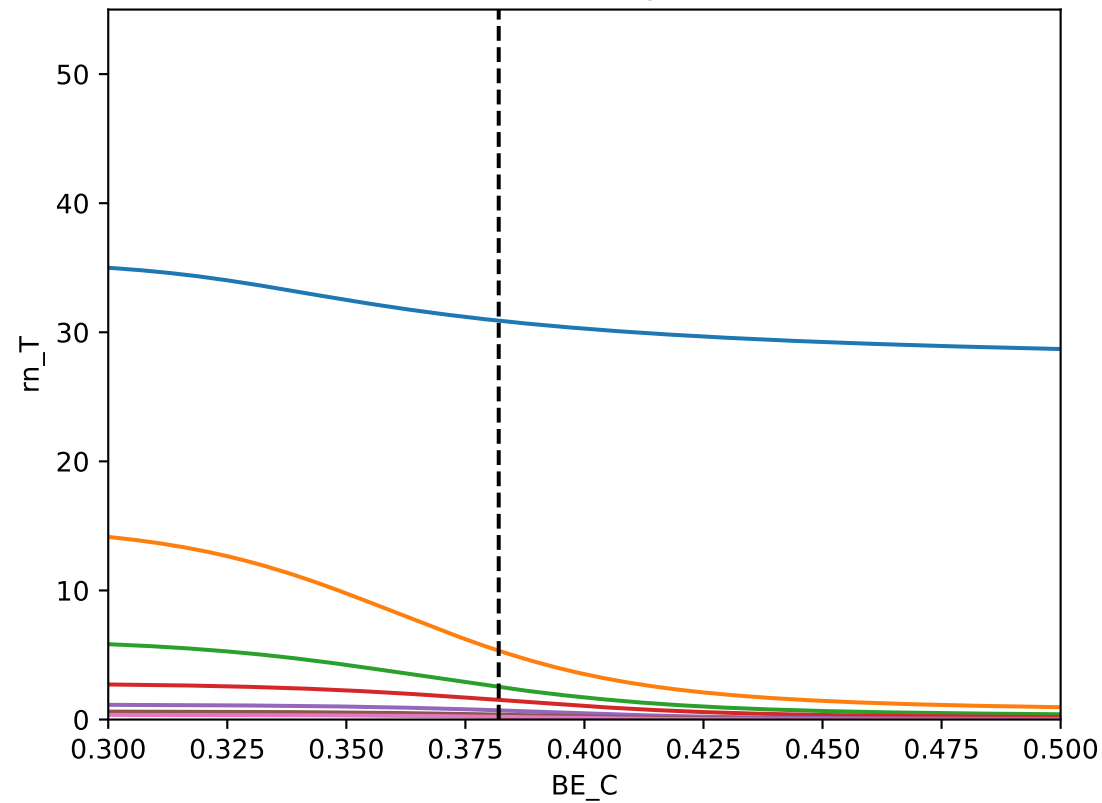

Supplement: S3 File — (ZIP) [file pcbi.1014035.s003.zip › 01-code/11-fig11/rnT_EI.pdf]

# Connectivity EI

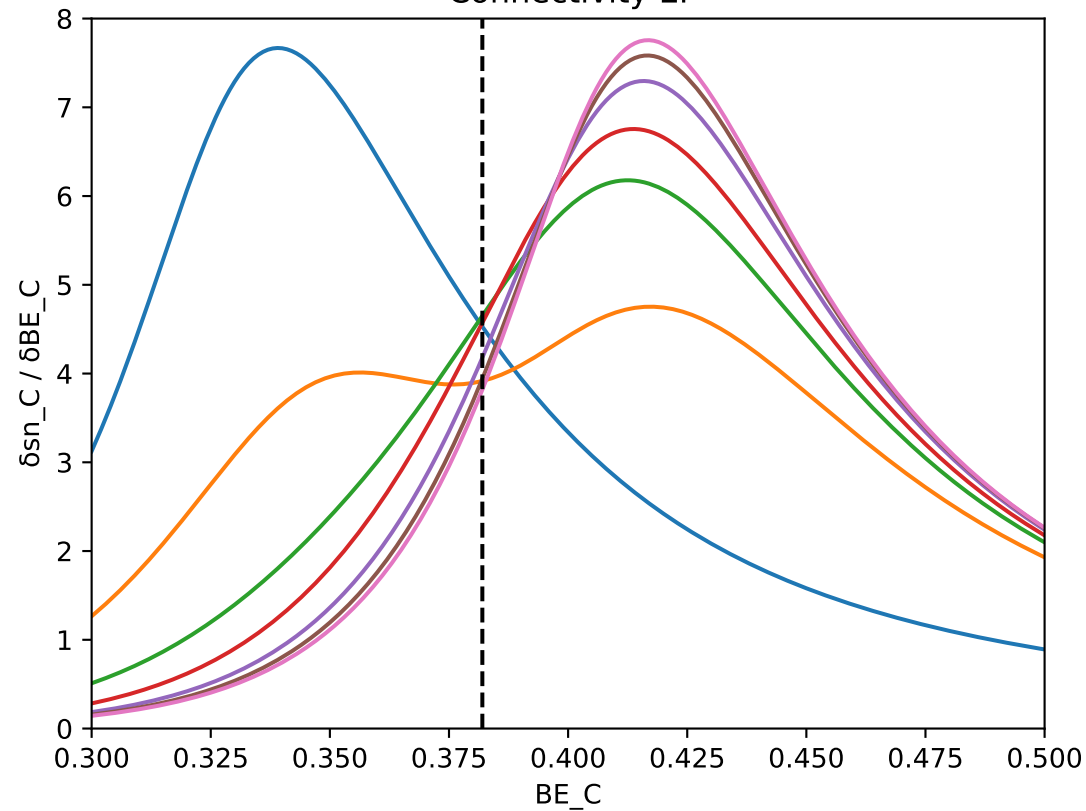

Supplement: S3 File — (ZIP) [file pcbi.1014035.s003.zip › 01-code/11-fig11/Sensi_snC_BEC_EI.pdf]

# Connectivity EI

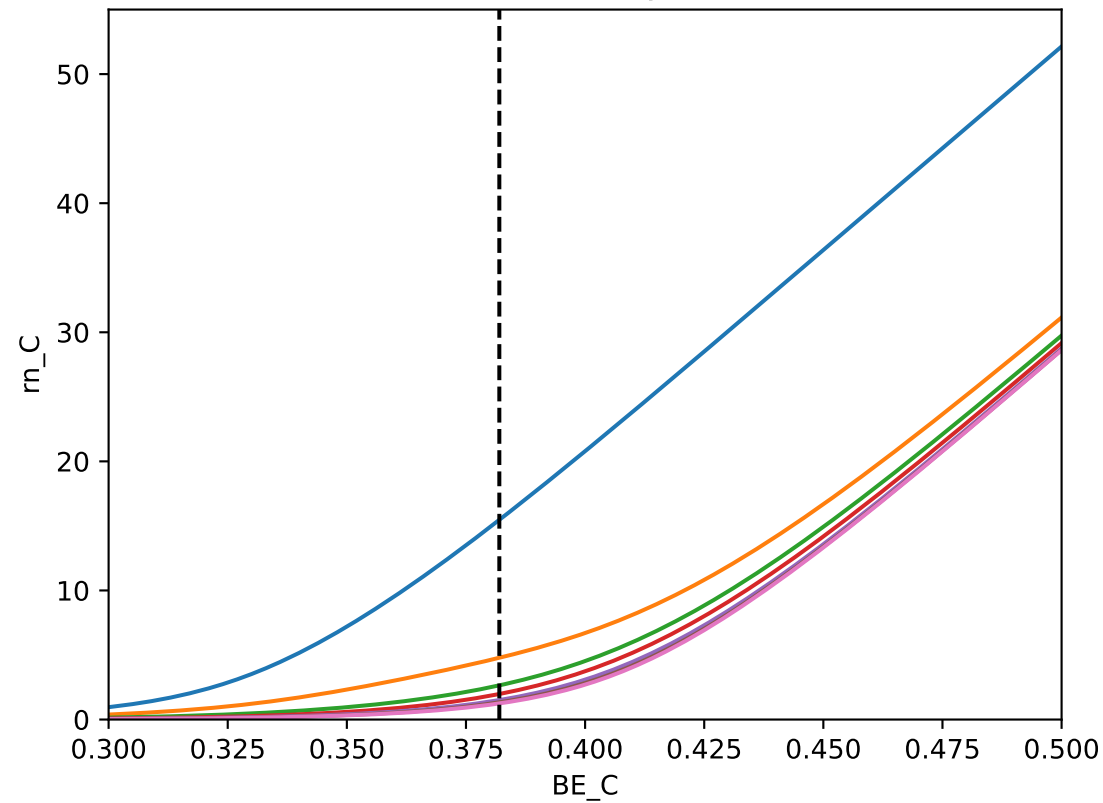

Supplement: S3 File — (ZIP) [file pcbi.1014035.s003.zip › 01-code/11-fig11/rnC_EI.pdf]

# Connectivity EI

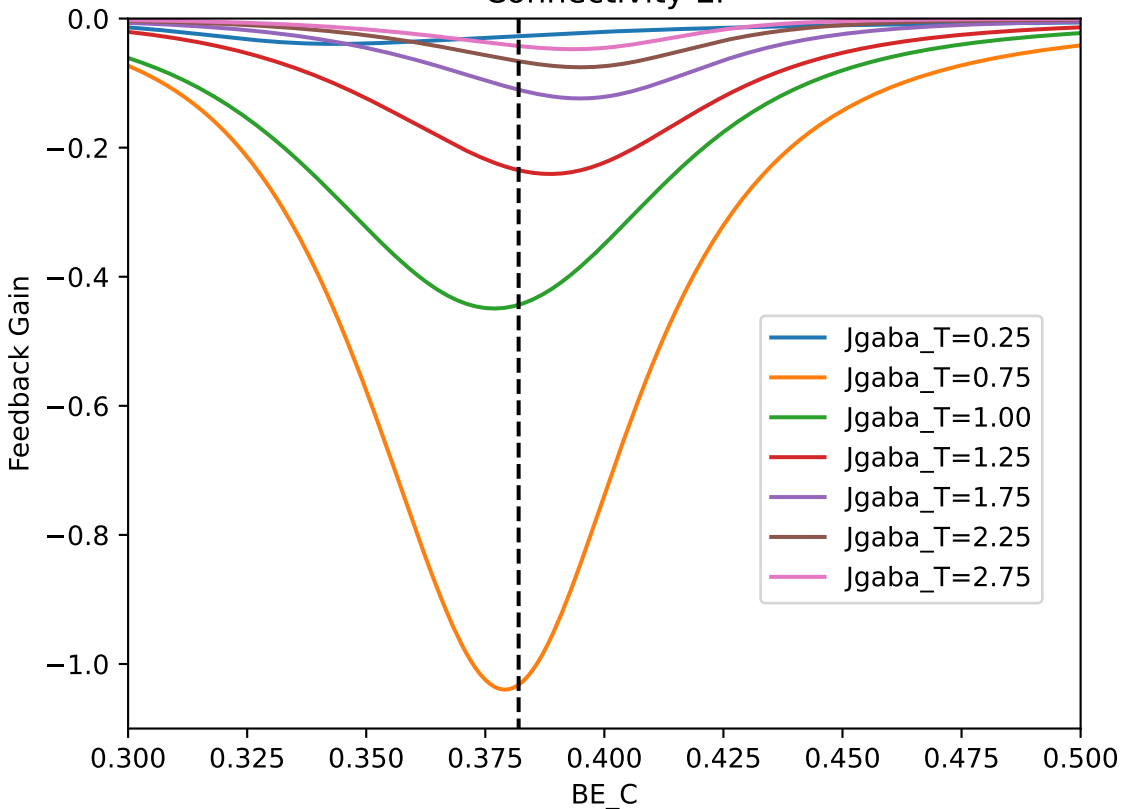

Supplement: S3 File — (ZIP) [file pcbi.1014035.s003.zip › 01-code/11-fig11/FeedbackGain_EI.pdf]

# Connectivity II

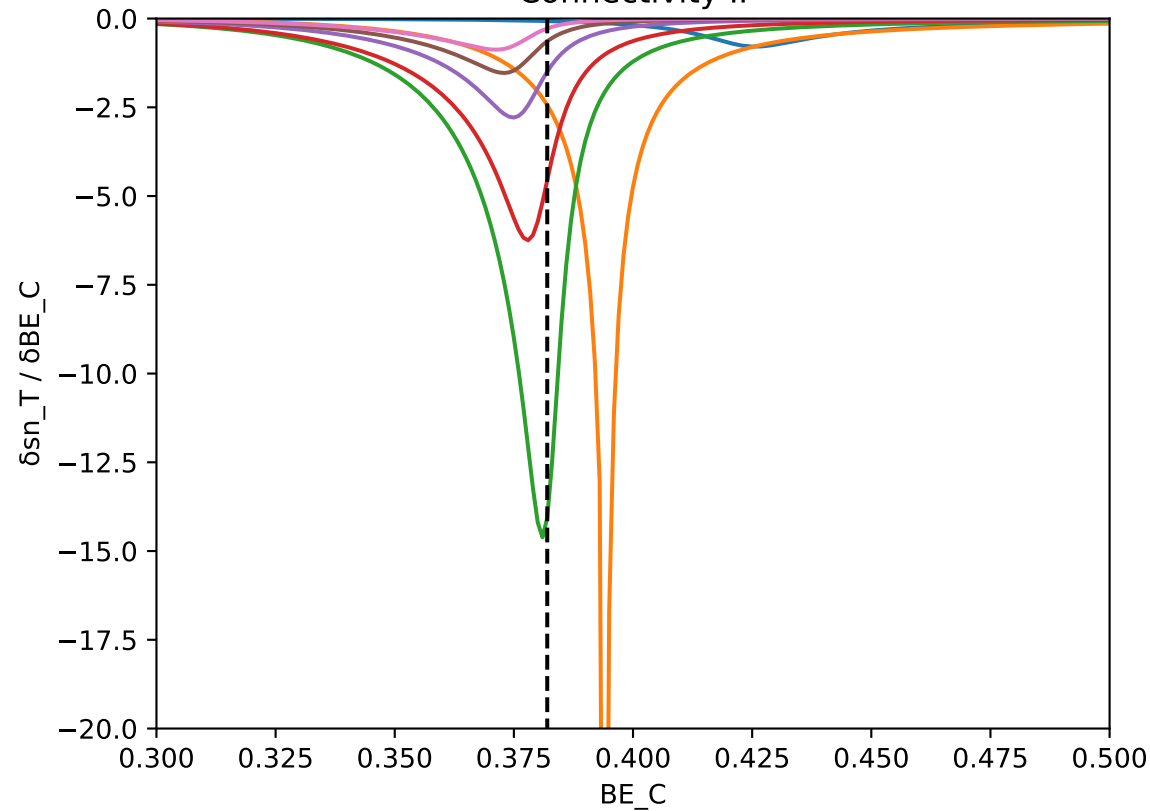

Supplement: S3 File — (ZIP) [file pcbi.1014035.s003.zip › 01-code/10-fig10/Sensi_snT_BEC_II.pdf]

## Connectivity II

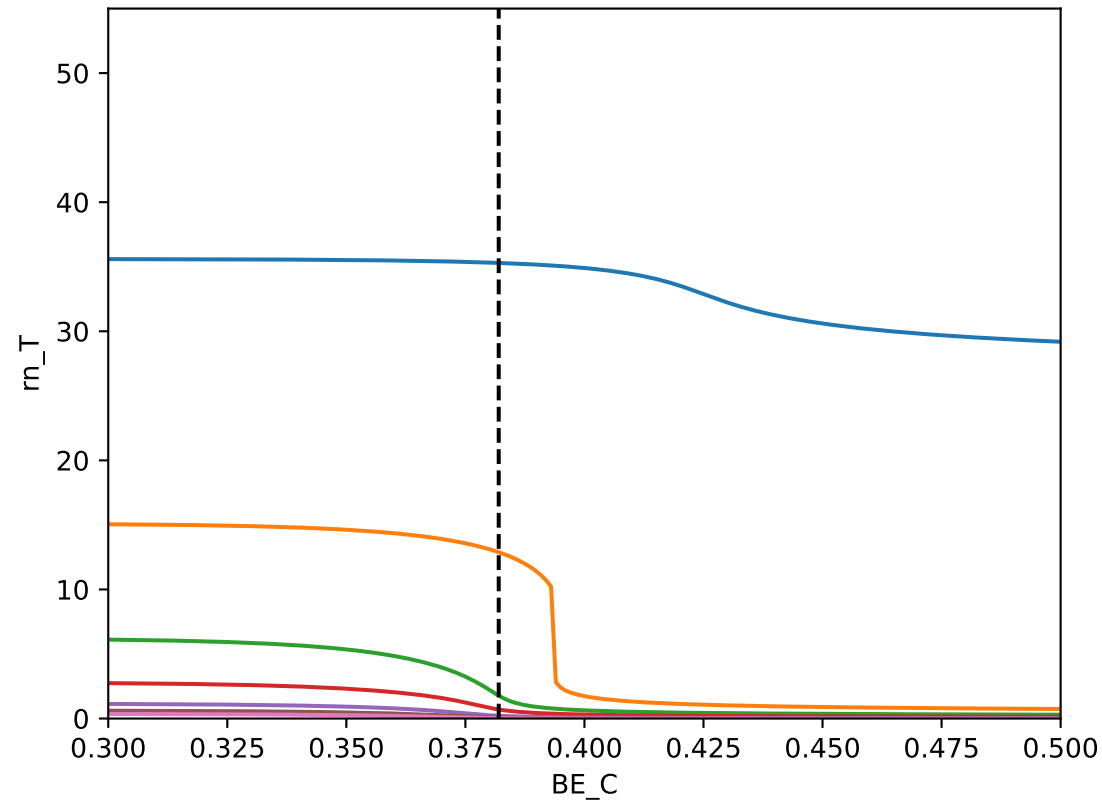

Supplement: S3 File — (ZIP) [file pcbi.1014035.s003.zip › 01-code/10-fig10/rnT_II.pdf]

# Connectivity II

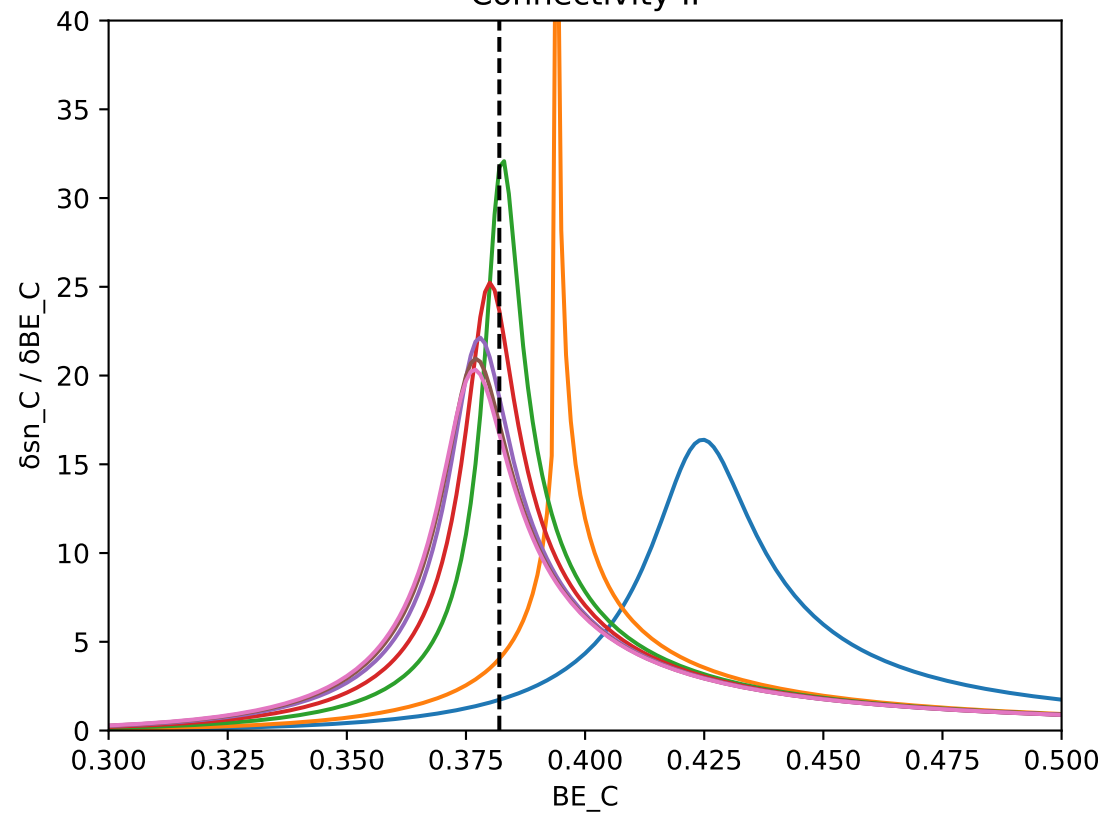

Supplement: S3 File — (ZIP) [file pcbi.1014035.s003.zip › 01-code/10-fig10/Sensi_snC_BEC_II.pdf]

# Connectivity II

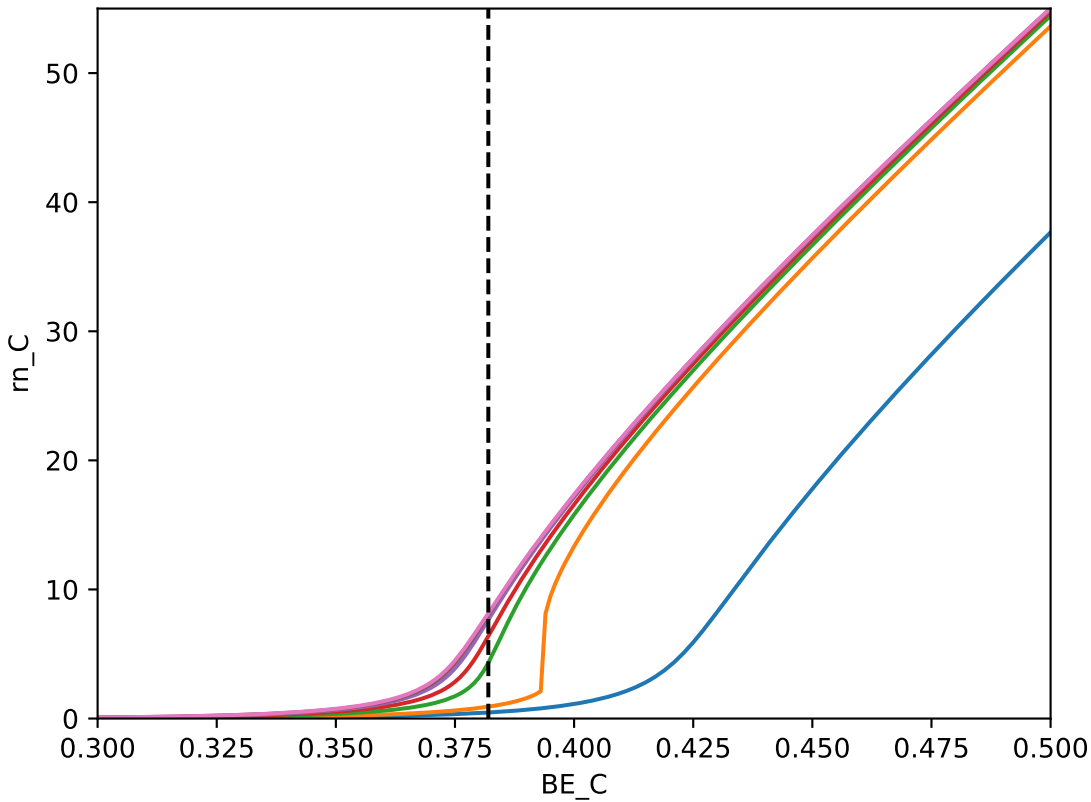

Supplement: S3 File — (ZIP) [file pcbi.1014035.s003.zip › 01-code/10-fig10/rnC_II.pdf]

## Connectivity II

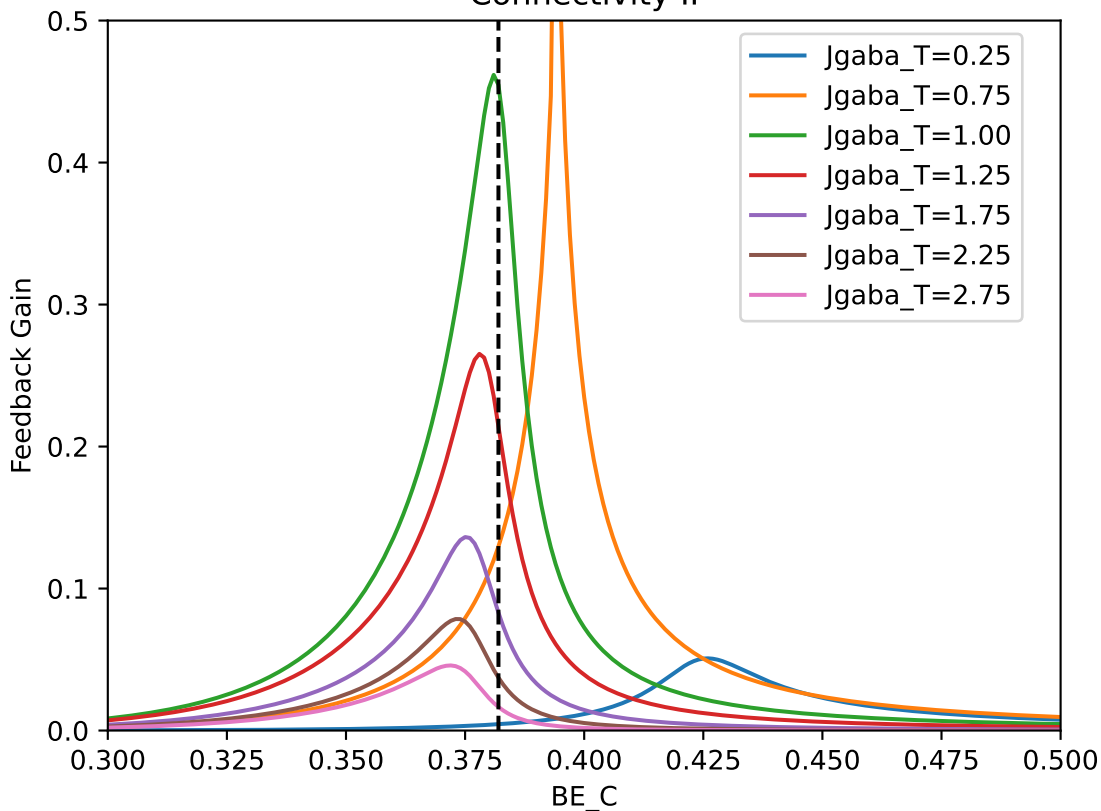

Supplement: S3 File — (ZIP) [file pcbi.1014035.s003.zip › 01-code/10-fig10/FeedbackGain_II.pdf]

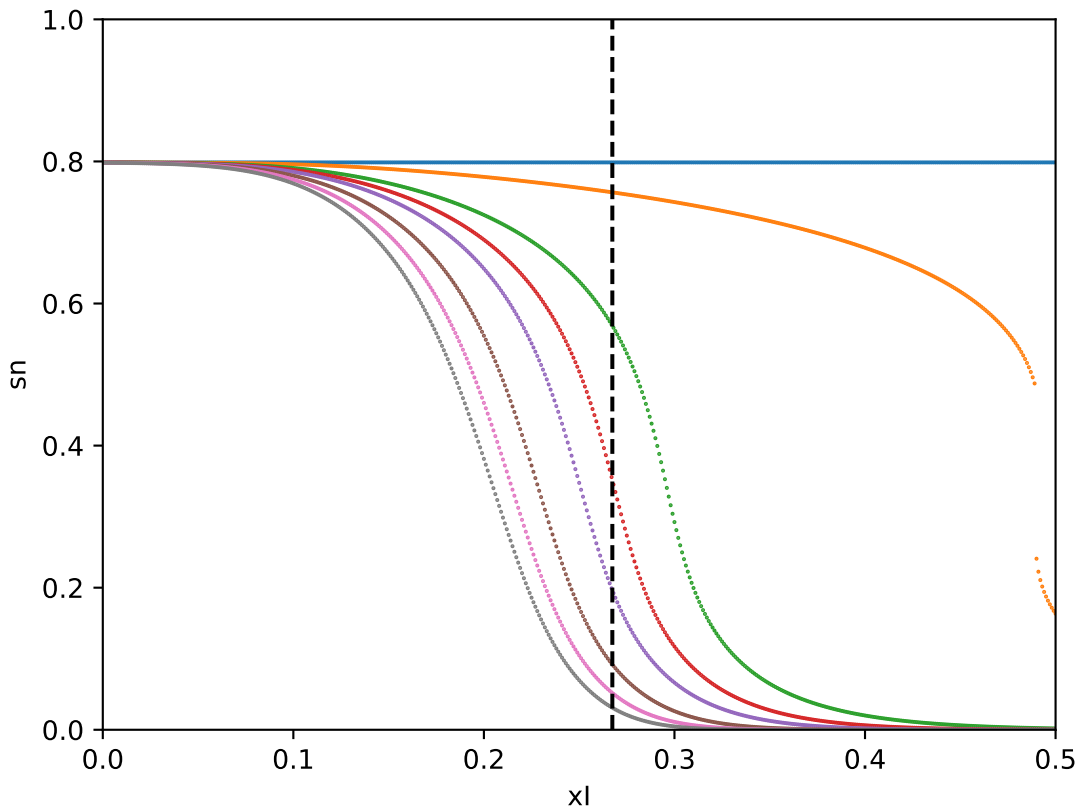

Supplement: S3 File — (ZIP) [file pcbi.1014035.s003.zip › 01-code/07-fig07/sn_xI.pdf]

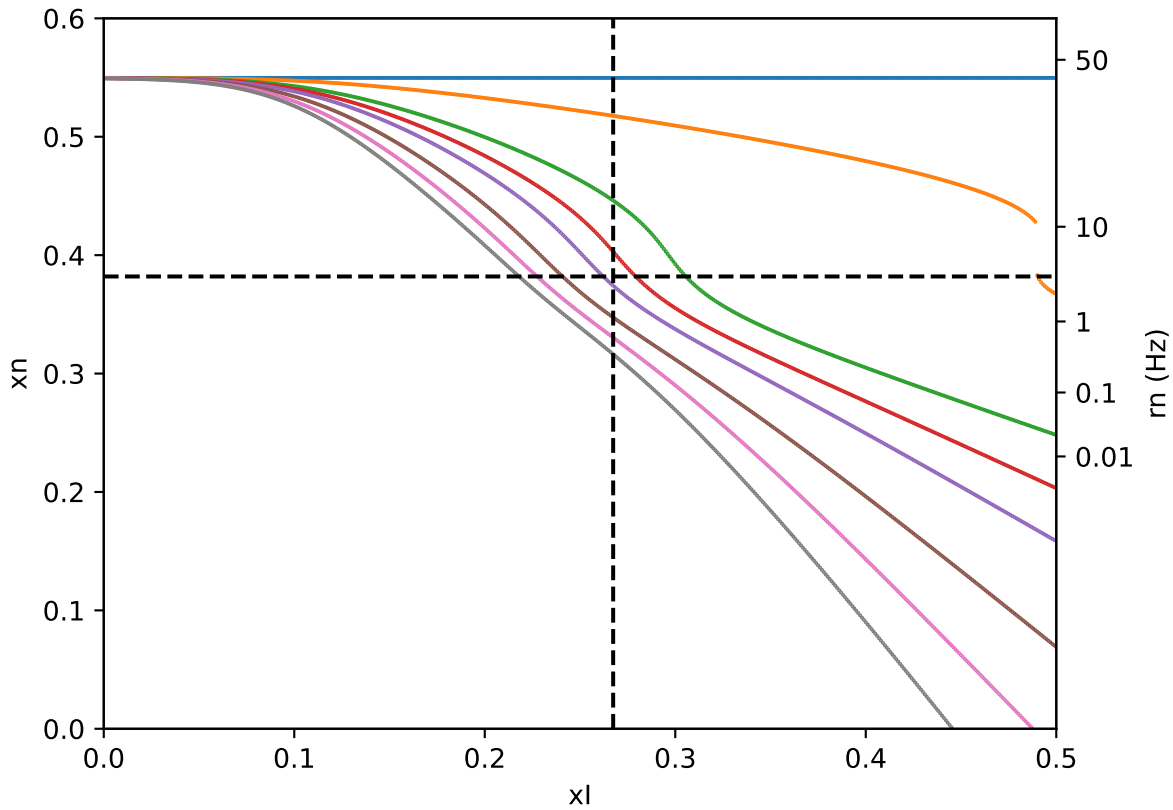

Supplement: S3 File — (ZIP) [file pcbi.1014035.s003.zip › 01-code/07-fig07/xn_xI.pdf]

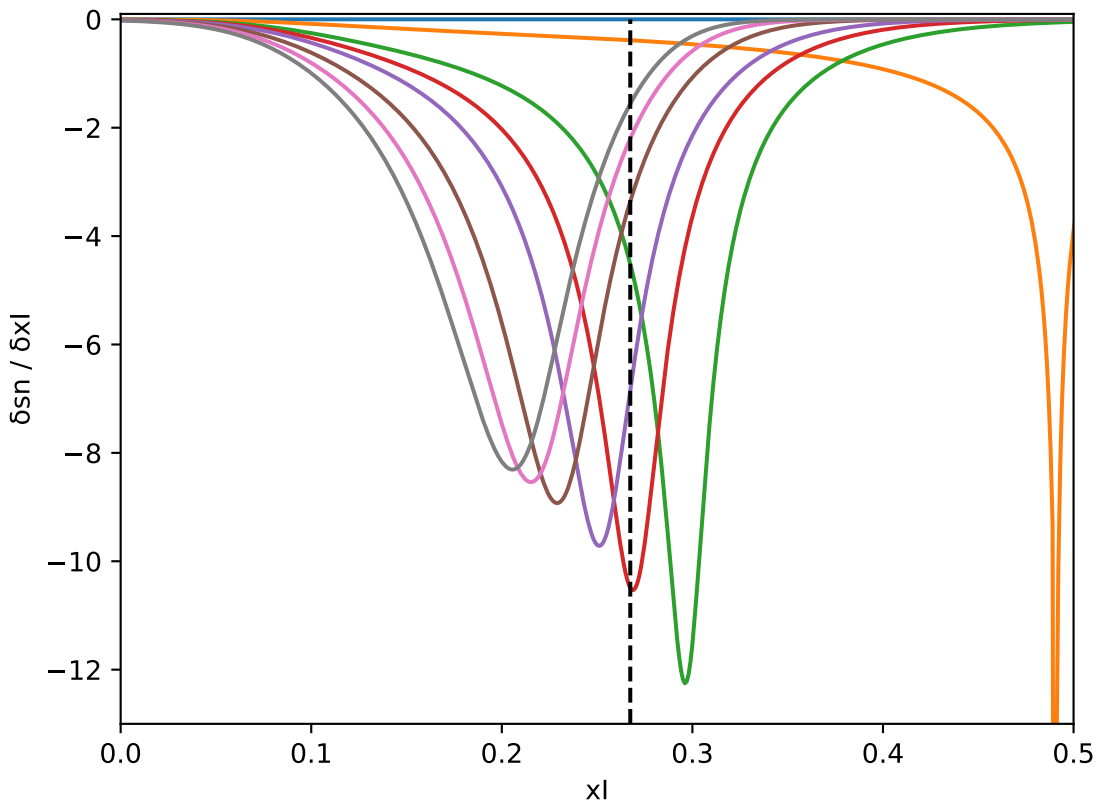

Supplement: S3 File — (ZIP) [file pcbi.1014035.s003.zip › 01-code/07-fig07/sensi_sn_xI.pdf]

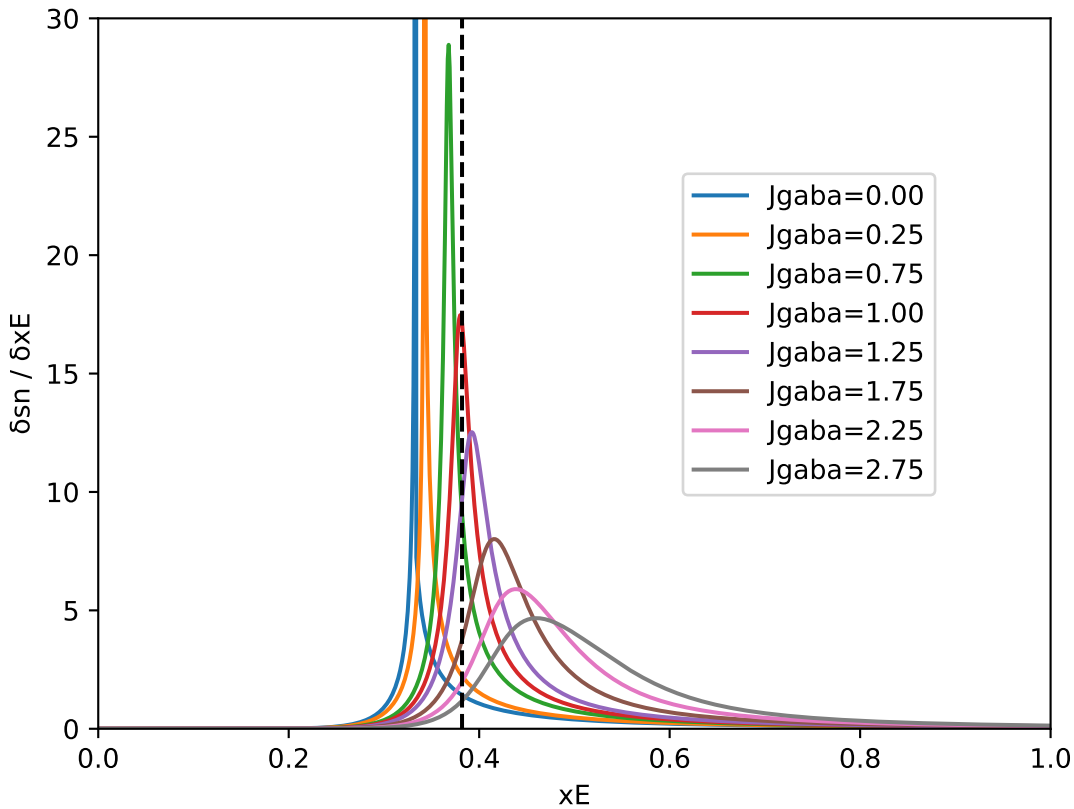

Supplement: S3 File — (ZIP) [file pcbi.1014035.s003.zip › 01-code/07-fig07/sensi_sn_xE.pdf]

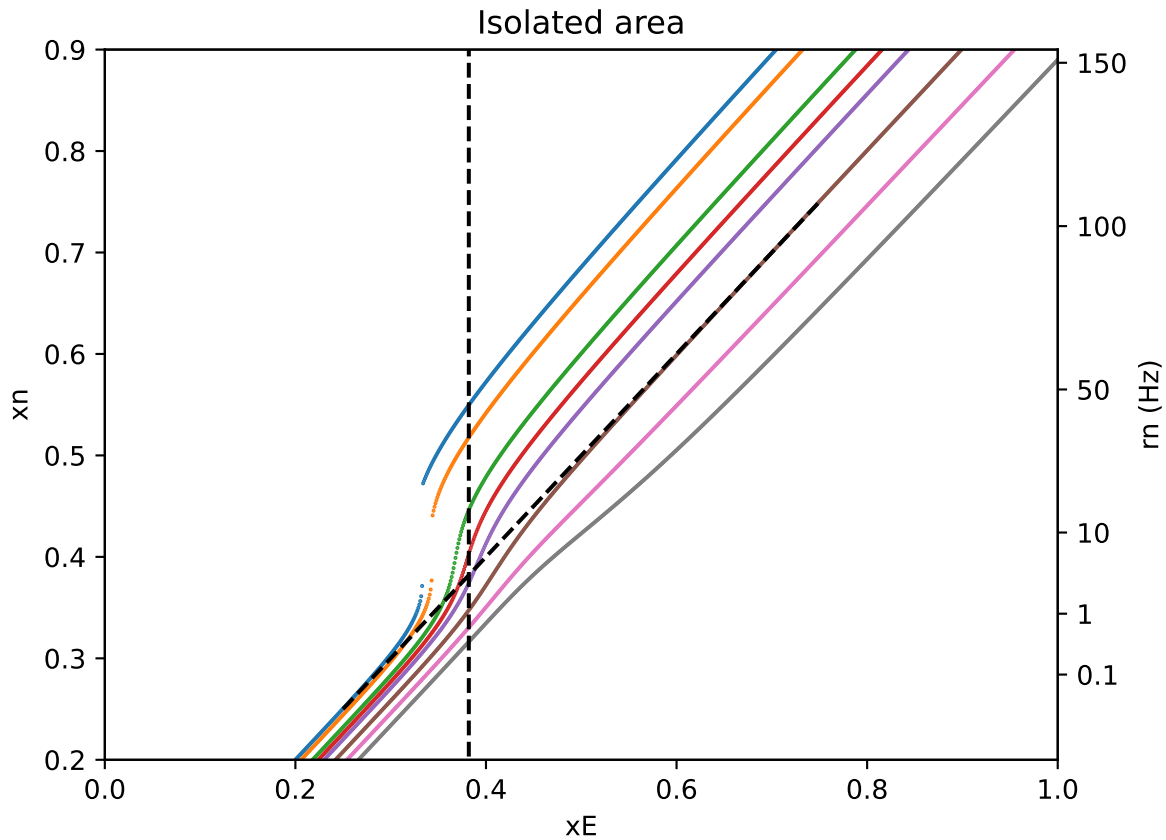

Supplement: S3 File — (ZIP) [file pcbi.1014035.s003.zip › 01-code/07-fig07/xn_xE.pdf]

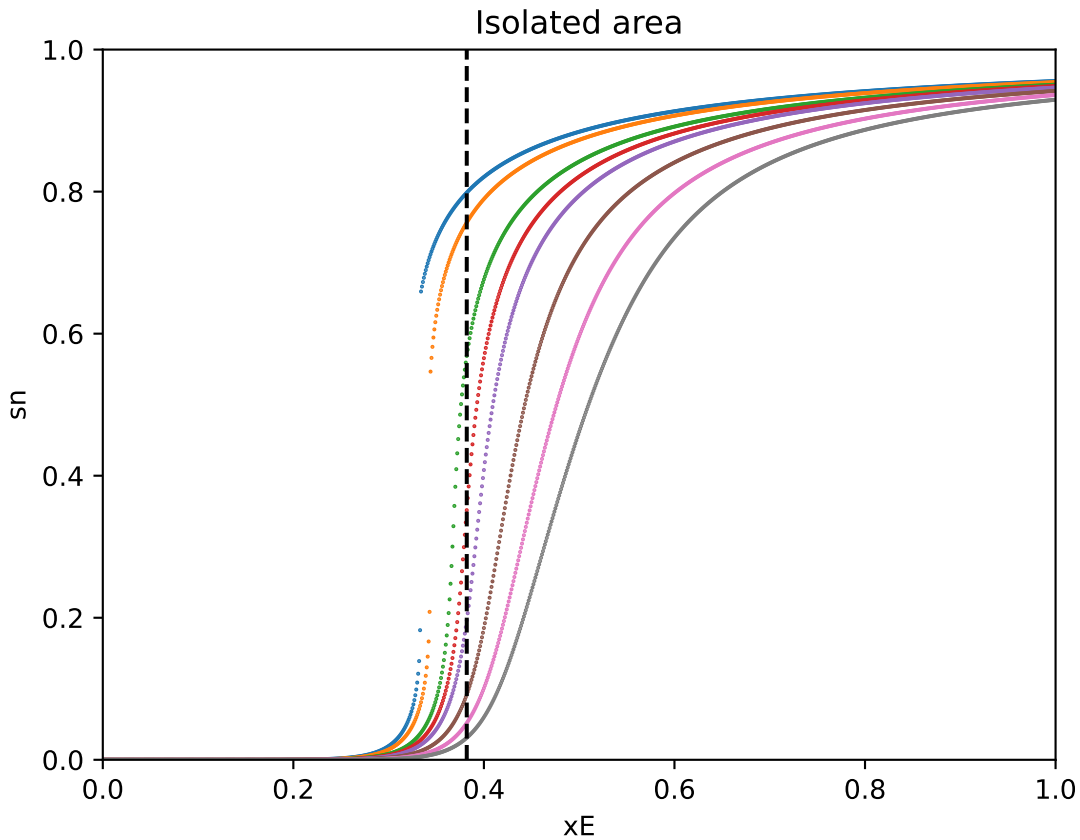

Supplement: S3 File — (ZIP) [file pcbi.1014035.s003.zip › 01-code/07-fig07/sn_xE.pdf]

Connectivity EE (fixed points calculations)

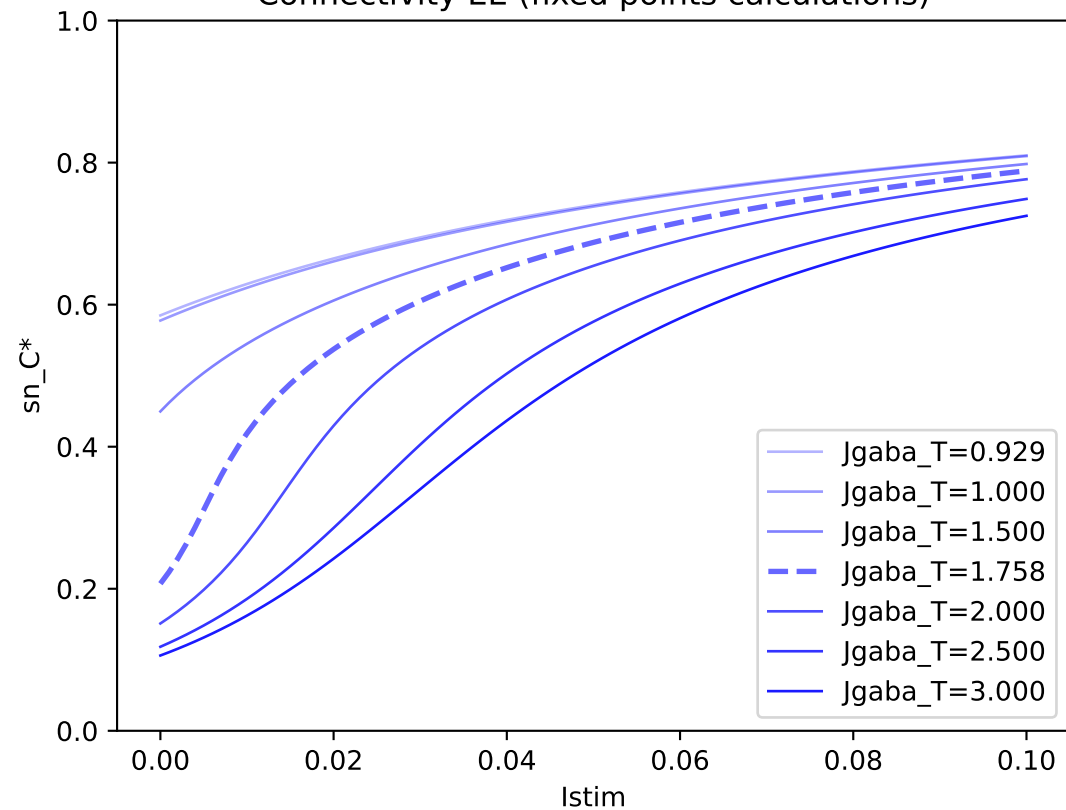

Supplement: S3 File — (ZIP) [file pcbi.1014035.s003.zip › 01-code/13-fig13/snC_Istim_EE.pdf]

Connectivity IE (fixed points calculations)

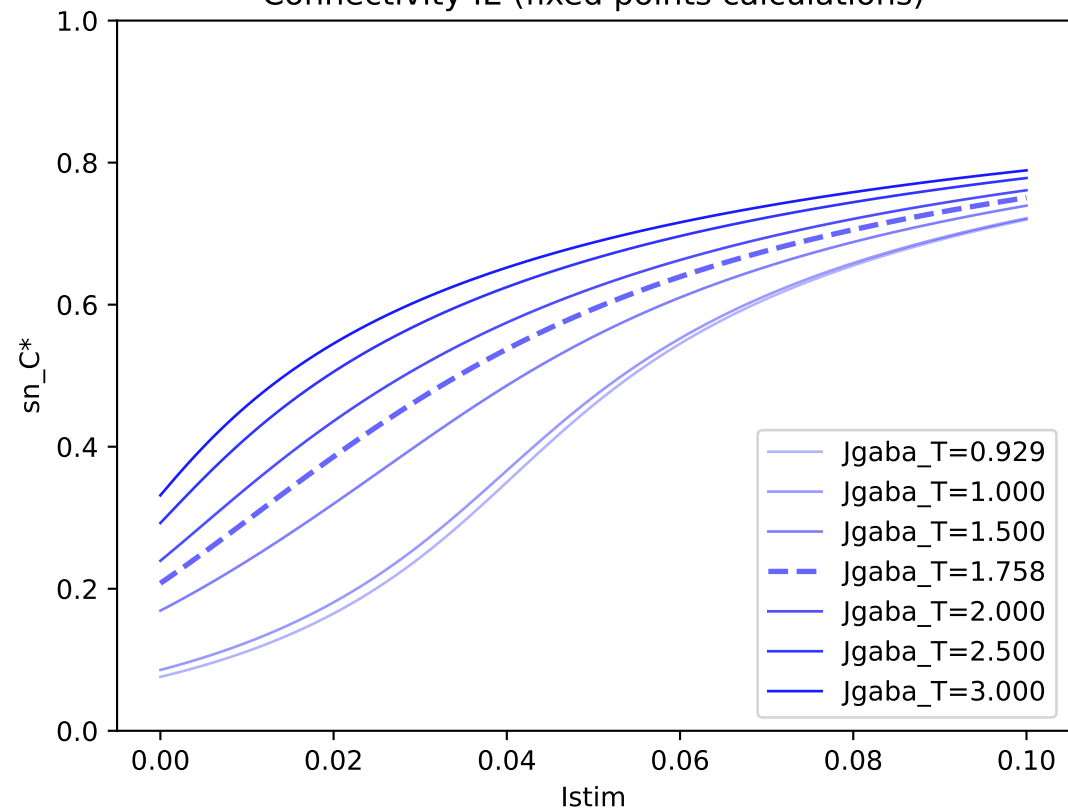

Supplement: S3 File — (ZIP) [file pcbi.1014035.s003.zip › 01-code/13-fig13/snC_Istim_IE.pdf]

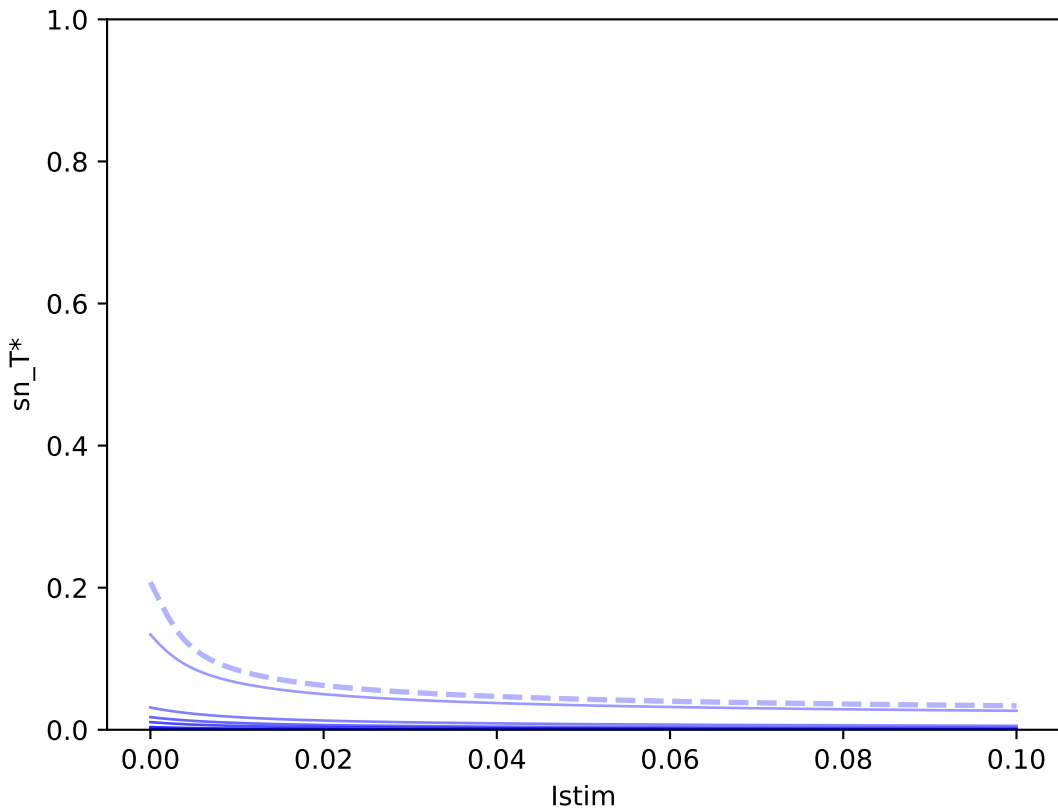

Supplement: S3 File — (ZIP) [file pcbi.1014035.s003.zip › 01-code/13-fig13/snT_Istim_II.pdf]

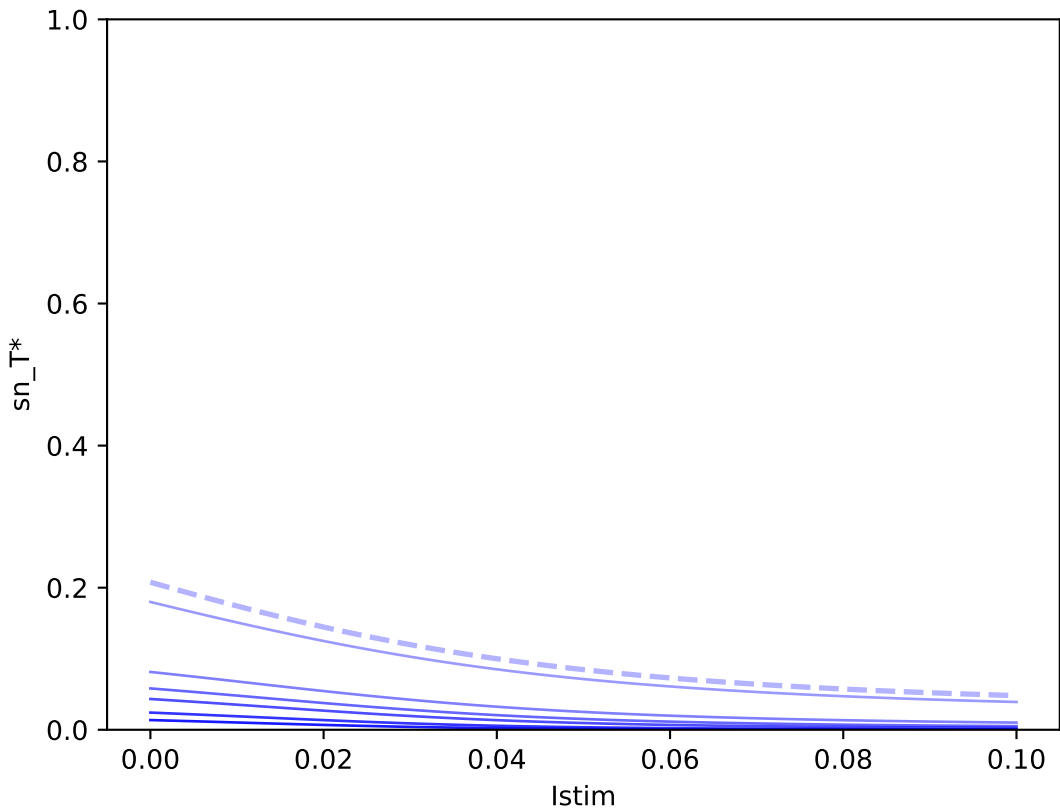

Supplement: S3 File — (ZIP) [file pcbi.1014035.s003.zip › 01-code/13-fig13/snT_Istim_EI.pdf]

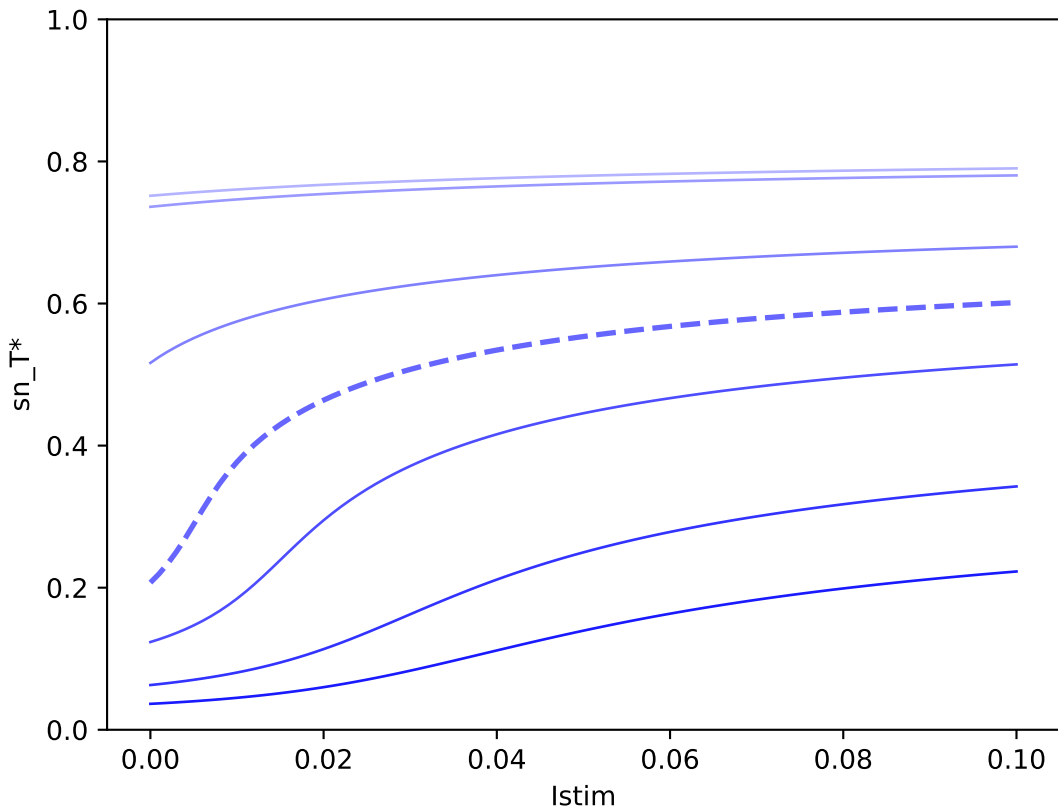

Supplement: S3 File — (ZIP) [file pcbi.1014035.s003.zip › 01-code/13-fig13/snT_Istim_EE.pdf]

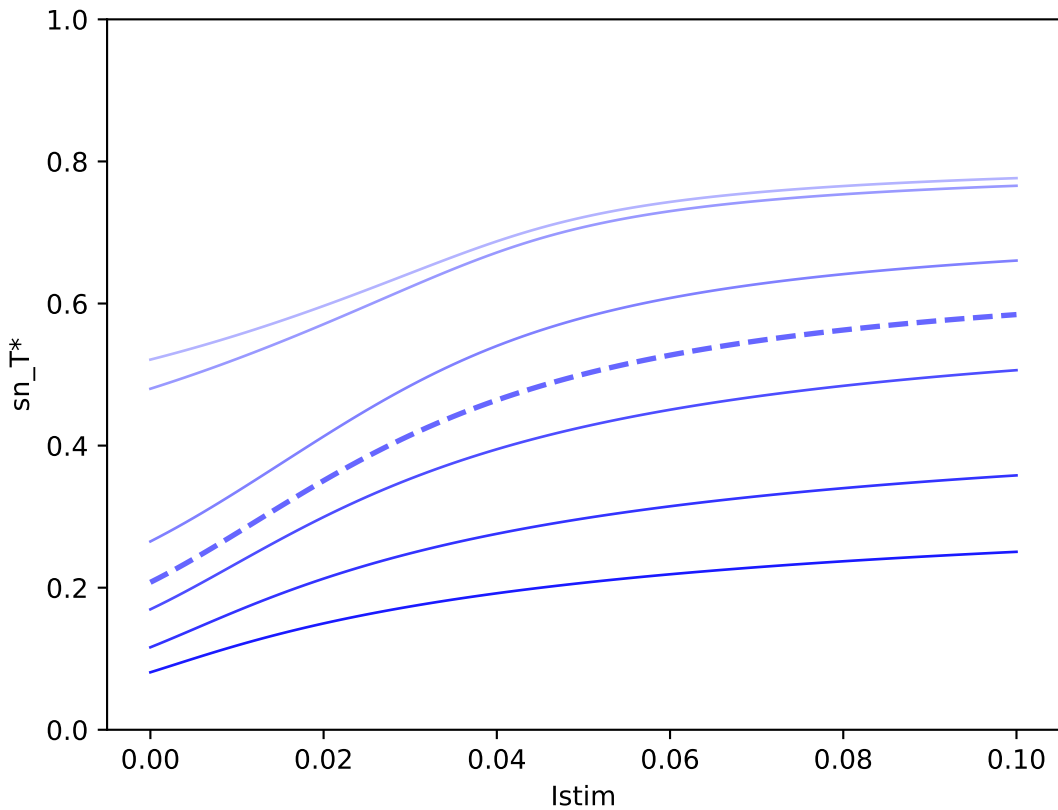

Supplement: S3 File — (ZIP) [file pcbi.1014035.s003.zip › 01-code/13-fig13/snT_Istim_IE.pdf]

## Connectivity II (fixed points calculations)

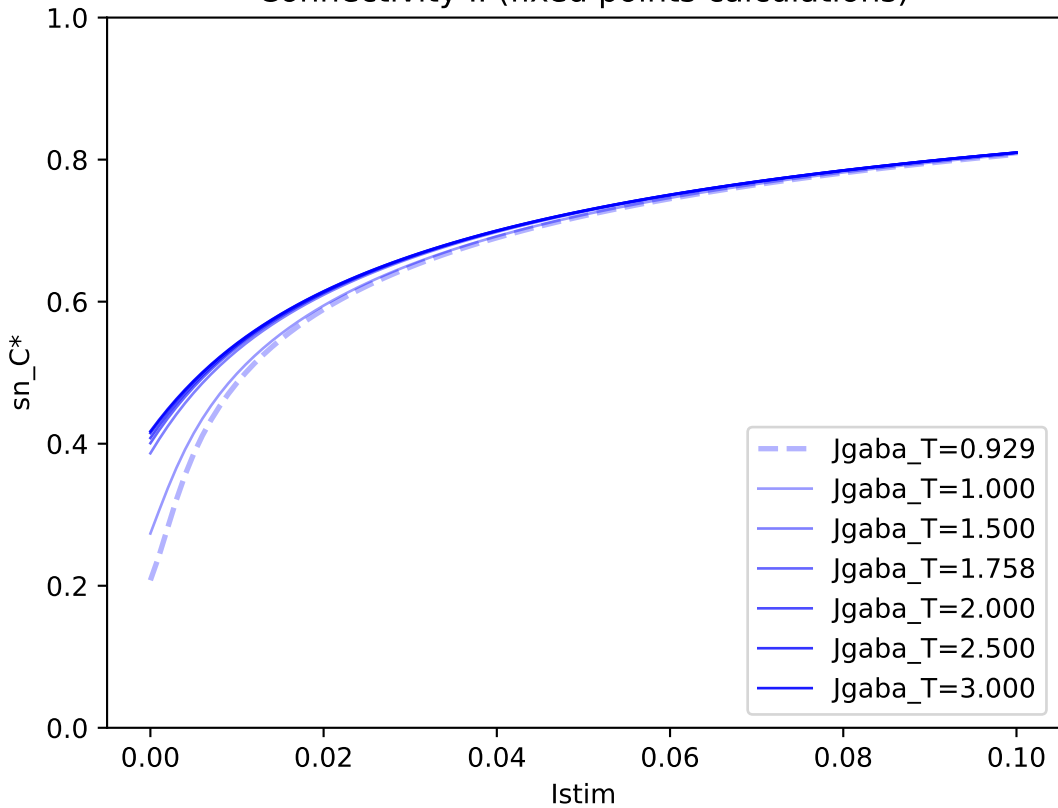

Supplement: S3 File — (ZIP) [file pcbi.1014035.s003.zip › 01-code/13-fig13/snC_Istim_II.pdf]

Connectivity EI (fixed points calculations)

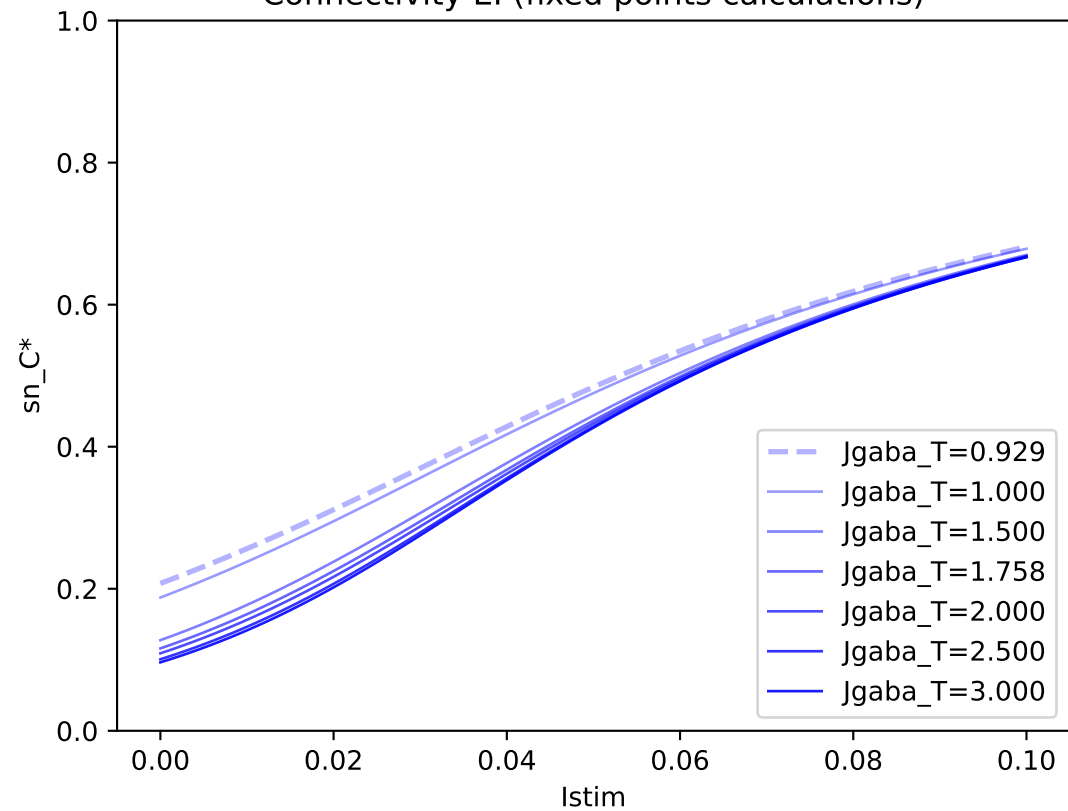

Supplement: S3 File — (ZIP) [file pcbi.1014035.s003.zip › 01-code/13-fig13/snC_Istim_EI.pdf]

# Isolated excitatory pool

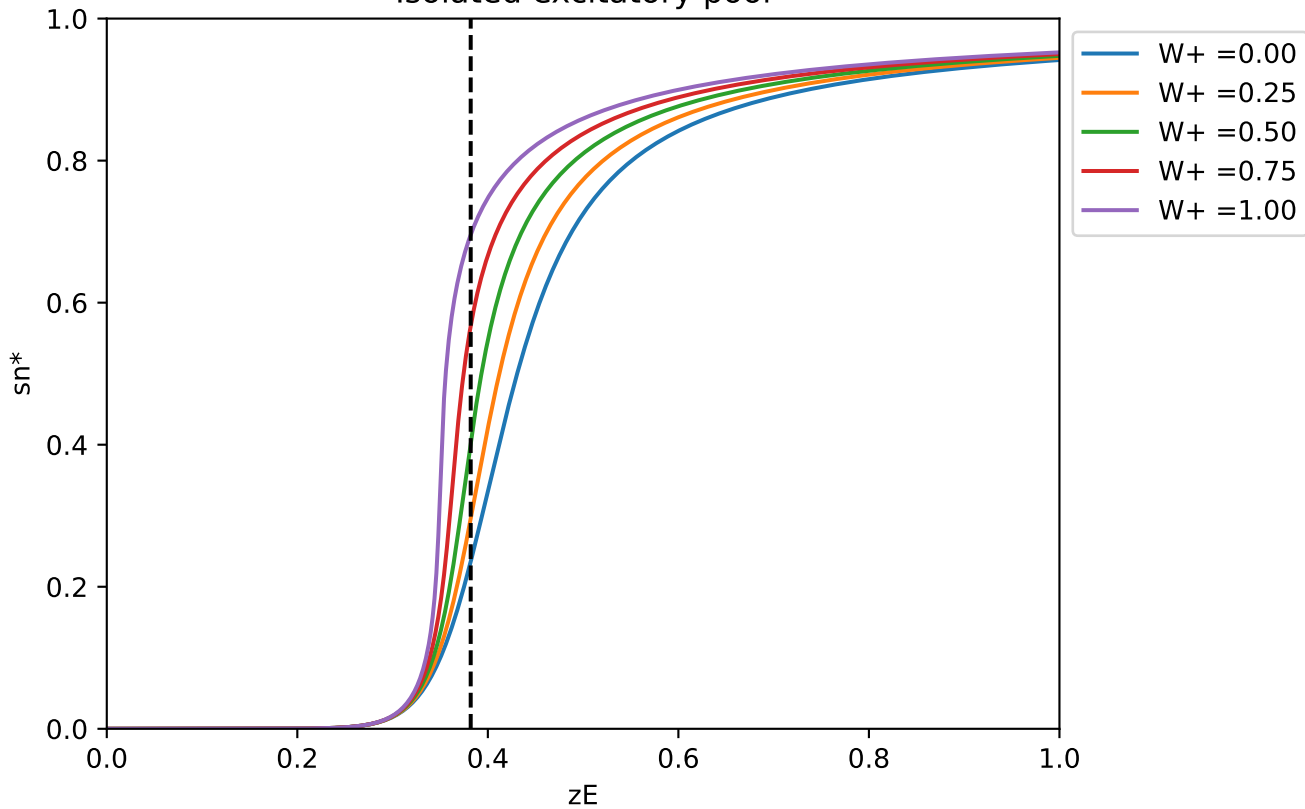

Supplement: S3 File — (ZIP) [file pcbi.1014035.s003.zip › 01-code/04-fig04/sn_zE.pdf]

Isolated excitatory pool

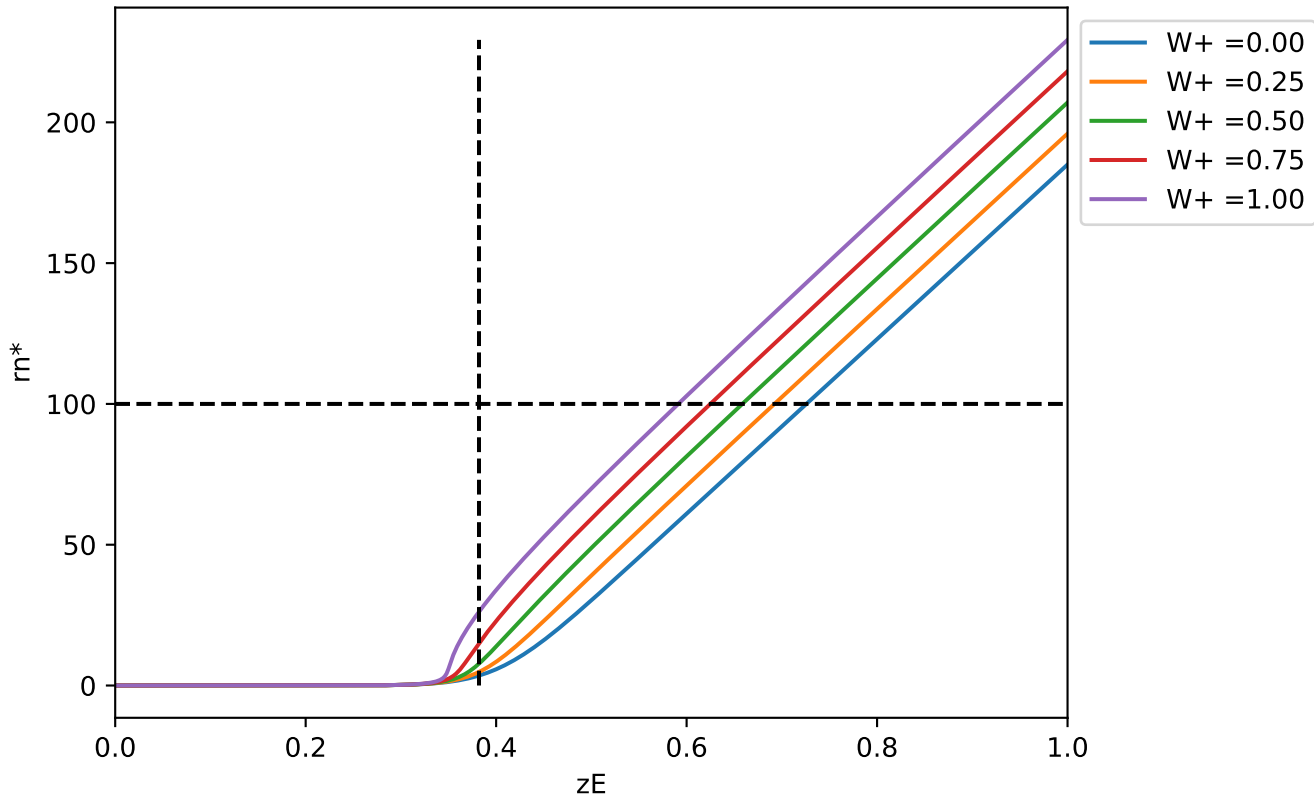

Supplement: S3 File — (ZIP) [file pcbi.1014035.s003.zip › 01-code/04-fig04/rn_zE.pdf]

# Isolated excitatory pool

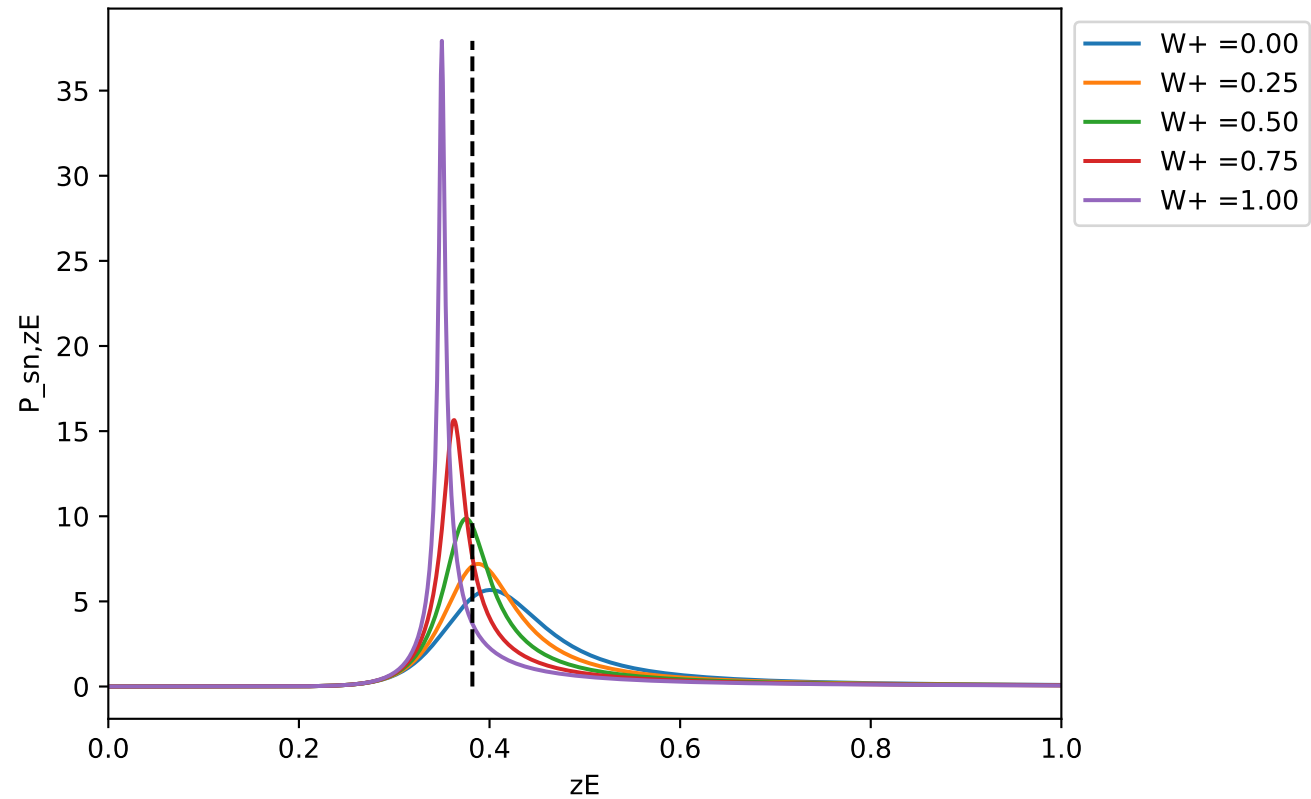

Supplement: S3 File — (ZIP) [file pcbi.1014035.s003.zip › 01-code/04-fig04/Close_Loop_Sensitivity.pdf]

Isolated excitatory pool

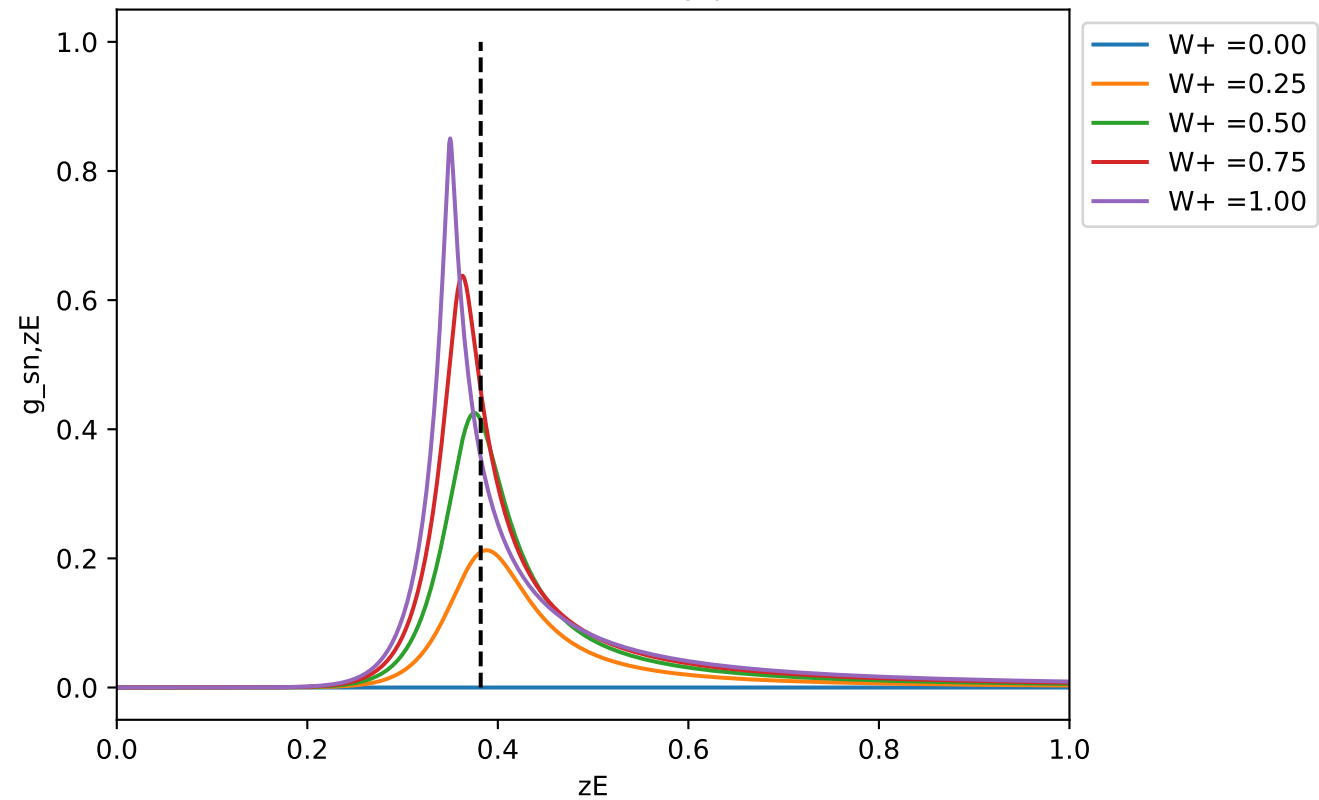

Supplement: S3 File — (ZIP) [file pcbi.1014035.s003.zip › 01-code/04-fig04/Feedback_Gain.pdf]

one isolated area system

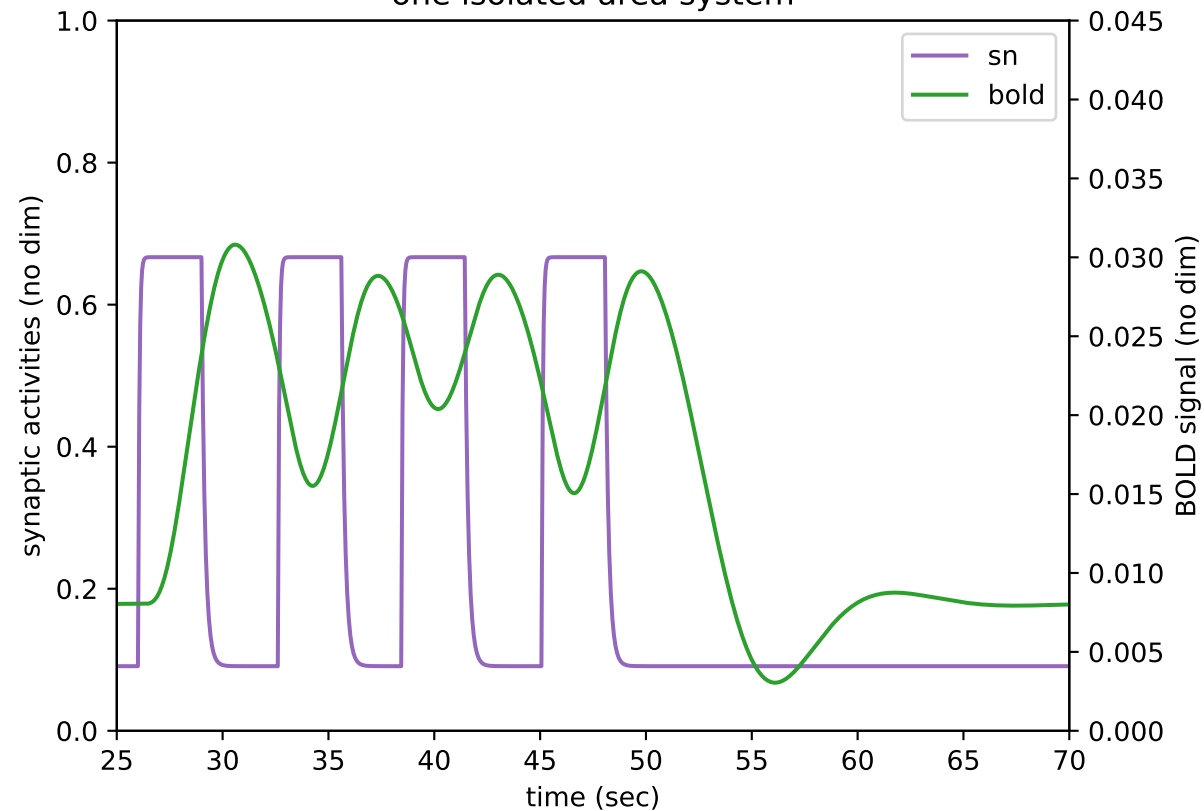

Supplement: S3 File — (ZIP) [file pcbi.1014035.s003.zip › 01-code/02-fig02/Sn_Bold.pdf]

# Connectivity IE

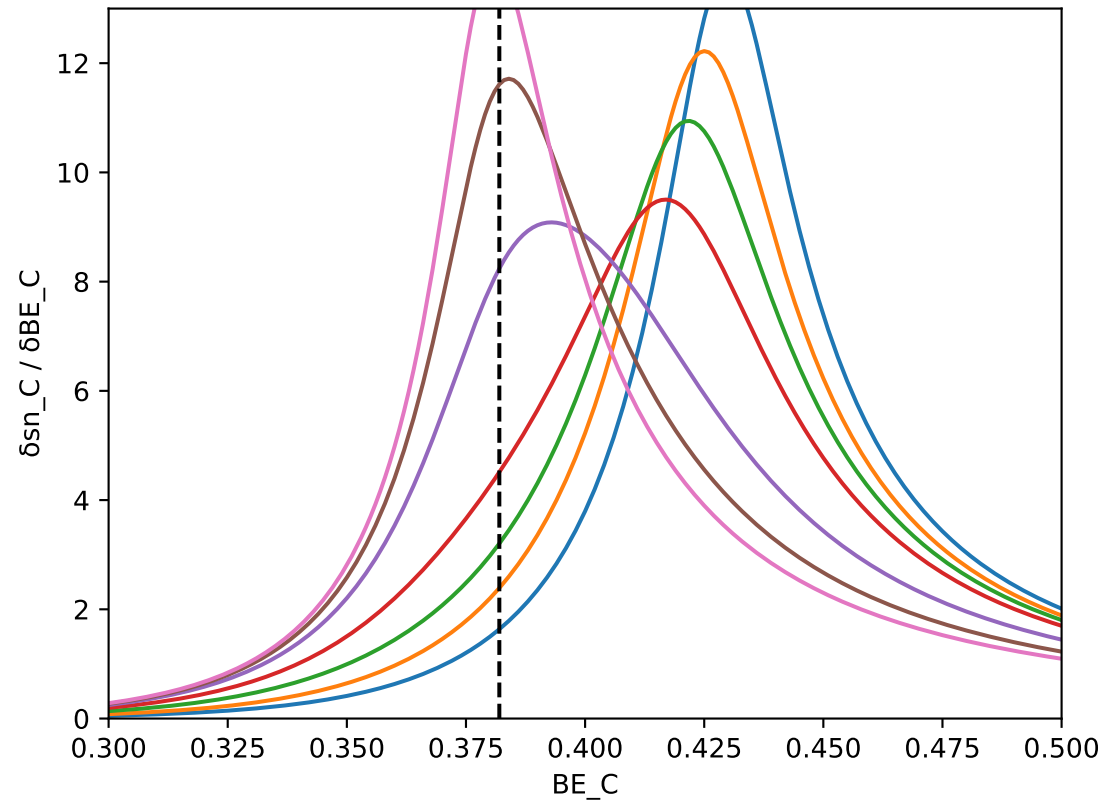

Supplement: S3 File — (ZIP) [file pcbi.1014035.s003.zip › 01-code/09-fig09/Sensi_snC_BEC_IE.pdf]

Connectivity IE

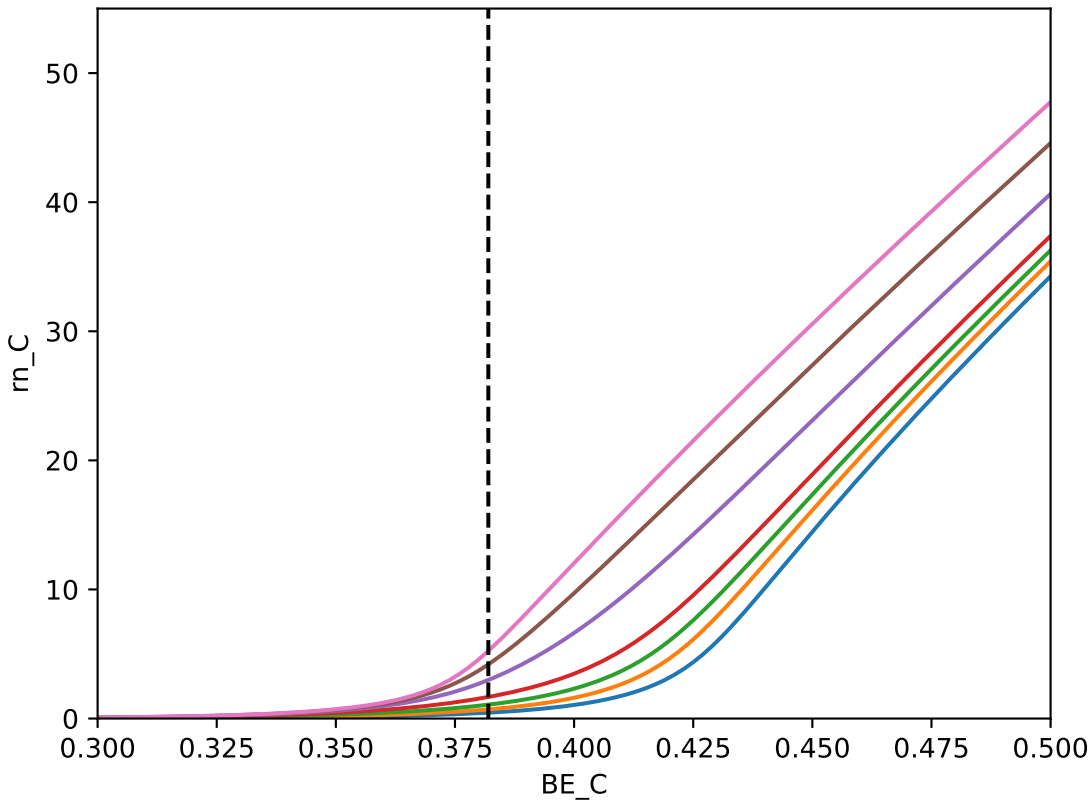

Supplement: S3 File — (ZIP) [file pcbi.1014035.s003.zip › 01-code/09-fig09/rnC_IE.pdf]

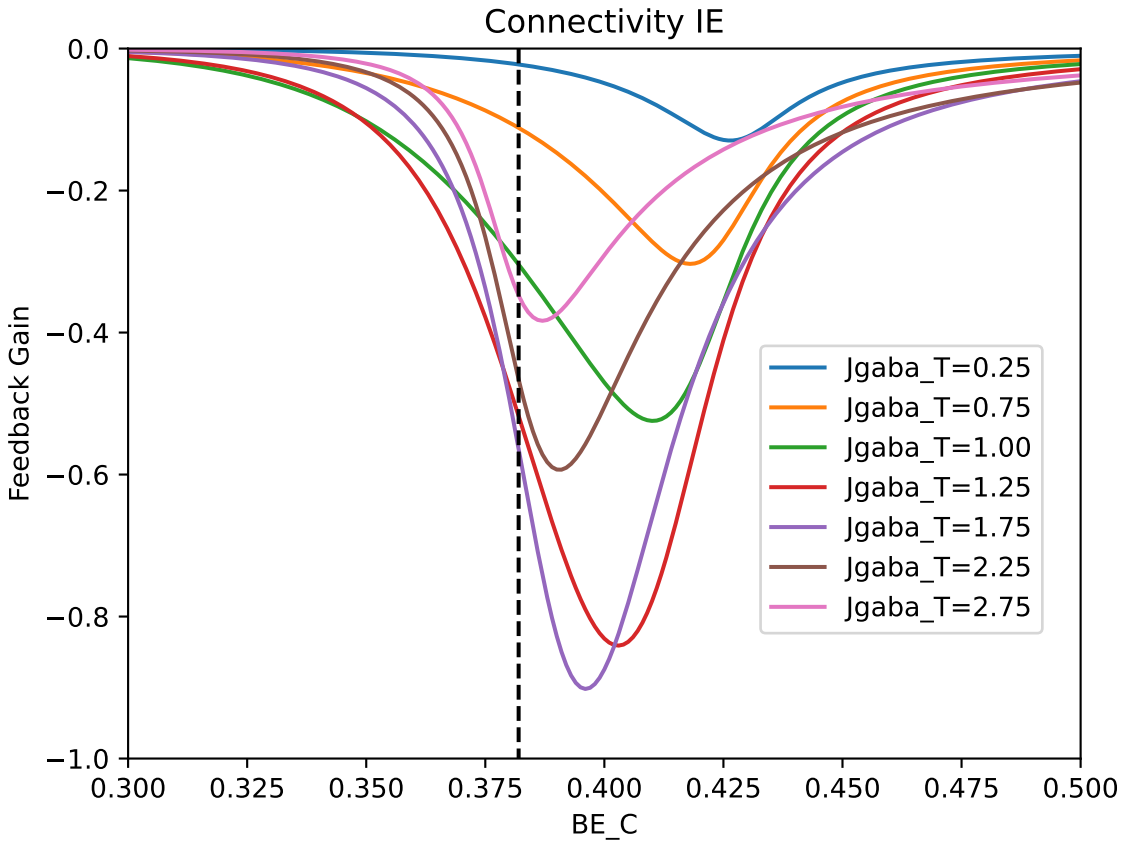

Supplement: S3 File — (ZIP) [file pcbi.1014035.s003.zip › 01-code/09-fig09/FeedbackGain_IE.pdf]

Connectivity IE

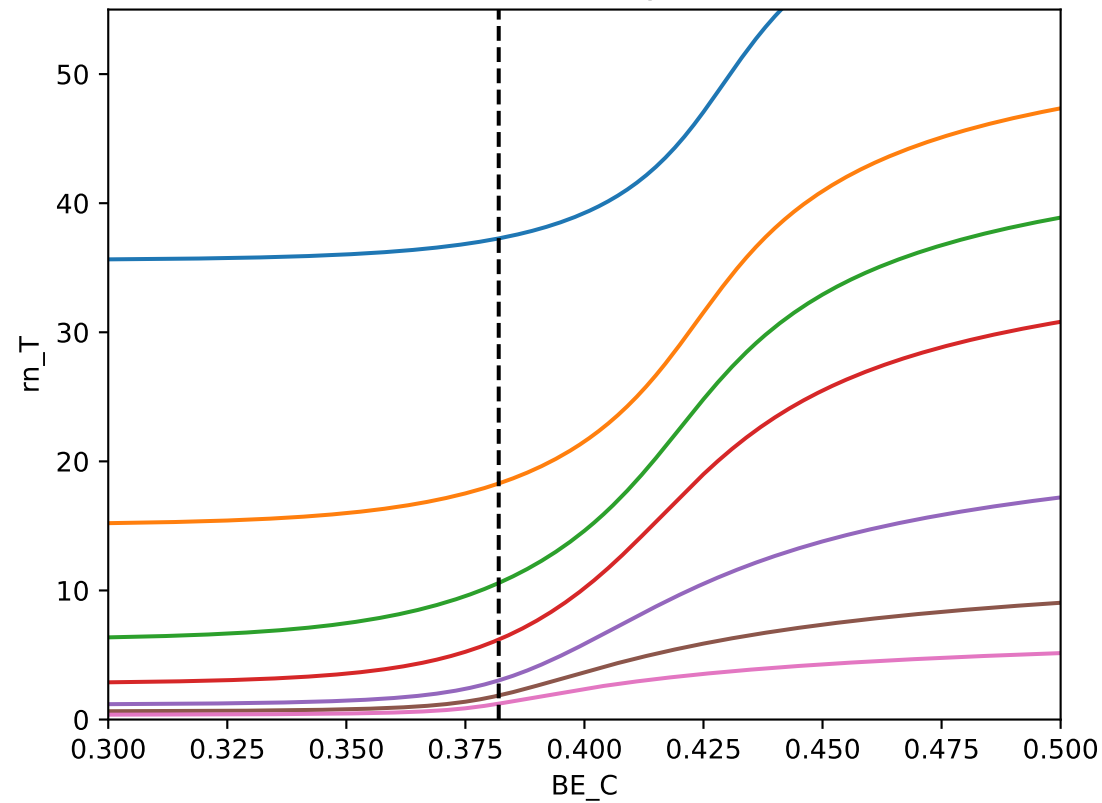

Supplement: S3 File — (ZIP) [file pcbi.1014035.s003.zip › 01-code/09-fig09/rnT_IE.pdf]

# Connectivity IE

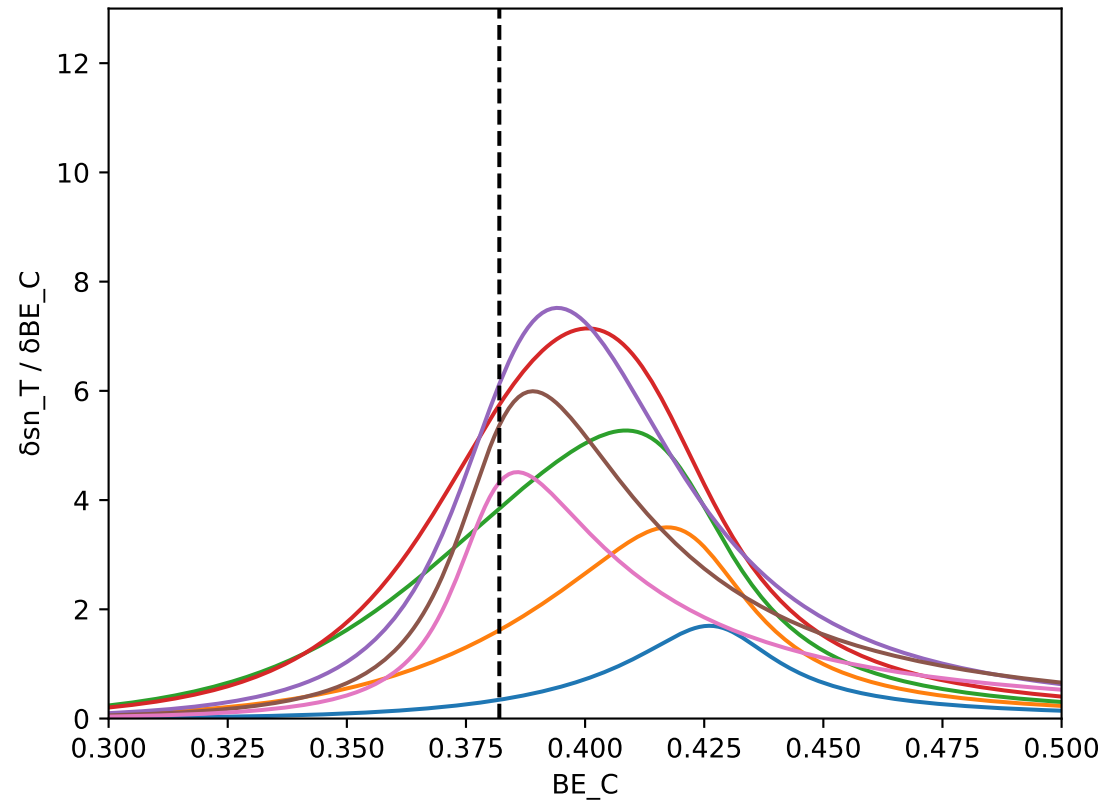

Supplement: S3 File — (ZIP) [file pcbi.1014035.s003.zip › 01-code/09-fig09/Sensi_snT_BEC_IE.pdf]

# 2 cerebral areas system

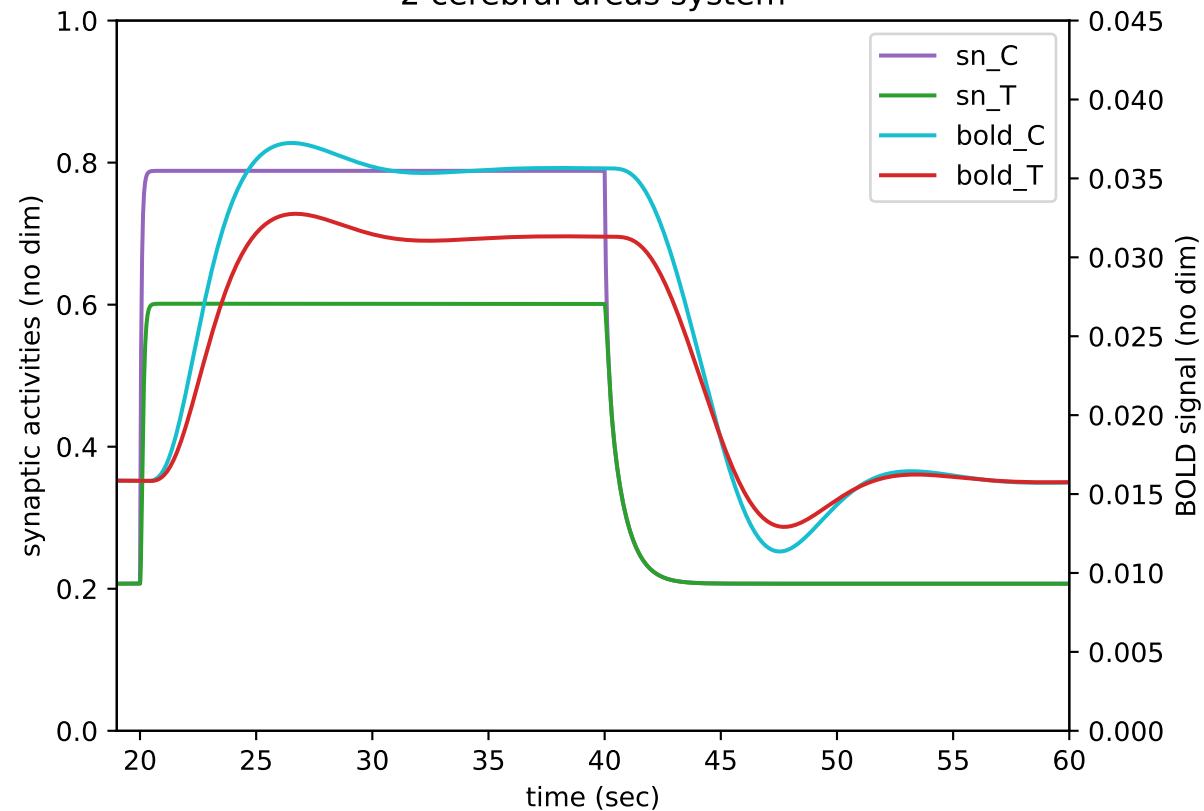

Supplement: S3 File — (ZIP) [file pcbi.1014035.s003.zip › 01-code/12-fig12/Sn_Bold_1Stim_EE.pdf]

# 2 cerebral areas system

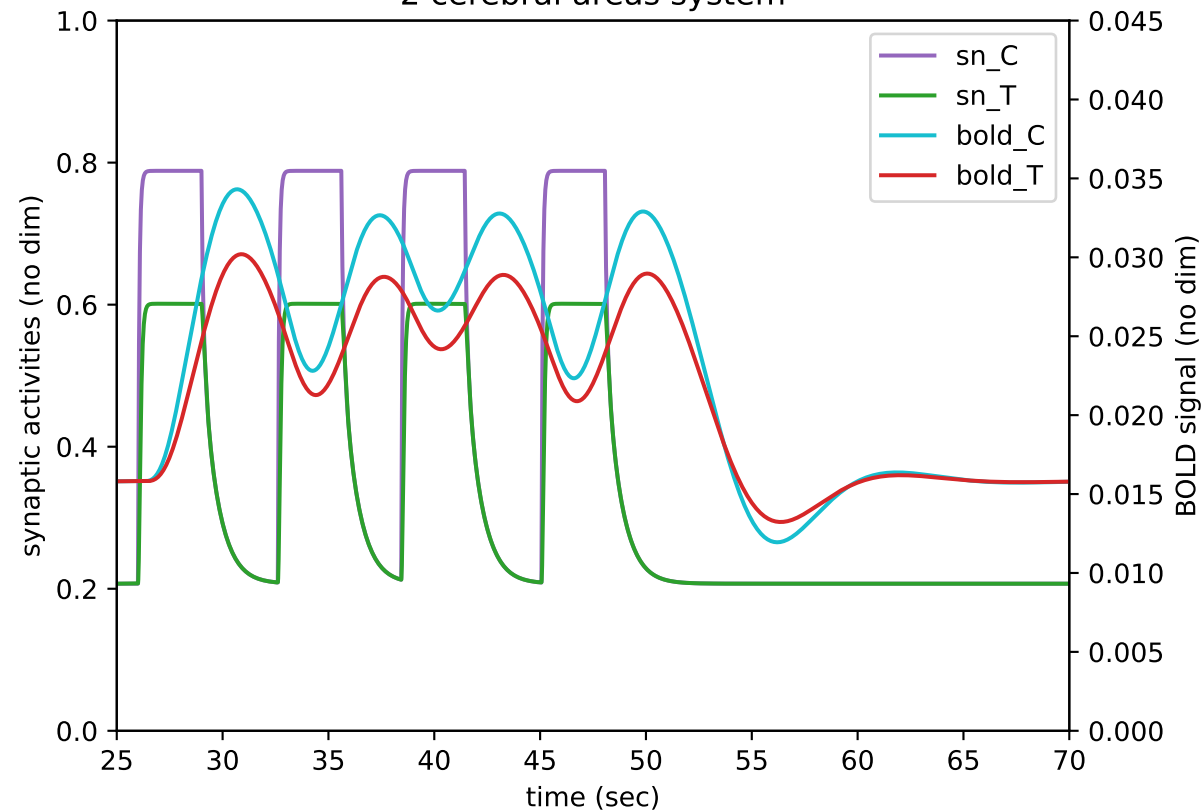

Supplement: S3 File — (ZIP) [file pcbi.1014035.s003.zip › 01-code/12-fig12/Sn_Bold_4Stim_EE.pdf]

# 2 cerebral areas system

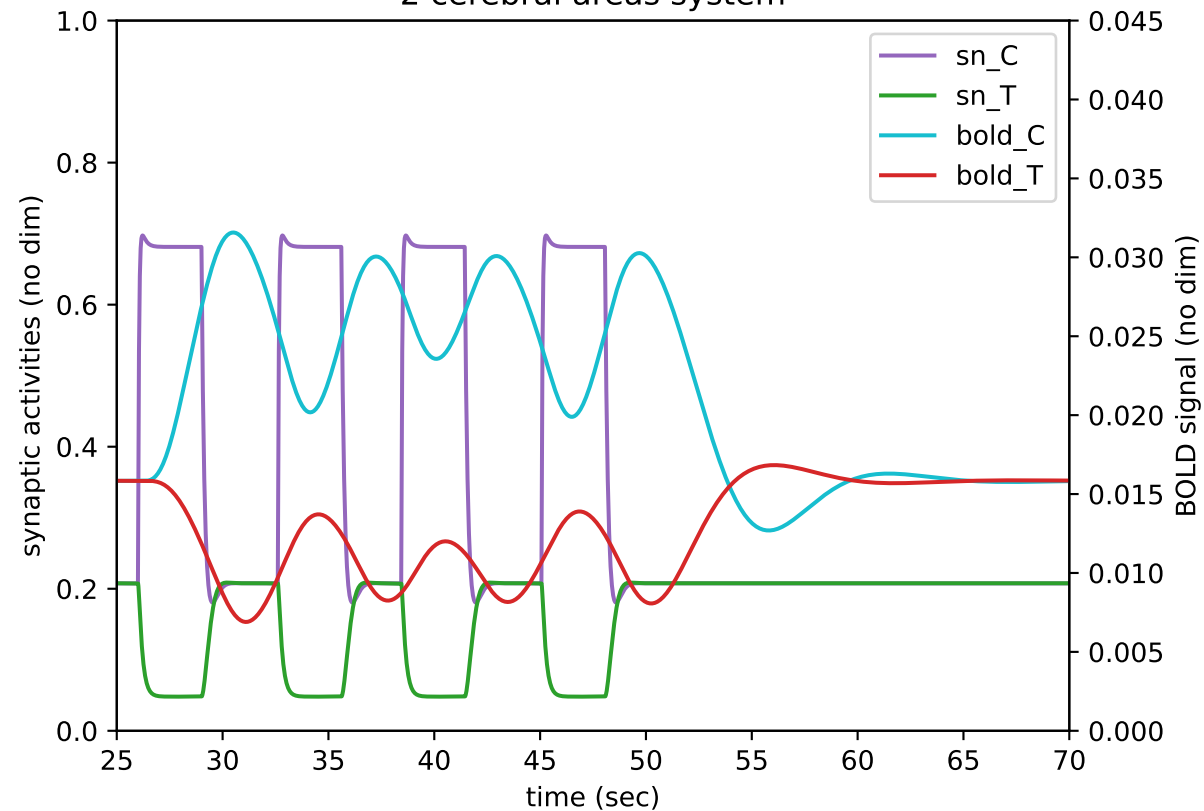

Supplement: S3 File — (ZIP) [file pcbi.1014035.s003.zip › 01-code/12-fig12/Sn_Bold_4Stim_EI.pdf]

2 cerebral areas system

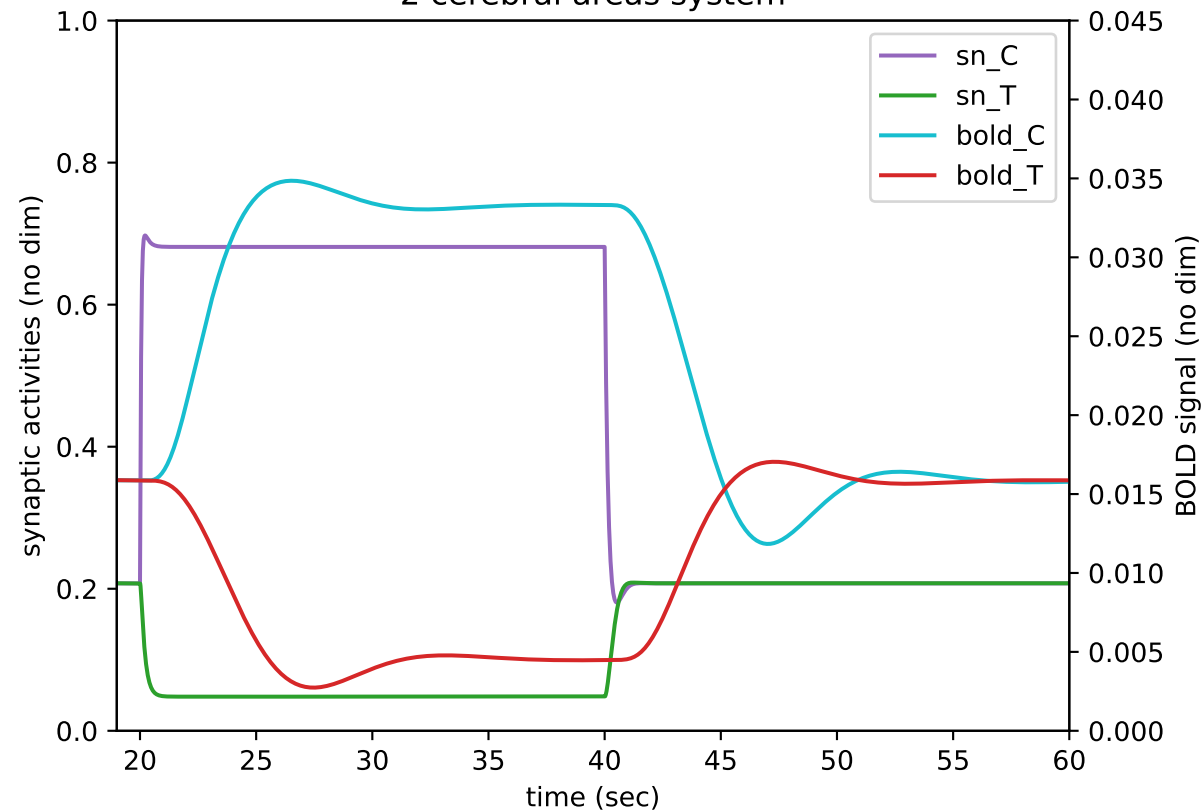

Supplement: S3 File — (ZIP) [file pcbi.1014035.s003.zip › 01-code/12-fig12/Sn_Bold_1Stim_EI.pdf]

Isolated inhibitory pool

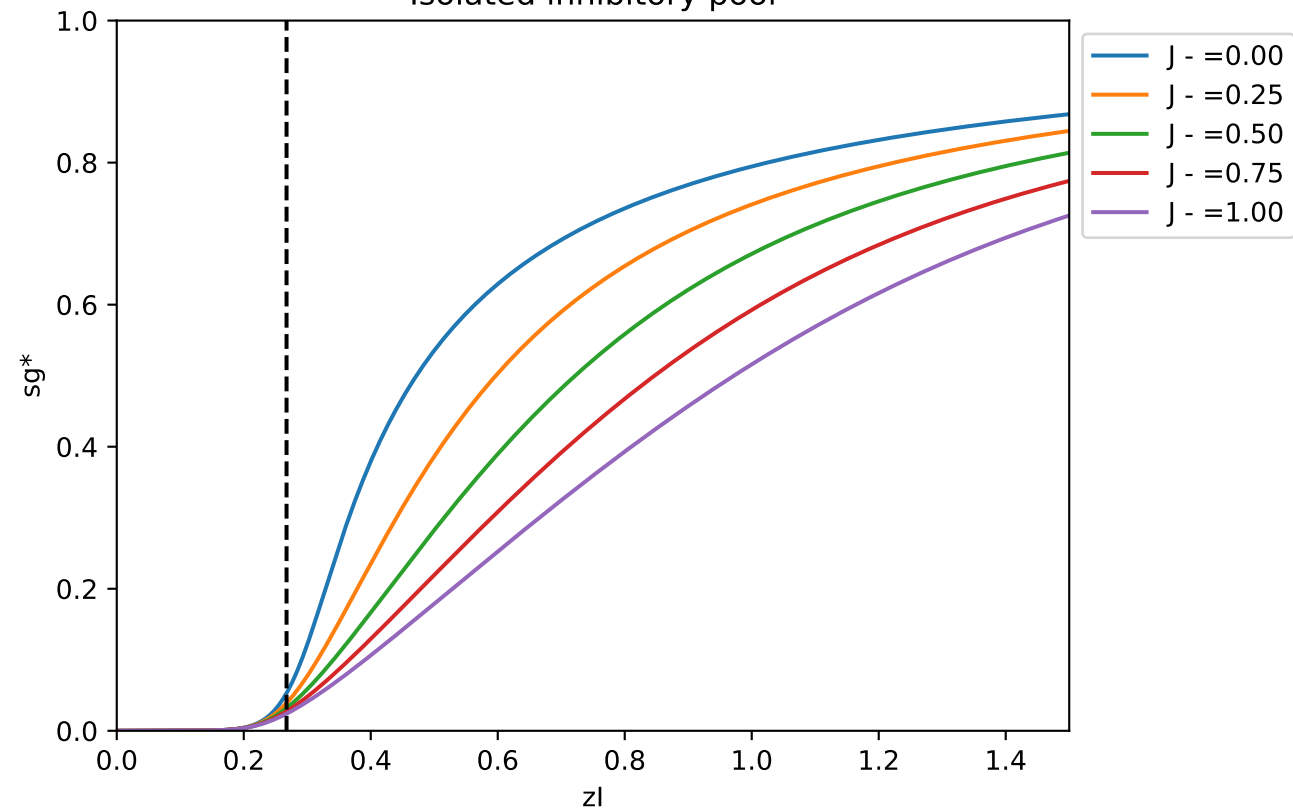

Supplement: S3 File — (ZIP) [file pcbi.1014035.s003.zip › 01-code/05-fig05/sg_zI.pdf]

Isolated inhibitory pool

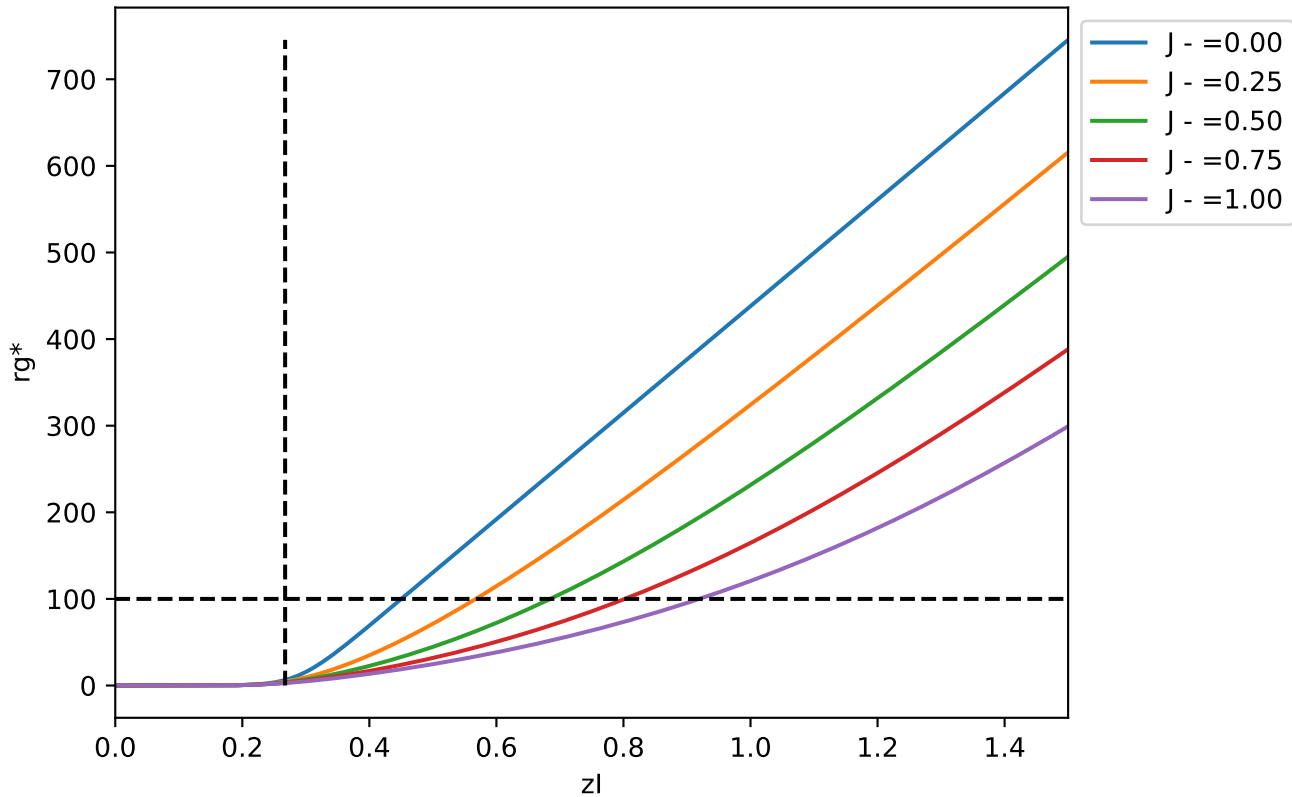

Supplement: S3 File — (ZIP) [file pcbi.1014035.s003.zip › 01-code/05-fig05/rg_zI.pdf]

Isolated inhibitory pool

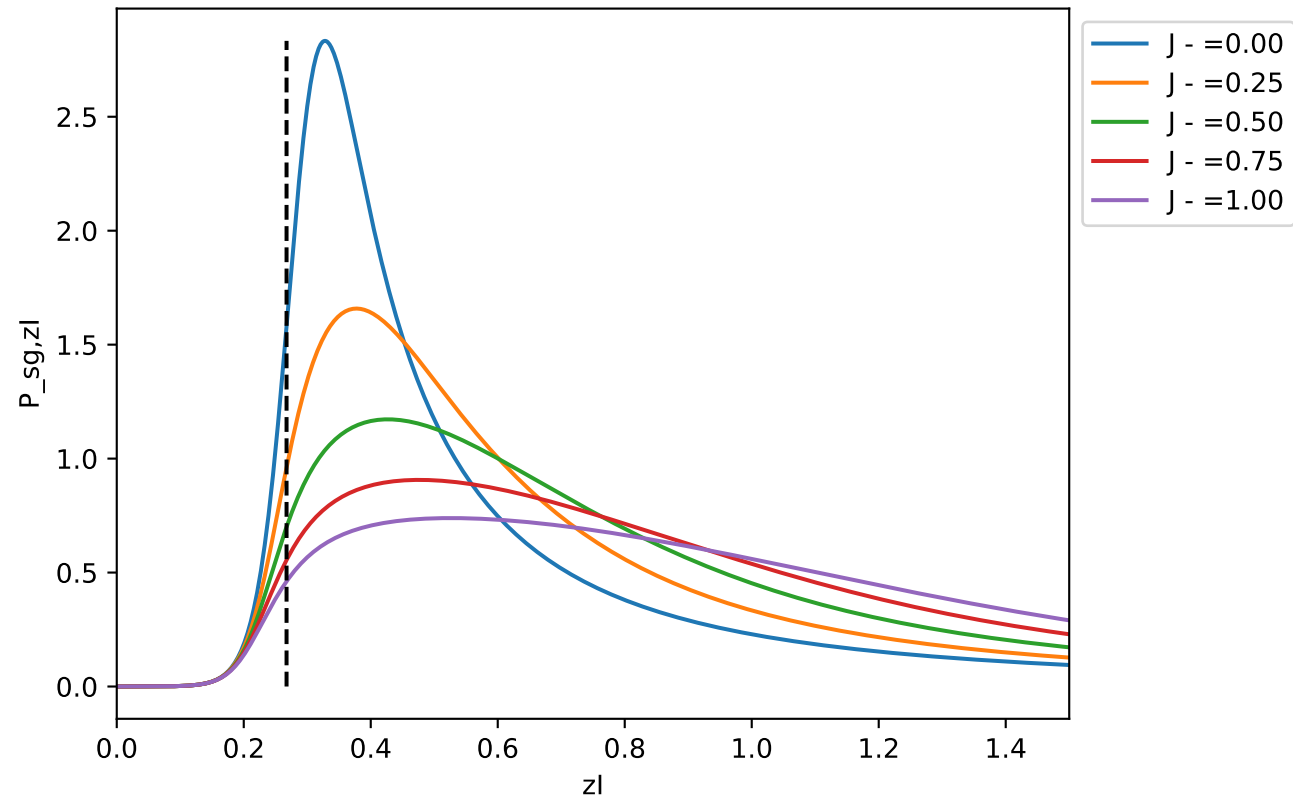

Supplement: S3 File — (ZIP) [file pcbi.1014035.s003.zip › 01-code/05-fig05/Close_Loop_Sensitivity.pdf]

Isolated inhibitory pool

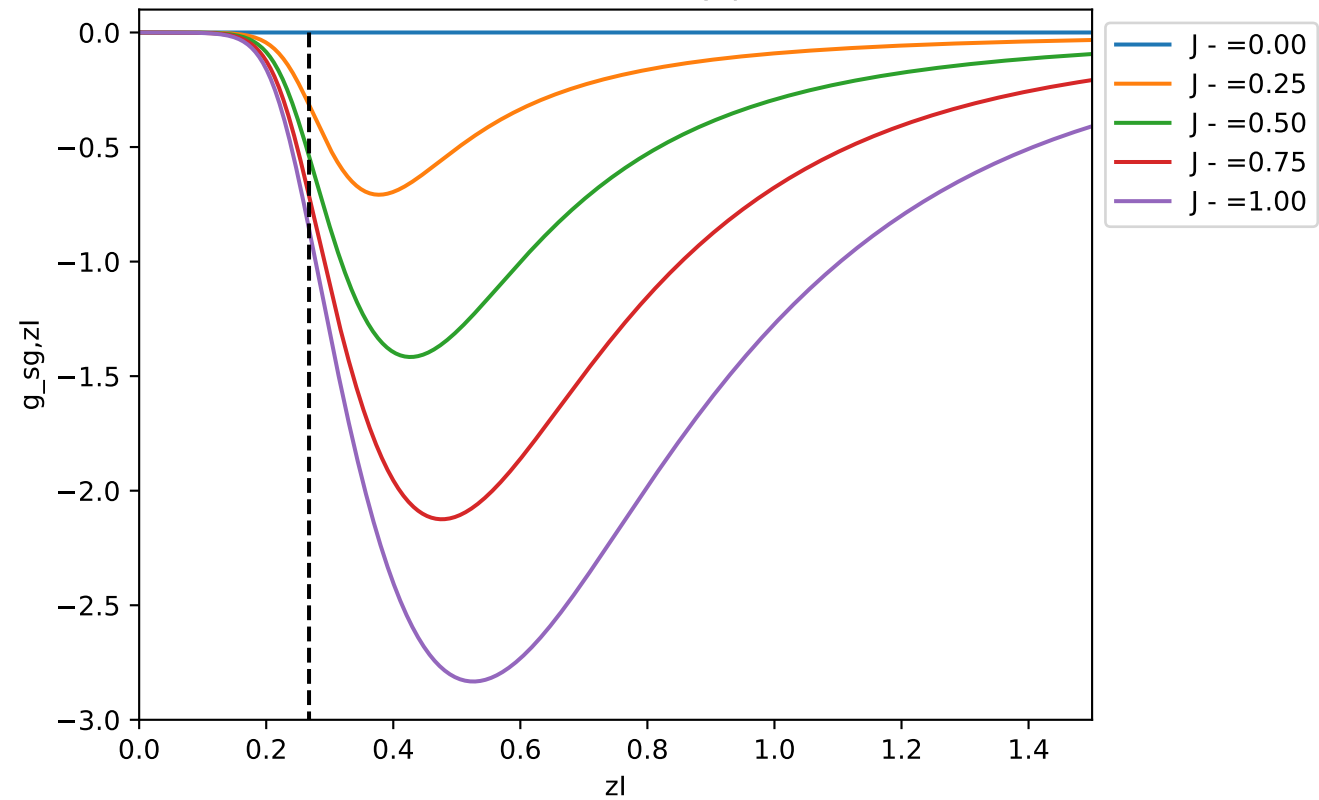

Supplement: S3 File — (ZIP) [file pcbi.1014035.s003.zip › 01-code/05-fig05/Feedback_Gain.pdf]

# Connectivity EE

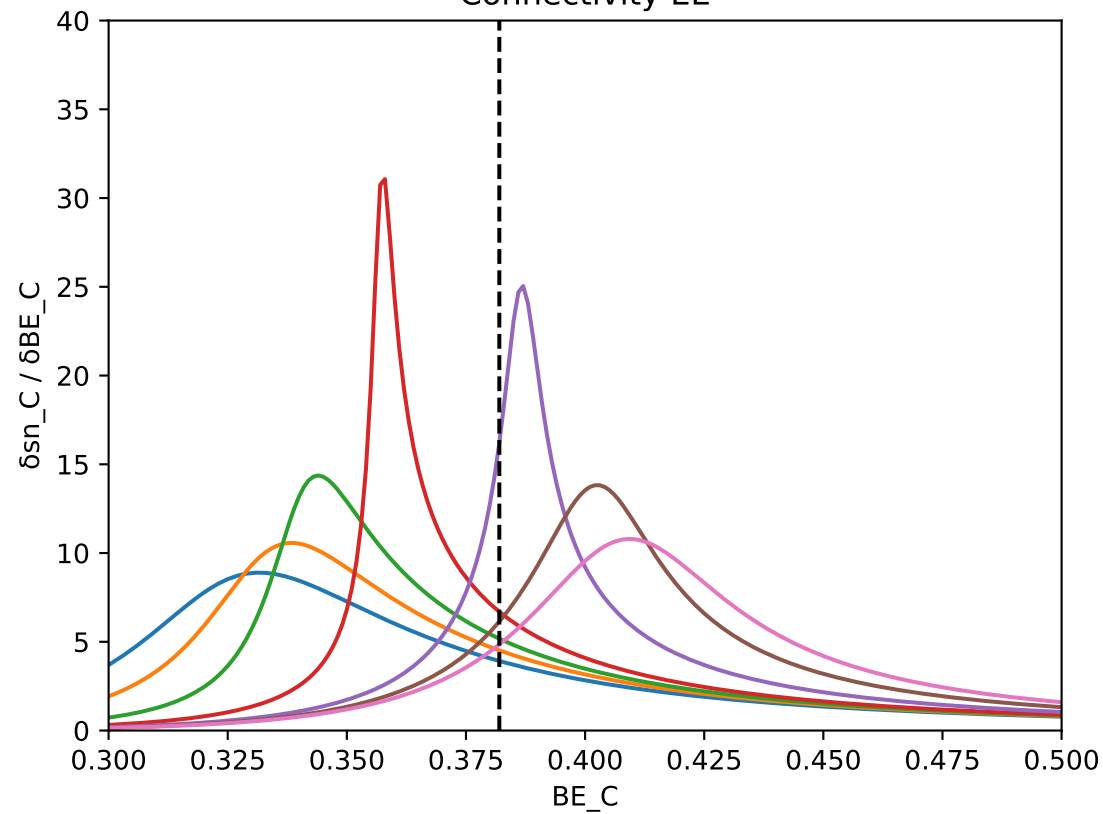

Supplement: S3 File — (ZIP) [file pcbi.1014035.s003.zip › 01-code/08-fig08/Sensi_snC_BEC_EE.pdf]

Connectivity EE

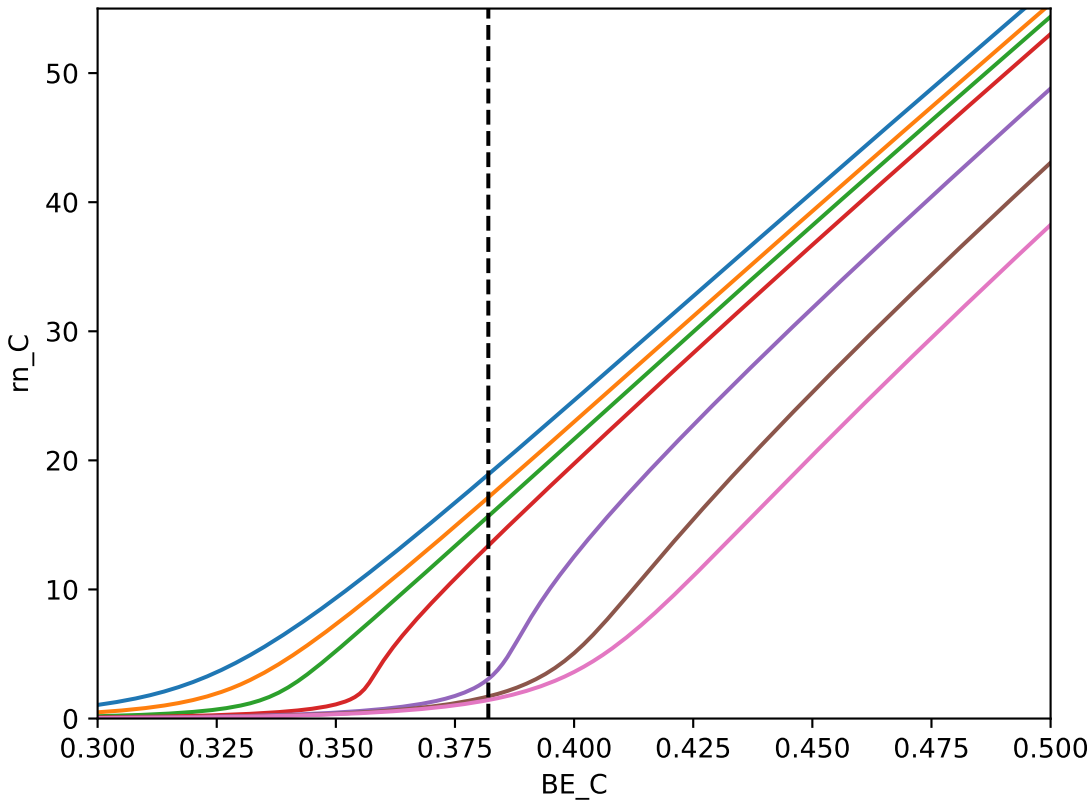

Supplement: S3 File — (ZIP) [file pcbi.1014035.s003.zip › 01-code/08-fig08/rnC_EE.pdf]

Connectivity EE

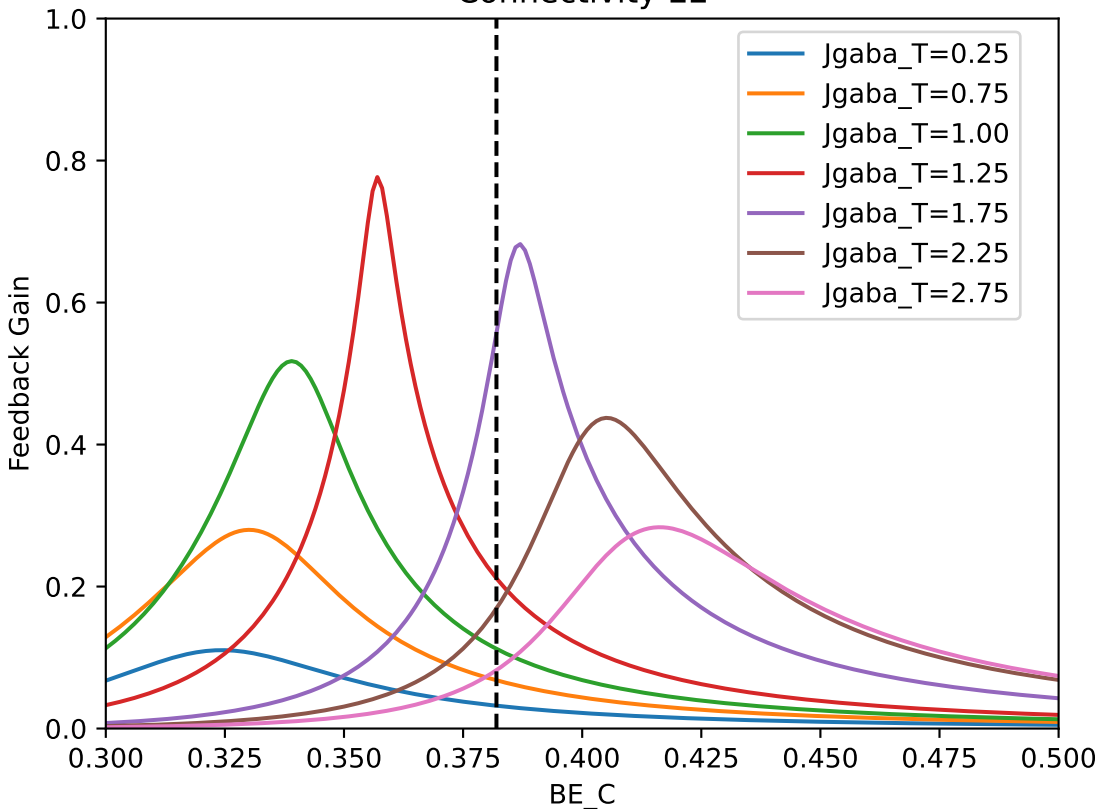

Supplement: S3 File — (ZIP) [file pcbi.1014035.s003.zip › 01-code/08-fig08/FeedbackGain_EE.pdf]

Connectivity EE

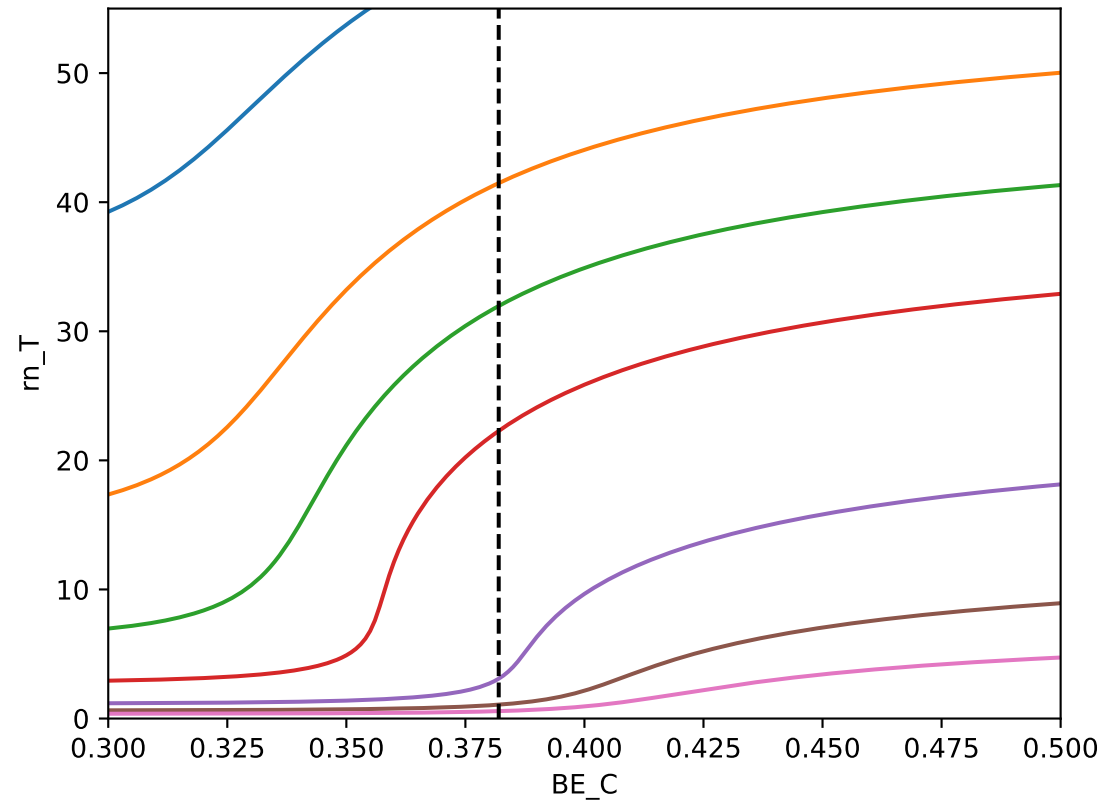

Supplement: S3 File — (ZIP) [file pcbi.1014035.s003.zip › 01-code/08-fig08/rnT_EE.pdf]

# Connectivity EE

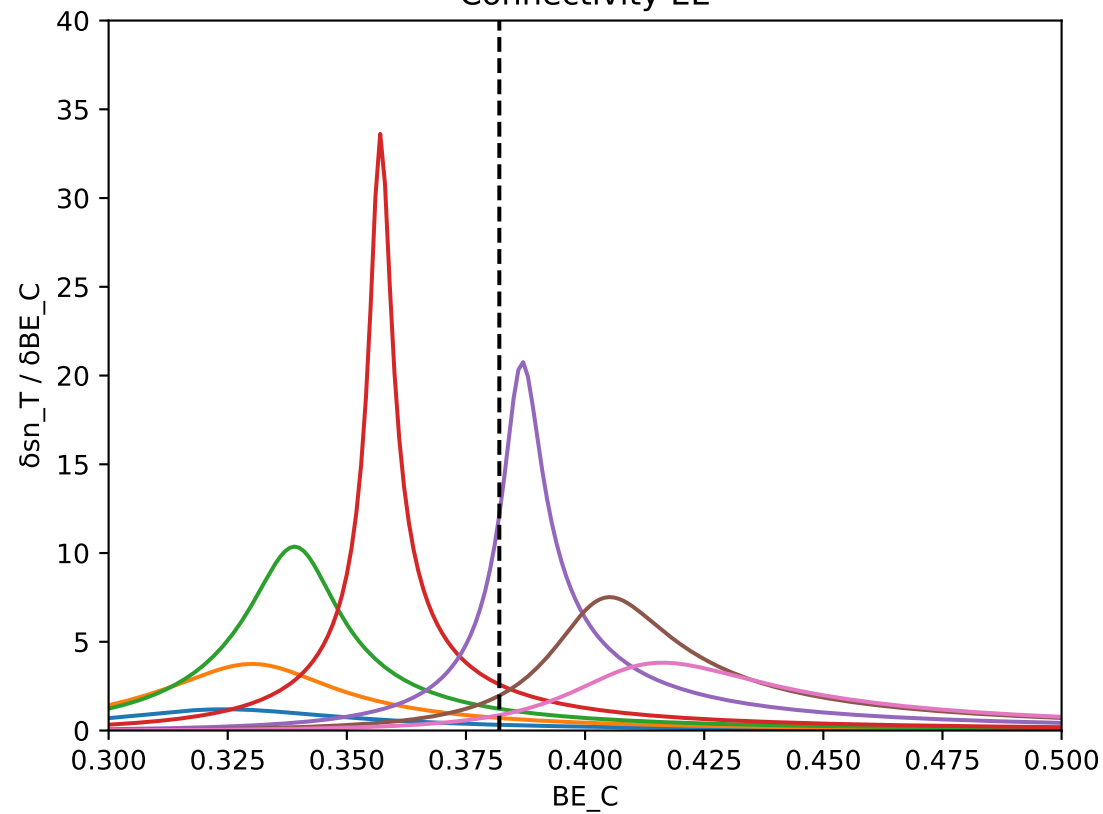

Supplement: S3 File — (ZIP) [file pcbi.1014035.s003.zip › 01-code/08-fig08/Sensi_snT_BEC_EE.pdf]
