# Supplementary material for: Sensitivity analysis enlightens effects of connectivity in a Neural Mass Model under Control-Target mode
Source: PLoS Comput Biol. 2026 Mar 23;22(3):e1014035. doi: 10.1371/journal.pcbi.1014035 (PMC13008111; doi:10.1371/journal.pcbi.1014035)
Supplement: S4 File — (PDF) [file pcbi.1014035.s004.pdf]

Sensitivity analysis enlightens effects of connectivity in a Neural Mass Model under Control-Target mode: SI Jacobian-based derivations

Vallet Anaïs[1], Blanco Stéphane[2], Chevallier Coline[2,3], Eustache Francis[1], Gautrais Jacques[2,3,*], Grandpeix Jean-Yves[4], Joly Jean-Louis[2], Segobin Shailendra[1], Gagnepain Pierre[1]

1 Normandie Univ, UNICAEN, PSL Research University, EPHE, INSERM, U1077, CHU de Caen, GIP Cyceron, Neuropsychologie et Imagerie de la Mémoire Humaine, 14000 Caen, France

2 LAPLACE, Université de Toulouse, CNRS, INPT, UPS, Toulouse, France

3 Centre de Recherches sur la Cognition Animale (CRCA), Centre de Biologie Intégrative (CBI), Université de Toulouse, CNRS, UPS, France

4 LMD/IPSL, Sorbonne Université, CNRS, École Polytechnique, ENS, Paris, France

* jacques.gautrais@cnrs.fr

In this SI, we show that the analytical expressions of sensitivities w.r.t. forcings (in Sec 1) and the propagation matrix of a linear stability analysis (in Sec 2) are respectively the same when expressed in a brute-force formalism or as a function of open loop sensitivities. We have taken the example of one region for this demonstration.

With a brute-force formalism, the analytical expressions of sensitivities and the elements of the propagation matrix depend explicitly on parameters and the fixed point. They are quite long, complex and not legible. Using our nested methodology, they are more compact and legible, as we can give physical meaning to certain terms.

Contents

1 Jacobian-based sensitivity analysis for one isolated area **3**

1.1 Stationnary model 3

1.2 Expressing sensitivities w.r.t. forcings 4

2 Jacobian-based linear stability analysis for one isolated area **11**

2.1 Dynamic model 11

2.2 Expressing the propagation matrix 12

1 Jacobian-based sensitivity analysis for one isolated area

1.1 Stationnary model

At the fixed point, we start with the system for one isolated area:

$$\begin{cases} 0 = -\beta^E sn^* + \alpha^E T_{glu}(1 - sn^*)rn^* \equiv fn(sn^*, rn^*) \\ 0 = -\beta^I sg^* + \alpha^I T_{gaba}(1 - sg^*)rg^* \equiv fg(sg^*, rg^*) \end{cases} \tag{1}$$

with

$$\begin{cases} rn^* = \dfrac{a_E xn^* - b_E}{1 - e^{-d_E(a_E xn^* - b_E)}} \equiv hn(xn^*) \\ rg^* = \dfrac{a_I xg^* - b_I}{1 - e^{-d_I(a_I xg^* - b_I)}} \equiv hg(xg^*) \end{cases} \tag{2}$$

in which xn^* and xg^* represent the respective total input currents:

$$\begin{cases} xn^* = W_+ J_{nmda} sn^* - J_{gaba} sg^* + x_E \equiv wn(sn^*, sg^*, x_E) \\ xg^* = J_{nmda} sn^* - J_- sg^* + x_I \equiv wn(sn^*, sg^*, x_I) \end{cases} \tag{3}$$

where x_E and x_I represent basal forcings (effective external inputs), that we will respectively perturbate.

We define

$$\vec{s} = \begin{pmatrix} sn^* \\ sg^* \end{pmatrix}, \quad \vec{r} = \begin{pmatrix} rn^* \\ rg^* \end{pmatrix}, \quad \vec{x} = \begin{pmatrix} xn^* \\ xg^* \end{pmatrix} \tag{4}$$

so that we can summarize the fixed point as:

$$\begin{cases} \vec{f}(\vec{s}, \vec{r}) = \vec{0} \\ \vec{r} = \vec{h}(\vec{x}) \\ \vec{x} = \vec{w}(\vec{s}, x_E, x_I) \end{cases} \tag{5}$$

1.2 Expressing sensitivities w.r.t. forcings

We start from Eq 5 where we consider the dependency to x_E.

With the perturbation, we get the new model:

$$\begin{cases} \vec{f}(\vec{s},\vec{r}) = \vec{0} \\ \vec{r} = \vec{h}(\vec{x}) \\ \vec{x} = \vec{w}(\vec{s}, x_E + \delta x_E, x_I) \end{cases} \tag{6}$$

We search the formal expression for the sensitivities of state variables w.r.t. forcings:

$$\begin{pmatrix} \mathscr{A}_{sn,x_E} \\ \\ \mathscr{A}_{sg,x_E} \end{pmatrix} \equiv \begin{pmatrix} \frac{\delta sn}{\delta x_E} \\ \\ \frac{\delta sg}{\delta x_E} \end{pmatrix} = \overrightarrow{\frac{\delta s}{\delta x_E}} \tag{7}$$

The linearization for the perturbation around fixed points $\vec{s^*} = (sn^*, sg^*)$ yields:

$$\begin{cases} \overline{\overline{\frac{\partial f}{\partial s}}}\vec{\delta s} + \overline{\overline{\frac{\partial f}{\partial r}}}\vec{\delta r} = \vec{0} \\ \vec{\delta r} = \overline{\overline{\frac{\partial h}{\partial x}}}\vec{\delta x} \\ \vec{\delta x} = \overline{\overline{\frac{\partial w}{\partial s}}}\vec{\delta s} + \overline{\overline{\frac{\partial w}{\partial x_E}}}\overrightarrow{\delta x_E} \end{cases} \tag{8}$$

Plugging the two last expressions into the first, we get:

$$\left(\overline{\overline{\frac{\partial f}{\partial s}}} + \overline{\overline{\frac{\partial f}{\partial r}\frac{\partial h}{\partial x}\frac{\partial w}{\partial s}}}\right)\vec{\delta s} = -\overline{\overline{\frac{\partial f}{\partial r}\frac{\partial h}{\partial x}\frac{\partial w}{\partial x_E}}}\overrightarrow{\delta x_E} \tag{9}$$

$$\left(\overline{\overline{\mathbb{I}}} + \overline{\overline{\frac{\partial f}{\partial s}}}^{-1}\overline{\overline{\frac{\partial f}{\partial r}\frac{\partial h}{\partial x}\frac{\partial w}{\partial s}}}\right)\vec{\delta s} = -\overline{\overline{\frac{\partial f}{\partial s}}}^{-1}\overline{\overline{\frac{\partial f}{\partial r}\frac{\partial h}{\partial x}\frac{\partial w}{\partial x_E}}}\overrightarrow{\delta x_E} \tag{10}$$

so that we can write the sensitivities w.r.t the excitatory forcing x_E:

$$\begin{pmatrix} \mathscr{A}_{sn,x_E} \\ \\ \mathscr{A}_{sg,x_E} \end{pmatrix} = \overrightarrow{\frac{\delta s}{\delta x_E}} = -\left(\bar{\bar{\mathbb{I}}} + \overline{\overline{\frac{\partial f}{\partial s}}}^{-1} \overline{\overline{\frac{\partial f}{\partial r}\frac{\partial h}{\partial x}\frac{\partial w}{\partial s}}}\right)^{-1} \overline{\overline{\frac{\partial f}{\partial s}}}^{-1} \overline{\overline{\frac{\partial f}{\partial r}\frac{\partial h}{\partial x}\frac{\partial w}{\partial x_E}}} \tag{11}$$

Now turning to x_I, we can proceed the same way, expressing at fixed point the model with pertubation:

$$\begin{cases} \vec{f}(\vec{s}, \vec{r}) = \vec{0} \\ \vec{r} = \vec{h}(\vec{x}) \\ \vec{x} = \vec{w}(\vec{s}, x_E, x_I + \delta x_I) \end{cases} \tag{12}$$

and we obtain the sensitivities w.r.t. the inhibitory forcing x_I:

$$\begin{pmatrix} \mathscr{A}_{sn,x_I} \\ \\ \mathscr{A}_{sg,x_I} \end{pmatrix} \equiv \overrightarrow{\frac{\delta s}{\delta x_I}} = -\left(\bar{\bar{\mathbb{I}}} + \overline{\overline{\frac{\partial f}{\partial s}}}^{-1} \overline{\overline{\frac{\partial f}{\partial r}\frac{\partial h}{\partial x}\frac{\partial w}{\partial s}}}\right)^{-1} \overline{\overline{\frac{\partial f}{\partial s}}}^{-1} \overline{\overline{\frac{\partial f}{\partial r}\frac{\partial h}{\partial x}\frac{\partial w}{\partial x_I}}} \tag{13}$$

Since we have:

$$\overline{\overline{\frac{\partial w}{\partial x_E}}} = \begin{bmatrix} 1 \\ \\ 0 \end{bmatrix}, \overline{\overline{\frac{\partial w}{\partial x_I}}} = \begin{bmatrix} 0 \\ \\ 1 \end{bmatrix} \tag{14}$$

We deduce that:

$$-\left(\bar{\bar{\mathbb{I}}} + \overline{\overline{\frac{\partial f}{\partial s}}}^{-1} \overline{\overline{\frac{\partial f}{\partial r}\frac{\partial h}{\partial x}\frac{\partial w}{\partial s}}}\right)^{-1} \overline{\overline{\frac{\partial f}{\partial s}}}^{-1} \overline{\overline{\frac{\partial f}{\partial r}\frac{\partial h}{\partial x}}} = \left(\overrightarrow{\tfrac{\delta s}{\delta x_E}}, \overrightarrow{\tfrac{\delta s}{\delta x_I}}\right) = \begin{pmatrix} \mathscr{A}_{sn,x_E} & \mathscr{A}_{sn,x_I} \\ \\ \mathscr{A}_{sg,x_E} & \mathscr{A}_{sg,x_I} \end{pmatrix} \tag{15}$$

Now we can calculate the different terms of equation 15 in order to formally express the sensitivities :

$$\overline{\overline{\frac{\partial f}{\partial s}}} = \begin{bmatrix} \frac{\partial fn}{\partial sn} & 0 \\ 0 & \frac{\partial fg}{\partial sg} \end{bmatrix} = \begin{bmatrix} -\beta^E - \alpha^E T_{glu} rn^* & 0 \\ 0 & -\beta^I - \alpha^I T_{gaba} rg^* \end{bmatrix} \tag{16}$$

$$\overline{\overline{\frac{\partial f}{\partial s}}}^{-1} = \begin{bmatrix} (-\beta^E - \alpha^E T_{glu} rn^*)^{-1} & 0 \\ 0 & (-\beta^I - \alpha^I T_{gaba} rg^*)^{-1} \end{bmatrix} \tag{17}$$

$$\overline{\overline{\frac{\partial f}{\partial r}}} = \begin{bmatrix} \frac{\partial fn}{\partial rn} & 0 \\ 0 & \frac{\partial fg}{\partial rg} \end{bmatrix} = \begin{bmatrix} \alpha^E T_{glu}(1 - sn^*) & 0 \\ 0 & \alpha^I T_{gaba}(1 - sg^*) \end{bmatrix} \tag{18}$$

$$\overline{\overline{\frac{\partial h}{\partial x}}} = \begin{bmatrix} \frac{dhn}{dxn^*} & 0 \\ 0 & \frac{dhg}{dxg^*} \end{bmatrix} \equiv \begin{bmatrix} hn'(xn^*) & 0 \\ 0 & hg'(xg^*) \end{bmatrix} \tag{19}$$

Then we have:

$$\overline{\overline{\frac{\partial f}{\partial r}\frac{\partial h}{\partial x}}} = \begin{bmatrix} \alpha^E T_{glu}(1 - sn^*) hn'(xn^*) & 0 \\ 0 & \alpha^I T_{gaba}(1 - sg^*) hg'(xg^*) \end{bmatrix} \tag{20}$$

and

$$\overline{\overline{\frac{\partial f}{\partial s}}}^{-1} \overline{\overline{\frac{\partial f}{\partial r}\frac{\partial h}{\partial x}}} = \begin{bmatrix} -\frac{\alpha^E T_{glu}(1-sn^*) hn'(xn^*)}{\beta^E + \alpha^E T_{glu} rn^*} & 0 \\ 0 & -\frac{\alpha^I T_{gaba}(1-sg^*) hg'(xg^*)}{\beta^I + \alpha^I T_{gaba} rg^*} \end{bmatrix} \tag{21}$$

We denote the following expressions of parameters and fixed point values:

$$\begin{cases} \xi_n \equiv \dfrac{\beta^E - \alpha^E T_{glu} rn^*}{\alpha^E T_{glu}(1 - sn^*) hn'(xn^*)} \\ \xi_g \equiv \dfrac{\beta^I - \alpha^I T_{gaba} rg^*}{\alpha^I T_{gaba}(1 - sg^*) hg'(xg^*)} \end{cases} \tag{22}$$

So we can write:

$$\overline{\overline{\frac{\partial f}{\partial s}}}^{-1} \overline{\overline{\frac{\partial f}{\partial r}\frac{\partial h}{\partial x}}} = \begin{bmatrix} -\frac{1}{\xi_n} & 0 \\ 0 & -\frac{1}{\xi_g} \end{bmatrix} \tag{23}$$

Furthermore the matrix that contains the couplings is expressed as:

$$\overline{\overline{\frac{\partial w}{\partial s}}} = \begin{bmatrix} \frac{\partial wn}{\partial sn^*} & \frac{\partial wn}{\partial sg^*} \\ \frac{\partial wg}{\partial sn^*} & \frac{\partial wg}{\partial sg^*} \end{bmatrix} \equiv \begin{bmatrix} W_+ J_{nmda} & -J_{gaba} \\ J_{nmda} & -J_- \end{bmatrix} \tag{24}$$

Which gives:

$$\overline{\overline{\frac{\partial f}{\partial s}}}^{-1} \overline{\overline{\frac{\partial f}{\partial r}\frac{\partial h}{\partial x}\frac{\partial w}{\partial s}}} = \begin{bmatrix} -\frac{1}{\xi_n} & 0 \\ 0 & -\frac{1}{\xi_g} \end{bmatrix} \begin{bmatrix} W_+ J_{nmda} & -J_{gaba} \\ J_{nmda} & -J_- \end{bmatrix} \tag{25}$$

$$= \begin{bmatrix} -\frac{W_+ J_{nmda}}{\xi_n} & \frac{J_{gaba}}{\xi_n} \\ -\frac{J_{nmda}}{\xi_g} & \frac{J_-}{\xi_g} \end{bmatrix} \tag{26}$$

and so we have:

$$\left(\overline{\overline{\mathbb{I}}} + \overline{\overline{\frac{\partial f}{\partial s}}}^{-1} \overline{\overline{\frac{\partial f}{\partial r}\frac{\partial h}{\partial x}\frac{\partial w}{\partial s}}} \right)^{-1} = \begin{bmatrix} 1 - \frac{W_+ J_{nmda}}{\xi_n} & \frac{J_{gaba}}{\xi_n} \\ -\frac{J_{nmda}}{\xi_g} & 1 + \frac{J_-}{\xi_g} \end{bmatrix}^{-1} \tag{27}$$

$$= \frac{1}{(1 - \frac{W_+ J_{nmda}}{\xi_n})(1 + \frac{J_-}{\xi_g}) + \frac{J_{gaba}}{\xi_n}\frac{J_{nmda}}{\xi_g}} \begin{bmatrix} 1 + \frac{J_-}{\xi_g} & -\frac{J_{gaba}}{\xi_n} \\ \frac{J_{nmda}}{\xi_g} & 1 - \frac{W_+ J_{nmda}}{\xi_n} \end{bmatrix} \tag{28}$$

Finally, we calculate the matrix that contains the sensitivities w.r.t. forcings:

$$\begin{bmatrix} \mathscr{A}_{sn,x_E} & \mathscr{A}_{sn,x_I} \\ \\ \mathscr{A}_{sg,x_E} & \mathscr{A}_{sg,x_I} \end{bmatrix} \tag{29}$$

$$= -\left(\bar{\bar{\mathbb{I}}} + \overline{\overline{\frac{\partial f}{\partial s}}}^{-1} \overline{\overline{\frac{\partial f}{\partial r} \frac{\partial h}{\partial x} \frac{\partial w}{\partial s}}} \right)^{-1} \overline{\overline{\frac{\partial f}{\partial s}}}^{-1} \overline{\overline{\frac{\partial f}{\partial r} \frac{\partial h}{\partial x}}} \tag{30}$$

$$= -\frac{1}{(1 - \frac{W_+ J_{nmda}}{\xi_n})(1 + \frac{J_-}{\xi_g}) + \frac{J_{gaba}}{\xi_n}\frac{J_{nmda}}{\xi_g}} \begin{bmatrix} 1 + \frac{J_-}{\xi_g} & -\frac{J_{gaba}}{\xi_n} \\ \frac{J_{nmda}}{\xi_g} & 1 - \frac{W_+ J_{nmda}}{\xi_n} \end{bmatrix} \begin{bmatrix} -\frac{1}{\xi_n} & 0 \\ 0 & -\frac{1}{\xi_g} \end{bmatrix} \tag{31}$$

$$= -\frac{1}{(1 - \frac{W_+ J_{nmda}}{\xi_n})(1 + \frac{J_-}{\xi_g}) + \frac{J_{gaba}}{\xi_n}\frac{J_{nmda}}{\xi_g}} \begin{bmatrix} (1 + \frac{J_-}{\xi_g})(-\frac{1}{\xi_n}) & (-\frac{J_{gaba}}{\xi_n})(-\frac{1}{\xi_g}) \\ \frac{J_{nmda}}{\xi_g}(-\frac{1}{\xi_n}) & (1 - \frac{W_+ J_{nmda}}{\xi_n})(-\frac{1}{\xi_g}) \end{bmatrix} \tag{32}$$

$$= \frac{1}{(1 - \frac{W_+ J_{nmda}}{\xi_n})(1 + \frac{J_-}{\xi_g}) + \frac{J_{gaba}}{\xi_n}\frac{J_{nmda}}{\xi_g}} \begin{bmatrix} (1 + \frac{J_-}{\xi_g})\frac{1}{\xi_n} & -\frac{J_{gaba}}{\xi_n}\frac{1}{\xi_g} \\ \frac{J_{nmda}}{\xi_g}\frac{1}{\xi_n} & (1 - \frac{W_+ J_{nmda}}{\xi_n})\frac{1}{\xi_g} \end{bmatrix} \tag{33}$$

Replacing ξ_n and ξ_g with their expressions in equation 22, we get a complex analytical expression in which the different terms have no meaning. It depends on the fixed point and the parameters:

$$\begin{bmatrix} \mathscr{A}_{sn,x_E} & \mathscr{A}_{sn,x_I} \\ \\ \mathscr{A}_{sg,x_E} & \mathscr{A}_{sg,x_I} \end{bmatrix}$$

$$= \frac{1}{\left(1 - W_+ J_{nmda} \frac{\alpha^E T_{glu}(1-sn^*)hn'(xn^*)}{\beta^E - \alpha^E T_{glu} rn^*}\right)\left(1 + (J_-)\frac{\alpha^I T_{gaba}(1-sg^*)hg'(xg^*)}{\beta^I - \alpha^I T_{gaba} rg^*}\right) + J_{gaba}\frac{\alpha^E T_{glu}(1-sn^*)hn'(xn^*)}{\beta^E - \alpha^E T_{glu} rn^*} J_{nmda}\frac{\alpha^I T_{gaba}(1-sg^*)hg'(xg^*)}{\beta^I - \alpha^I T_{gaba} rg^*}}$$

$$\begin{bmatrix} \left(1 + (J_-)\frac{\alpha^I T_{gaba}(1-sg^*)hg'(xg^*)}{\beta^I - \alpha^I T_{gaba} rg^*}\right)\frac{\alpha^E T_{glu}(1-sn^*)hn'(xn^*)}{\beta^E - \alpha^E T_{glu} rn^*} & -J_{gaba}\frac{\alpha^E T_{glu}(1-sn^*)hn'(xn^*)}{\beta^E - \alpha^E T_{glu} rn^*}\frac{\alpha^I T_{gaba}(1-sg^*)hg'(xg^*)}{\beta^I - \alpha^I T_{gaba} rg^*} \\ J_{nmda}\frac{\alpha^I T_{gaba}(1-sg^*)hg'(xg^*)}{\beta^I - \alpha^I T_{gaba} rg^*}\frac{\alpha^E T_{glu}(1-sn^*)hn'(xn^*)}{\beta^E - \alpha^E T_{glu} rn^*} & \left(1 - W_+ J_{nmda}\frac{\alpha^E T_{glu}(1-sn^*)hn'(xn^*)}{\beta^E - \alpha^E T_{glu} rn^*}\right)\frac{\alpha^I T_{gaba}(1-sg^*)hg'(xg^*)}{\beta^I - \alpha^I T_{gaba} rg^*} \end{bmatrix} \tag{34}$$

We show that the expressions of the sensitivities w.r.t. forcings (expressed in equation 33) are exactly equal to those presented in section **??**.

First we can multiply the numerator and the denominator by ξ_n:

$$\begin{bmatrix} \mathscr{A}_{sn,x_E} & \mathscr{A}_{sn,x_I} \\ \\ \mathscr{A}_{sg,x_E} & \mathscr{A}_{sg,x_I} \end{bmatrix} \tag{35}$$

$$= \frac{1}{(1-\frac{W_+ J_{nmda}}{\xi_n})(1+\frac{J_-}{\xi_g}) + \frac{J_{gaba}}{\xi_n}\frac{J_{nmda}}{\xi_g}} \begin{bmatrix} (1+\frac{J_-}{\xi_g})\frac{1}{\xi_n} & -\frac{J_{gaba}}{\xi_n}\frac{1}{\xi_g} \\ \frac{J_{nmda}}{\xi_g}\frac{1}{\xi_n} & (1-\frac{W_+ J_{nmda}}{\xi_n})\frac{1}{\xi_g} \end{bmatrix} \tag{36}$$

$$= \frac{1}{(\xi_n - W_+ J_{nmda})(1+\frac{J_-}{\xi_g}) + J_{gaba}\frac{J_{nmda}}{\xi_g}} \begin{bmatrix} (1+\frac{J_-}{\xi_g}) & -J_{gaba}\frac{1}{\xi_g} \\ \frac{J_{nmda}}{\xi_g} & (\xi_n - W_+ J_{nmda})\frac{1}{\xi_g} \end{bmatrix} \tag{37}$$

Second we multiply the numerator and the denominator by ξ_g:

$$\begin{bmatrix} \mathscr{A}_{sn,x_E} & \mathscr{A}_{sn,x_I} \\ \\ \mathscr{A}_{sg,x_E} & \mathscr{A}_{sg,x_I} \end{bmatrix} \tag{38}$$

$$= \frac{1}{(\xi_n - W_+ J_{nmda})(\xi_g + J_-) + J_{gaba} J_{nmda}} \begin{bmatrix} (\xi_g + J_-) & -J_{gaba} \\ J_{nmda} & (\xi_n - W_+ J_{nmda}) \end{bmatrix} \tag{39}$$

Finally, we multiply the numerator and the denominator by $(\xi_n - W_+ J_{nmda})^{-1}(\xi_g + J_-)^{-1}$:

$$\begin{bmatrix} \mathscr{A}_{sn,x_E} & \mathscr{A}_{sn,x_I} \\ \mathscr{A}_{sg,x_E} & \mathscr{A}_{sg,x_I} \end{bmatrix} = \frac{1}{1 + J_{gaba} J_{nmda} (\xi_n - W_+ J_{nmda})^{-1} (\xi_g + J_-)^{-1}} \begin{bmatrix} (\xi_n - W_+ J_{nmda})^{-1} & -J_{gaba} (\xi_n - W_+ J_{nmda})^{-1} (\xi_g + J_-)^{-1} \\ J_{nmda} (\xi_n - W_+ J_{nmda})^{-1} (\xi_g + J_-)^{-1} & (\xi_g + J_-)^{-1} \end{bmatrix} \tag{40}$$

In Sec **??**, we have written:

$$(\xi_n - W_+ J_{nmda})^{-1} \equiv \mathscr{A}^O_{sn,x_E} \tag{41}$$

$$(\xi_g + J_-)^{-1} \equiv \mathscr{A}^O_{sg,x_I} \tag{42}$$

Note that $\mathscr{A}^O_{sn,x_E}$ (respectively $\mathscr{A}^O_{sg,x_I}$) has physical meaning as it refers to the open loop sensitivity of the area, i.e. the sensitivity of the excitatory (resp. inhibitory) pool w.r.t. the excitatory (resp. inhibitory) forcing, evaluated at the fixed point of the closed loop area.

Equation 40 can then be rewritten as:

$$\begin{bmatrix} \mathscr{A}_{sn,x_E} & \mathscr{A}_{sn,x_I} \\ \mathscr{A}_{sg,x_E} & \mathscr{A}_{sg,x_I} \end{bmatrix} = \frac{1}{1 + J_{gaba} J_{nmda} \mathscr{A}^O_{sn,x_E} \mathscr{A}^O_{sg,x_I}} \begin{bmatrix} \mathscr{A}^O_{sn,x_E} & -J_{gaba} \mathscr{A}^O_{sn,x_E} \mathscr{A}^O_{sg,x_I} \\ J_{nmda} \mathscr{A}^O_{sn,x_E} \mathscr{A}^O_{sg,x_I} & \mathscr{A}^O_{sg,x_I} \end{bmatrix} \tag{43}$$

We therefore find the same expressions of sensitivities w.r.t. forcings as when using nested sensitivities.

2 Jacobian-based linear stability analysis for one isolated area

2.1 Dynamic model

The dynamic system can be expressed as:

$$\begin{cases} \dfrac{dsn(t)}{dt} = -\beta^E sn(t) + \alpha^E T_{glu}(1 - sn(t))rn(t) & \equiv fn(sn(t), rn(t)) \\ \dfrac{dsg(t)}{dt} = -\beta^I sg(t) + \alpha^I T_{gaba}(1 - sg(t))rg(t) & \equiv fg(sn(t), rn(t)) \end{cases} \tag{44}$$

with

$$\begin{cases} rn(t) = \dfrac{a_E xn(t) - b_E}{1 - e^{-d_E(a_E xn(t) - b_E)}} & \equiv hn(xn(t)) \\ rg(t) = \dfrac{a_I xg(t) - b_I}{1 - e^{-d_I(a_I xg(t) - b_I)}} & \equiv hg(xg(t)) \end{cases} \tag{45}$$

In Eq 45, $xn(t)$ and $xg(t)$ represent the respective total input current:

$$\begin{cases} xn(t) = W_+ J_{nmda} sn(t) - J_{gaba} sg(t) + x_E \\ xg(t) = J_{nmda} sn(t) - J_- sg(t) + x_I \end{cases} \tag{46}$$

where x_E and x_I represent basal forcings (effective external inputs).

We define

$$\vec{s}(t) = \begin{pmatrix} sn(t) \\ sg(t) \end{pmatrix}, \quad \vec{r}(t) = \begin{pmatrix} rn(t) \\ rg(t) \end{pmatrix}, \quad \vec{x}(t) = \begin{pmatrix} xn(t) \\ xg(t) \end{pmatrix} \tag{47}$$

The dynamic model can thus be rewritten in vectorised form:

$$\begin{cases} \dfrac{d\vec{s}}{dt} = \vec{f}(\vec{s}, \vec{r}) \\ \vec{r} = \vec{h}(\vec{x}) \\ \vec{x} = \vec{w}(\vec{s}, x_E, x_I) \end{cases} \tag{48}$$

2.2 Expressing the propagation matrix

We stand near the fixed point: $\vec{s} = \vec{s^*} + \vec{\delta s}$.

The perturbed form of system 48 reads:

$$\begin{cases} \dfrac{d}{dt}(\vec{s^*} + \vec{\delta s}) = \vec{f}\,(\vec{s^*} + \vec{\delta s}, \vec{r}\,) \\ \qquad\qquad \vec{r} = \vec{h}\,(\vec{x}\,) \\ \qquad\qquad \vec{x} = \vec{w}(\vec{s^*} + \vec{\delta s}, x_E, x_I) \end{cases} \tag{49}$$

By linearization near the fixed point, we get:

$$\begin{cases} \dfrac{d}{dt}(\vec{\delta s}) = \overline{\overline{\dfrac{\partial f}{\partial s}^*}}\vec{\delta s} + \overline{\overline{\dfrac{\partial f}{\partial r}^*}}\vec{\delta r} \\ \quad \vec{\delta r} = \overline{\overline{\dfrac{\partial h}{\partial x}^*}}\vec{\delta x} \\ \quad \vec{\delta x} = \overline{\overline{\dfrac{\partial w}{\partial s}^*}}\vec{\delta s} \end{cases} \tag{50}$$

Plugging the second equation into the first, we have:

$$\frac{d}{dt}(\vec{\delta s}) = \overline{\overline{\frac{\partial f}{\partial s}^*}}\vec{\delta s} + \overline{\overline{\frac{\partial f}{\partial r}^*\frac{\partial h}{\partial x}^*\frac{\partial w}{\partial s}^*}}\vec{\delta s} \tag{51}$$

$$= \left(\overline{\overline{\frac{\partial f}{\partial s}^*}} + \overline{\overline{\frac{\partial f}{\partial r}^*\frac{\partial h}{\partial x}^*\frac{\partial w}{\partial s}^*}}\right)\vec{\delta s} \tag{52}$$

$$= \overline{\overline{\frac{\partial f}{\partial r}^*\frac{\partial h}{\partial x}^*}}\left(\left(\overline{\overline{\frac{\partial f}{\partial r}\frac{\partial h}{\partial x}}}\right)^{-1}\overline{\overline{\frac{\partial f}{\partial s}^*}} + \overline{\overline{\frac{\partial w}{\partial s}^*}}\right)\vec{\delta s} \tag{53}$$

We denote $\overline{\overline{J}}$ the propagation matrix:

$$\overline{\overline{J}} = \overline{\overline{\frac{\partial f}{\partial r}^*\frac{\partial h}{\partial x}^*}}\left(\left(\overline{\overline{\frac{\partial f}{\partial r}\frac{\partial h}{\partial x}}}\right)^{-1}\overline{\overline{\frac{\partial f}{\partial s}^*}} + \overline{\overline{\frac{\partial w}{\partial s}^*}}\right) \tag{54}$$

We have:

$$\overline{\overline{\frac{\partial f}{\partial s}}} = \begin{bmatrix} \frac{\partial fn}{\partial sn} & 0 \\ 0 & \frac{\partial fg}{\partial sg} \end{bmatrix} = \begin{bmatrix} -\beta^E - \alpha^E T_{glu} rn^* & 0 \\ 0 & -\beta^I - \alpha^I T_{gaba} rg^* \end{bmatrix} \tag{55}$$

$$\overline{\overline{\frac{\partial f}{\partial r}}} = \begin{bmatrix} \frac{\partial fn}{\partial rn} & 0 \\ 0 & \frac{\partial fg}{\partial rg} \end{bmatrix} = \begin{bmatrix} \alpha^E T_{glu}(1 - sn^*) & 0 \\ 0 & \alpha^I T_{gaba}(1 - sg^*) \end{bmatrix} \tag{56}$$

$$\overline{\overline{\frac{\partial h}{\partial x}}} = \begin{bmatrix} \frac{dhn}{dxn^*} & 0 \\ 0 & \frac{dhg}{dxg^*} \end{bmatrix} \equiv \begin{bmatrix} hn'(xn^*) & 0 \\ 0 & hg'(xg^*) \end{bmatrix} \tag{57}$$

Then:

$$\overline{\overline{\frac{\partial f}{\partial r}\frac{\partial h}{\partial x}}} = \begin{bmatrix} \alpha^E T_{glu}(1 - sn^*) hn'(xn^*) & 0 \\ 0 & \alpha^I T_{gaba}(1 - sg^*) hg'(xg^*) \end{bmatrix} \tag{58}$$

Denoting:

$$\begin{cases} \xi_n \equiv \dfrac{\beta^E - \alpha^E T_{glu} rn^*}{\alpha^E T_{glu}(1 - sn^*) hn'(xn^*)} \\ \xi_g \equiv \dfrac{\beta^I - \alpha^I T_{gaba} rg^*}{\alpha^I T_{gaba}(1 - sg^*) hg'(xg^*)} \end{cases} \tag{59}$$

We can calculate:

$$\left(\overline{\overline{\frac{\partial f}{\partial r}\frac{\partial h}{\partial x}}}\right)^{-1} \overline{\overline{\frac{\partial f}{\partial s}}}^* = \begin{bmatrix} -\frac{\beta^E - \alpha^E T_{glu} rn^*}{\alpha^E T_{glu}(1-sn^*) hn'(xn^*)} & 0 \\ 0 & -\frac{\beta^I - \alpha^I T_{gaba} rg^*}{\alpha^I T_{gaba}(1-sg^*) hg'(xg^*)} \end{bmatrix} = \begin{bmatrix} -\xi_n & 0 \\ 0 & -\xi_g \end{bmatrix} \tag{60}$$

Furthermore the matrix that contains the couplings is expressed as:

$$\overline{\overline{\frac{\partial w}{\partial s}}} = \begin{bmatrix} \frac{\partial wn}{\partial sn^*} & \frac{\partial wn}{\partial sg^*} \\ \frac{\partial wg}{\partial sn^*} & \frac{\partial wg}{\partial sg^*} \end{bmatrix} \equiv \begin{bmatrix} W_+ J_{nmda} & -J_{gaba} \\ J_{nmda} & -J_- \end{bmatrix} \tag{61}$$

Which gives for the propagation matrix:

$$\overline{\overline{J}} = \begin{bmatrix} \alpha^E T_{glu}(1-sn^*)hn'(xn^*) & 0 \\ 0 & \alpha^I T_{gaba}(1-sg^*)hg'(xg^*) \end{bmatrix} \left(\begin{bmatrix} -\xi_n & 0 \\ 0 & -\xi_g \end{bmatrix} + \begin{bmatrix} W_+ J_{nmda} & -J_{gaba} \\ J_{nmda} & -J_- \end{bmatrix} \right) \tag{62}$$

$$= \begin{bmatrix} \alpha^E T_{glu}(1-sn^*)hn'(xn^*) & 0 \\ 0 & \alpha^I T_{gaba}(1-sg^*)hg'(xg^*) \end{bmatrix} \begin{bmatrix} -\xi_n + W_+ J_{nmda} & -J_{gaba} \\ J_{nmda} & -\xi_g - J_- \end{bmatrix} \tag{63}$$

$$= \begin{bmatrix} \alpha^E T_{glu}(1-sn^*)hn'(xn^*)(-\xi_n + W_+ J_{nmda}) & -\alpha^E T_{glu}(1-sn^*)hn'(xn^*)J_{gaba} \\ \alpha^I T_{gaba}(1-sg^*)hg'(xg^*)J_{nmda} & \alpha^I T_{gaba}(1-sg^*)hg'(xg^*)(-\xi_g - J_-) \end{bmatrix} \tag{64}$$

$$= \begin{bmatrix} -\alpha^E T_{glu}(1-sn^*)hn'(xn^*)(\xi_n - W_+ J_{nmda}) & -\alpha^E T_{glu}(1-sn^*)hn'(xn^*)J_{gaba} \\ \alpha^I T_{gaba}(1-sg^*)hg'(xg^*)J_{nmda} & -\alpha^I T_{gaba}(1-sg^*)hg'(xg^*)(\xi_g + J_-) \end{bmatrix} \tag{65}$$

We show that the propagation matrix is exactly equal to the one presented in Sec **??** as a function of the open loop sensitivities.

In fact, in Sec **??**, we have written:

$$\begin{cases} (\xi_n - W_+ J_{nmda})^{-1} \equiv \mathscr{A}^O_{sn,x_E} \\ (\xi_g + J_-)^{-1} \equiv \mathscr{A}^O_{sg,x_I} \end{cases} \tag{66}$$

Note that $\mathscr{A}^O_{sn,x_E}$ (respectively $\mathscr{A}^O_{sg,x_I}$) has physical meaning as it refers to the open loop sensitivity of the area, i.e. the sensitivity of the excitatory (resp. inhibitory) pool w.r.t. the excitatory (resp. inhibitory) forcing, evaluated at the fixed point of the closed loop area.

Then we deduce:

$$\overline{\overline{J}} = \begin{bmatrix} -\alpha^E T_{glu}(1-sn^*)hn'(xn^*)(\mathscr{A}^O_{sn,x_E})^{-1} & -\alpha^E T_{glu}(1-sn^*)hn'(xn^*)J_{gaba} \\ \alpha^I T_{gaba}(1-sg^*)hg'(xg^*)J_{nmda} & -\alpha^I T_{gaba}(1-sg^*)hg'(xg^*)(\mathscr{A}^O_{sg,x_I})^{-1} \end{bmatrix} \tag{67}$$
