# Supplementary material for: Sensitivity analysis enlightens effects of connectivity in a Neural Mass Model under Control-Target mode
Source: PLoS Comput Biol. 2026 Mar 23;22(3):e1014035. doi: 10.1371/journal.pcbi.1014035 (PMC13008111; doi:10.1371/journal.pcbi.1014035)
Supplement: S5 File — (PDF) [file pcbi.1014035.s005.pdf]

Sensitivity analysis enlightens effects of connectivity in a Neural Mass Model under Control-Target mode: SI Three coupled area system

Vallet Anaïs[1], Blanco Stéphane[2], Chevallier Coline[2,3], Eustache Francis[1], Gautrais Jacques[2,3,*], Grandpeix Jean-Yves[4], Joly Jean-Louis[2], Segobin Shailendra[1], Gagnepain Pierre[1]

1 Normandie Univ, UNICAEN, PSL Research University, EPHE, INSERM, U1077, CHU de Caen, GIP Cyceron, Neuropsychologie et Imagerie de la Mémoire Humaine, 14000 Caen, France

2 LAPLACE, Université de Toulouse, CNRS, INPT, UPS, Toulouse, France

3 Centre de Recherches sur la Cognition Animale (CRCA), Centre de Biologie Intégrative (CBI), Université de Toulouse, CNRS, UPS, France

4 LMD/IPSL, Sorbonne Université, CNRS, École Polytechnique, ENS, Paris, France

* jacques.gautrais@cnrs.fr

Contents

1 Three coupled area system **3**

1.1 Splitting x_{inter} from x_{intra} 3

1.2 Intermediate variables elimination 4

1.3 Expressing sensitivities w.r.t. forcings 5

1.3.1 Perturbation of one forcing B 5

1.3.2 Expressing Open Loop Sensitivities 7

1.4 Using Isolated Area Sensitivities 9

1.5 Sensitivities in a Control-Relay-Target system 14

1 Three coupled area system

1.1 Splitting x_{inter} from x_{intra}

At stationary state, the system can be expressed as:

$$\forall i \in \Sigma = [\![1;3]\!] \begin{cases} 0 = -\beta^E sn_i^* + \alpha^E T_{glu}(1 - sn_i^*) rn_i^* \equiv fn_i(sn_i^*, rn_i^*) \\ 0 = -\beta^I sg_i^* + \alpha^I T_{gaba}(1 - sg_i^*) rg_i^* \equiv fg_i(sn_i^*, rn_i^*) \end{cases} \tag{1}$$

with

$$\begin{cases} rn_i^* = \dfrac{a_E xn_i^* - b_E}{1 - e^{-d_E(a_E xn_i^* - b_E)}} \equiv hn_i(xn_i^*) \\ rg_i^* = \dfrac{a_I xg_i^* - b_I}{1 - e^{-d_I(a_I xg_i^* - b_I)}} \equiv hg_i(xg_i^*) \end{cases} \tag{2}$$

In Eq 1, xn_i^* and xg_i^* represent the respective total input current to area i. In order to express closed loop sensitivities of the areas as a function of their open loop sensitivities, we explicitly split this total input current between an internal current within an area, due to intra-pool recurrence, and coupling between pools:

$$\begin{cases} xn_{intra,i}^* = W_+ J_{nmda} sn_i^* - J_{gaba_i} sg_i^* \equiv wn_{intra,i}(sn_i^*, sg_i^*) \\ xg_{intra,i}^* = J_{nmda} sn_i^* - J_- sg_i^* \equiv wg_{intra,i}(sn_i^*, sg_i^*) \end{cases} \tag{3}$$

and external current due to the external inputs and the coupling between the three areas:

$$\begin{cases} xn_{inter,i}^* = \sum\limits_{\substack{j \in \Sigma \\ j \neq i}} k_{E_{ij}} \kappa_{ij} sn_j + B_{E_i} \equiv wn_{inter,i}(sn_{\{j\} \in \Sigma \setminus \{i\}}^*) \\ xg_{inter,i}^* = \sum\limits_{\substack{j \in \Sigma \\ j \neq i}} (1 - k_{E_{ij}}) \kappa_{ij} sn_j + B_{I_i} \equiv wg_{inter,i}(sn_{\{j\} \in \Sigma \setminus \{i\}}^*) \end{cases} \tag{4}$$

so that total input currents reads:

$$\begin{cases} xn_i^* = xn_{intra,i}^* + xn_{inter,i}^* = wn_i(xn_{intra,i}^*, xn_{inter,i}^*) \\ xg_i^* = xg_{intra,i}^* + xg_{inter,i}^* = wg_i(xg_{intra,i}^*, xg_{inter,i}^*) \end{cases} \tag{5}$$

1.2 Intermediate variables elimination

We define

$$\overrightarrow{s} = \begin{pmatrix} sn_1^* \\ sn_2^* \\ sn_3^* \\ sg_1^* \\ sg_2^* \\ sg_3^* \end{pmatrix}, \quad \overrightarrow{r} = \begin{pmatrix} rn_1^* \\ rn_2^* \\ rn_3^* \\ rg_1^* \\ rg_2^* \\ rg_3^* \end{pmatrix}, \quad \overrightarrow{x} = \begin{pmatrix} xn_1^* \\ xn_2^* \\ xn_3^* \\ xg_1^* \\ xg_2^* \\ xg_3^* \end{pmatrix}, \quad \overrightarrow{x_{intra}} = \begin{pmatrix} xn_{intra,1}^* \\ xn_{intra,2}^* \\ xn_{intra,3}^* \\ xg_{intra,1}^* \\ xg_{intra,2}^* \\ xg_{intra,3}^* \end{pmatrix}, \quad \overrightarrow{x_{inter}} = \begin{pmatrix} xn_{inter,1}^* \\ xn_{inter,2}^* \\ xn_{inter,3}^* \\ xg_{inter,1}^* \\ xg_{inter,2}^* \\ xg_{inter,3}^* \end{pmatrix} \tag{6}$$

and

$$\overrightarrow{B_E} = \begin{pmatrix} B_{E_1} \\ B_{E_2} \\ B_{E_3} \end{pmatrix}, \quad \overrightarrow{B_I} = \begin{pmatrix} B_{I_1} \\ B_{I_2} \\ B_{I_3} \end{pmatrix} \tag{7}$$

so that we can summarize the fixed point as:

$$\begin{cases} \overrightarrow{f}(\overrightarrow{s}, \overrightarrow{r}) = \overrightarrow{0} \\ \overrightarrow{r} = \overrightarrow{h}(\overrightarrow{x}) \\ \overrightarrow{x} = \overrightarrow{w}(\overrightarrow{x_{intra}}, \overrightarrow{x_{inter}}) \\ \overrightarrow{x_{intra}} = \overrightarrow{w_{intra}}(\overrightarrow{s}) \\ \overrightarrow{x_{inter}} = \overrightarrow{w_{inter}}(\overrightarrow{s}, \overrightarrow{B_E}, \overrightarrow{B_I}) \end{cases} \tag{8}$$

Plugging intermediate variables $\overrightarrow{r}$, $\overrightarrow{x}$, $\overrightarrow{x_{intra}}$ into the first equation, Eq 8 can be rewritten as:

$$\begin{cases} \overrightarrow{F}(\overrightarrow{s}, \overrightarrow{x_{inter}}) = \overrightarrow{0} \\ \overrightarrow{x_{inter}} = \overrightarrow{w_{inter}}(\overrightarrow{s}, \overrightarrow{B_E}, \overrightarrow{B_I}) \end{cases} \tag{9}$$

where

$$\begin{cases} Fn_i(sn_i^*, sg_i^*, xn_{inter,i}^*) = -\beta^E sn_i^* \\ \qquad + \alpha^E T_{glu}(1 - sn_i^*) \dfrac{a_E(W_+ J_{nmda} sn_i^* - J_{gaba_i} sg_i^* + xn_{inter,i}^*) - b_E}{1 - e^{-d_E[a_E(W_+ J_{nmda} sn_i^* - J_{gaba_i} sg_i^* + xn_{inter,i}^*) - b_E]}} \\ Fg_i(sn_i^*, sg_i^*, xg_{inter,i}^*) = -\beta^I sg_i^* \\ \qquad + \alpha^I T_{gaba}(1 - sg_i^*) \dfrac{a_I(J_{nmda} sn_i^* - J_- sg_i^* + xg_{inter,i}^*) - b_I}{1 - e^{-d_I[a_I(J_{nmda} sn_i^* - J_- sg_i^* + xg_{inter,i}^*) - b_I]}} \end{cases} \tag{10}$$

and where $\overrightarrow{x_{inter}}$, given by Eq 4, will be the transfer variables.

1.3 Expressing sensitivities w.r.t. forcings

1.3.1 Perturbation of one forcing B

Here, we build the general expression of the sensitivity, for any given forcing B among B_{E_1}, B_{E_2}, B_{E_3}, B_{I_1}, B_{I_2} or B_{I_3}.

The perturbed form of system 9 reads:

$$\begin{cases} \overrightarrow{F}(\overrightarrow{s}, \overrightarrow{x_{inter}}) = \overrightarrow{0} \\ \overrightarrow{x_{inter}} = \overrightarrow{w_{inter}}(\overrightarrow{s}, B + \delta B) \end{cases} \tag{11}$$

By linearization at fixed point, we get:

$$\begin{cases} \overline{\overline{\dfrac{\partial F}{\partial s}}} \overrightarrow{\delta s_B} + \overline{\overline{\dfrac{\partial F}{\partial x_{inter}}}} \overrightarrow{\delta x_{inter B}} = \overrightarrow{0} \\ \overrightarrow{\delta x_{inter B}} = \overline{\overline{\dfrac{\partial w_{inter}}{\partial s}}} \overrightarrow{\delta s_B} + \overrightarrow{\dfrac{\partial w_{inter}}{\partial B}} \delta B \end{cases} \tag{12}$$

Plugging the second equation into the first, we have:

$$(\overline{\overline{\frac{\partial F}{\partial s}}} + \overline{\overline{\frac{\partial F}{\partial x_{inter}} \frac{\partial w_{inter}}{\partial s}}})\overrightarrow{\delta s_B} = -\overline{\overline{\frac{\partial F}{\partial x_{inter}}}}\overrightarrow{\frac{\partial w_{inter}}{\partial B}}\delta B \tag{13}$$

Denoting

$$\overline{\overline{S}} = -\overline{\overline{\frac{\partial F}{\partial s}}}^{-1}\overline{\overline{\frac{\partial F}{\partial x_{inter}}}} \tag{14}$$

expression 13 can be written under the canonical form as:

$$(\overline{\overline{\mathbb{I}}} - \overline{\overline{S}}\,\overline{\overline{\frac{\partial w_{inter}}{\partial s}}})\overrightarrow{\delta s_B} = \overline{\overline{S}}\,\overrightarrow{\frac{\partial w_{inter}}{\partial B}}\delta B \tag{15}$$

In this expression $\overline{\overline{\frac{\partial w_{inter}}{\partial s}}}$ represents the coupling between the three areas, and is:

$$\overline{\overline{\frac{\partial w_{inter}}{\partial s}}} = \begin{pmatrix} 0 & \frac{\partial wn_{inter,1}}{\partial sn_2} & \frac{\partial wn_{inter,1}}{\partial sn_3} & 0 & 0 & 0 \\ \frac{\partial wn_{inter,2}}{\partial sn_1} & 0 & \frac{\partial wn_{inter,2}}{\partial sn_3} & 0 & 0 & 0 \\ \frac{\partial wn_{inter,3}}{\partial sn_1} & \frac{\partial wn_{inter,2}}{\partial sn_2} & 0 & 0 & 0 & 0 \\ 0 & \frac{\partial wg_{inter,1}}{\partial sn_2} & \frac{\partial wg_{inter,1}}{\partial sn_3} & 0 & 0 & 0 \\ \frac{\partial wg_{inter,2}}{\partial sn_1} & 0 & \frac{\partial wg_{inter,2}}{\partial sn_3} & 0 & 0 & 0 \\ \frac{\partial wg_{inter,3}}{\partial sn_1} & \frac{\partial wg_{inter,3}}{\partial sn_2} & 0 & 0 & 0 & 0 \end{pmatrix} \tag{16}$$

$$= \begin{pmatrix} 0 & k_{E_{12}}\kappa_{12} & k_{E_{13}}\kappa_{13} & 0 & 0 & 0 \\ k_{E_{21}}\kappa_{21} & 0 & k_{E_{23}}\kappa_{23} & 0 & 0 & 0 \\ k_{E_{31}}\kappa_{31} & k_{E_{32}}\kappa_{32} & 0 & 0 & 0 & 0 \\ 0 & (1-k_{E_{12}})\kappa_{12} & (1-k_{E_{13}})\kappa_{13} & 0 & 0 & 0 \\ (1-k_{E_{21}})\kappa_{21} & 0 & (1-k_{E_{23}})\kappa_{23} & 0 & 0 & 0 \\ (1-k_{E_{31}})\kappa_{31} & (1-k_{E_{32}})\kappa_{32} & 0 & 0 & 0 & 0 \end{pmatrix} \tag{17}$$

If we set $\overline{\overline{\frac{\partial w_{inter}}{\partial s}}} = \overline{\overline{0}}$ while the r.h.s. remains untouched, we then obtain the effect of perturbation in the open loop case:

$$\overrightarrow{\delta s}_B^O = \overline{\overline{S}}\overrightarrow{\frac{\partial w_{inter}}{\partial B}}\delta B \tag{18}$$

The *legible* expression then reads:

$$\boxed{(\overline{\overline{\mathbb{I}}} - \overline{\overline{G}})\overrightarrow{\delta s}_B = \overrightarrow{\delta s}_B^O} \tag{19}$$

where

$$\overline{\overline{G}} = -\overline{\overline{\frac{\partial F}{\partial s}}}^{-1}\overline{\overline{\frac{\partial F}{\partial x_{inter}}\frac{\partial w_{inter}}{\partial s}}} = \overline{\overline{S}}\overline{\overline{\frac{\partial w_{inter}}{\partial s}}} \tag{20}$$

is the feedback gain matrix.

1.3.2 Expressing Open Loop Sensitivities

Perturbations are operated upon transfer variables, given by $\overrightarrow{w_{inter}}$.

Let $\overrightarrow{x_{inter}}^O$ denote the transfer variables in open loop condition when $\overline{\overline{\frac{\partial w_{inter}}{\partial s}}} = \overline{\overline{0}}$ (i.e. $\kappa_{12} = \kappa_{21} = \kappa_{13} = \kappa_{31} = \kappa_{23} = \kappa_{32} = 0$):

$$\overrightarrow{x_{inter}}^O = \begin{pmatrix} B_{E_1} \\ B_{E_2} \\ B_{E_3} \\ B_{I_1} \\ B_{I_2} \\ B_{I_3} \end{pmatrix} \tag{21}$$

Since forcing parameters are independent, we have: $\overline{\overline{\frac{\partial w_{inter}}{\partial x^O_{inter}}}} = \overline{\overline{\mathbb{I}}}$.

Hence considering each perturbation one by one, the operator $\overrightarrow{\frac{\partial w_{inter}}{\partial B}}$ acts as a selector of a column of $\overline{\overline{S}}$, so that we can express the sensitivities in closed loop condition as function of sensitivities in open loop condition.

For instance, perturbing B_{E_1}, we get:

$$\overrightarrow{\delta s}^{O}_{B_{E_1}} = \overline{\overline{S}} \overrightarrow{\frac{\partial w_{inter}}{\partial B_{E_1}}} \delta B_{E_1} \Longleftrightarrow \frac{\overrightarrow{\delta s}^{O}_{B_{E_1}}}{\delta B_{E_1}} = \overline{\overline{S}} \underbrace{\overrightarrow{\frac{\partial w_{inter}}{\partial B_{E_1}}}}_{\begin{bmatrix} 1 \\ 0 \\ 0 \\ 0 \\ 0 \\ 0 \end{bmatrix}} \tag{22}$$

hence the open loop sensitivities of both areas to a perturbation upon the excitatory pool of the first area yields the first column of $\overline{\overline{S}}$.

Obviously, in the open loop condition, the second and third areas are not perturbed at all.

Furthermore, considering that perturbing here B_{E_1} is the same as perturbing x_{E_1} in the first area considered as an isolated area, we have, by definition given in Eq **??**, that:

$$\overrightarrow{\delta s}^{O}_{B_{E_1}} = \begin{pmatrix} \delta sn_1 \\ \delta sn_2 \\ \delta sn_3 \\ \delta sg_1 \\ \delta sg_2 \\ \delta sg_3 \end{pmatrix}^{O}_{B_{E_1}} = \begin{pmatrix} \mathscr{A}_{sn_1, x_{E_1}} \delta B_{E_1} \\ 0 \\ 0 \\ \mathscr{A}_{sg_1, x_{E_1}} \delta B_{E_1} \\ 0 \\ 0 \end{pmatrix} \tag{23}$$

hence the first column of $\overline{\overline{S}}$ is given by:

$$\frac{\overrightarrow{\delta s}^{O}_{B_{E_1}}}{\delta B_{E_1}} = \begin{pmatrix} \mathscr{A}_{sn_1,x_{E_1}} \\ 0 \\ 0 \\ \mathscr{A}_{sg_1,x_{E_1}} \\ 0 \\ 0 \end{pmatrix} \tag{24}$$

Following the same lines of reasoning for the five other perturbations, we finally obtain:

$$\overline{\overline{S}} = \begin{pmatrix} \overline{\overline{\mathscr{A}_{sn,x_E}}} & \overline{\overline{\mathscr{A}_{sn,x_I}}} \\ \overline{\overline{\mathscr{A}_{sg,x_E}}} & \overline{\overline{\mathscr{A}_{sg,x_I}}} \end{pmatrix} \tag{25}$$

with

$$\overline{\overline{\mathscr{A}_{sn,x_E}}} = \begin{pmatrix} \mathscr{A}_{sn_1,x_{E_1}} & 0 & 0 \\ 0 & \mathscr{A}_{sn_2,x_{E_2}} & 0 \\ 0 & 0 & \mathscr{A}_{sn_3,x_{E_3}} \end{pmatrix}, \quad \overline{\overline{\mathscr{A}_{sn,x_I}}} = \begin{pmatrix} \mathscr{A}_{sn_1,x_{I_1}} & 0 & 0 \\ 0 & \mathscr{A}_{sn_2,x_{I_2}} & 0 \\ 0 & 0 & \mathscr{A}_{sn_3,x_{I_3}} \end{pmatrix} \tag{26}$$

$$\overline{\overline{\mathscr{A}_{sg,x_E}}} = \begin{pmatrix} \mathscr{A}_{sg_1,x_{E_1}} & 0 & 0 \\ 0 & \mathscr{A}_{sg_2,x_{E_2}} & 0 \\ 0 & 0 & \mathscr{A}_{sg_3,x_{E_3}} \end{pmatrix}, \quad \overline{\overline{\mathscr{A}_{sg,x_I}}} = \begin{pmatrix} \mathscr{A}_{sg_1,x_{I_1}} & 0 & 0 \\ 0 & \mathscr{A}_{sg_2,x_{I_2}} & 0 \\ 0 & 0 & \mathscr{A}_{sg_3,x_{I_3}} \end{pmatrix} \tag{27}$$

1.4 Using Isolated Area Sensitivities

In the same spirit as in Sec **??**, these open loop sensitivities of areas can be expressed by the analytical expression of their sensitivities when considered isolated, yet to be evaluated respectively at fixed points $\overrightarrow{s_1}^*$, $\overrightarrow{s_2}^*$ and $\overrightarrow{s_3}^*$ yielded by the closed loop system and taking into account the total amount of external forcing, so we write:

$$\begin{cases} \mathscr{A}_{sn_i,x_{E_i}} = \Phi n_{_E}(\overrightarrow{s^*} = \overrightarrow{s_i^*}, x_E = xn^*_{inter,i}, x_I = xg^*_{inter,i}) \\ \mathscr{A}_{sg_i,x_{E_i}} = \Phi g_{_E}(\overrightarrow{s^*} = \overrightarrow{s_i^*}, x_E = xn^*_{inter,i}, x_I = xg^*_{inter,i}) \\ \mathscr{A}_{sn_i,x_{I_i}} = \Phi n_{_I}(\overrightarrow{s^*} = \overrightarrow{s_i^*}, x_E = xn^*_{inter,i}, x_I = xg^*_{inter,i}) \\ \mathscr{A}_{sg_i,x_{I_i}} = \Phi g_{_I}(\overrightarrow{s^*} = \overrightarrow{s_i^*}, x_E = xn^*_{inter,i}, x_I = xg^*_{inter,i}) \end{cases} \tag{28}$$

where $\overrightarrow{s_i^*} = (sn_i^*, sg_i^*)$ and $xn^*_{inter,i}$, $x_I = xg^*_{inter,i}$ are defined in Eq 4. Analytical expression for the functions $\Phi n_{_E}$, $\Phi g_{_E}$, $\Phi n_{_I}$ and $\Phi g_{_I}$ are explicitly given in Sec **??**.

Closed Loop Sensitivities as functions of Open Loop Sensitivities

From definition 20 for $\overline{\overline{G}}$, we have:

$$\overline{\overline{G}} = \begin{pmatrix} \mathscr{A}_{sn_1,x_{E_1}} & 0 & 0 & \mathscr{A}_{sn_1,x_{I_1}} & 0 & 0 \\ 0 & \mathscr{A}_{sn_2,x_{E_2}} & 0 & 0 & \mathscr{A}_{sn_2,x_{I_2}} & 0 \\ 0 & 0 & \mathscr{A}_{sn_3,x_{E_3}} & 0 & 0 & \mathscr{A}_{sn_3,x_{I_3}} \\ \mathscr{A}_{sg_1,x_{E_1}} & 0 & 0 & \mathscr{A}_{sg_1,x_{I_1}} & 0 & 0 \\ 0 & \mathscr{A}_{sg_2,x_{E_2}} & 0 & 0 & \mathscr{A}_{sg_2,x_{I_2}} & 0 \\ 0 & 0 & \mathscr{A}_{sg_3,x_{E_3}} & 0 & 0 & \mathscr{A}_{sg_3,x_{I_3}} \end{pmatrix} \tag{29}$$

$$\times \begin{pmatrix} 0 & k_{E_{12}}\kappa_{12} & k_{E_{13}}\kappa_{13} & 0 & 0 & 0 \\ k_{E_{21}}\kappa_{21} & 0 & k_{E_{23}}\kappa_{23} & 0 & 0 & 0 \\ k_{E_{31}}\kappa_{31} & k_{E_{32}}\kappa_{32} & 0 & 0 & 0 & 0 \\ 0 & (1-k_{E_{12}})\kappa_{12} & (1-k_{E_{13}})\kappa_{13} & 0 & 0 & 0 \\ (1-k_{E_{21}})\kappa_{21} & 0 & (1-k_{E_{23}})\kappa_{23} & 0 & 0 & 0 \\ (1-k_{E_{31}})\kappa_{31} & (1-k_{E_{32}})\kappa_{32} & 0 & 0 & 0 & 0 \end{pmatrix} \tag{30}$$

that we will write as:

$$\overline{\overline{G}} = \begin{bmatrix} 0 & G_{12} & G_{13} & 0 & 0 & 0 \\ G_{21} & 0 & G_{23} & 0 & 0 & 0 \\ G_{31} & G_{32} & 0 & 0 & 0 & 0 \\ 0 & G_{42} & G_{43} & 0 & 0 & 0 \\ G_{51} & 0 & G_{53} & 0 & 0 & 0 \\ G_{61} & G_{62} & 0 & 0 & 0 & 0 \end{bmatrix} \tag{31}$$

with

$$\begin{cases} G_{12} = \mathscr{A}_{sn_1,x_{E_1}} k_{E_{12}} \kappa_{12} + \mathscr{A}_{sn_1,x_{I_1}} (1 - k_{E_{12}}) \kappa_{12} \\ G_{13} = \mathscr{A}_{sn_1,x_{E_1}} k_{E_{13}} \kappa_{13} + \mathscr{A}_{sn_1,x_{I_1}} (1 - k_{E_{13}}) \kappa_{13} \\ G_{21} = \mathscr{A}_{sn_2,x_{E_2}} k_{E_{21}} \kappa_{21} + \mathscr{A}_{sn_2,x_{I_2}} (1 - k_{E_{21}}) \kappa_{21} \\ G_{23} = \mathscr{A}_{sn_2,x_{E_2}} k_{E_{23}} \kappa_{23} + \mathscr{A}_{sn_2,x_{I_2}} (1 - k_{E_{23}}) \kappa_{23} \\ G_{31} = \mathscr{A}_{sn_3,x_{E_3}} k_{E_{31}} \kappa_{31} + \mathscr{A}_{sn_3,x_{I_3}} (1 - k_{E_{31}}) \kappa_{31} \\ G_{32} = \mathscr{A}_{sn_3,x_{E_3}} k_{E_{32}} \kappa_{32} + \mathscr{A}_{sn_3,x_{I_3}} (1 - k_{E_{32}}) \kappa_{32} \\ G_{42} = \mathscr{A}_{sg_1,x_{E_1}} k_{E_{12}} \kappa_{12} + \mathscr{A}_{sg_1,x_{I_1}} (1 - k_{E_{12}}) \kappa_{12} \\ G_{43} = \mathscr{A}_{sg_1,x_{E_1}} k_{E_{13}} \kappa_{13} + \mathscr{A}_{sg_1,x_{I_1}} (1 - k_{E_{13}}) \kappa_{13} \\ G_{51} = \mathscr{A}_{sg_2,x_{E_2}} k_{E_{21}} \kappa_{21} + \mathscr{A}_{sg_2,x_{I_2}} (1 - k_{E_{21}}) \kappa_{21} \\ G_{53} = \mathscr{A}_{sg_2,x_{E_2}} k_{E_{23}} \kappa_{23} + \mathscr{A}_{sg_2,x_{I_2}} (1 - k_{E_{23}}) \kappa_{23} \\ G_{61} = \mathscr{A}_{sg_3,x_{E_3}} k_{E_{31}} \kappa_{31} + \mathscr{A}_{sg_3,x_{I_3}} (1 - k_{E_{31}}) \kappa_{31} \\ G_{62} = \mathscr{A}_{sg_3,x_{E_3}} k_{E_{32}} \kappa_{32} + \mathscr{A}_{sg_3,x_{I_3}} (1 - k_{E_{32}}) \kappa_{32} \end{cases} \tag{32}$$

For the l.h.s. term in expression 19, we then have:

$$\overline{\overline{\mathbb{I}}} - \overline{\overline{G}} = \left(\begin{array}{ccc|ccc} 1 & -G_{12} & -G_{13} & 0 & 0 & 0 \\ -G_{21} & 1 & -G_{23} & 0 & 0 & 0 \\ -G_{31} & -G_{32} & 1 & 0 & 0 & 0 \\ \hline 0 & -G_{42} & -G_{43} & 1 & 0 & 0 \\ -G_{51} & 0 & -G_{53} & 0 & 1 & 0 \\ -G_{61} & -G_{62} & 0 & 0 & 0 & 1 \end{array}\right) \tag{33}$$

that we will write as:

$$\overline{\overline{\mathbb{I}}} - \overline{\overline{G}} = \left(\begin{array}{c|c} \overline{\overline{B_1}} & \overline{\overline{B_2}} \\ \hline \overline{\overline{B_3}} & \overline{\overline{B_4}} \end{array}\right) \tag{34}$$

Considering the property that

$$\text{If } \overline{\overline{M}} = \begin{pmatrix} \overline{\overline{A}} & \overline{\overline{B}} \\ \overline{\overline{C}} & \overline{\overline{D}} \end{pmatrix} \text{ with } \overline{\overline{D}} \text{ invertible}$$

$$\text{then } \overline{\overline{M}}^{-1} = \begin{pmatrix} \overline{\overline{R}} & \overline{\overline{S}} \\ \overline{\overline{T}} & \overline{\overline{U}} \end{pmatrix} \text{ with } \begin{cases} \overline{\overline{R}} = \left(\overline{\overline{A}} - \overline{\overline{BD}}^{-1}\overline{\overline{C}}\right)^{-1} \\ \overline{\overline{S}} = -\overline{\overline{RBD}}^{-1} \\ \overline{\overline{T}} = -\overline{\overline{D}}^{-1}\overline{\overline{CR}} \\ \overline{\overline{U}} = \overline{\overline{D}}^{-1}\left(\overline{\overline{\mathbb{I}}} - \overline{\overline{CS}}\right) \end{cases}$$

we obtain

$$\left(\bar{\bar{\mathbb{I}}} - \overline{\overline{G}}\right)^{-1} = \left(\begin{array}{c|c} \overline{\overline{C_1}} & \overline{\overline{C_2}} \\ \hline \overline{\overline{C_3}} & \overline{\overline{C_4}} \end{array}\right) \text{ where } \begin{cases} \overline{\overline{C_1}} = \left(\overline{\overline{B_1}} - \overline{\overline{B_2}}(\overline{\overline{B_4}}^{-1})\overline{\overline{B_3}}\right)^{-1} = \overline{\overline{B_1}}^{-1} \\ \overline{\overline{C_2}} = -\overline{\overline{C_1 B_2}}(\overline{\overline{B_4}}^{-1}) = \bar{\bar{0}} \\ \overline{\overline{C_3}} = -\overline{\overline{B_4}}^{-1}\overline{\overline{B_3 C_1}} = -\overline{\overline{B_3 C_1}} = -\overline{\overline{B_3 B_1}}^{-1} \\ \overline{\overline{C_4}} = \overline{\overline{B_4}}^{-1}\left(\bar{\bar{\mathbb{I}}} - \overline{\overline{B_3 C_2}}\right) = \bar{\bar{\mathbb{I}}} \end{cases} \tag{35}$$

$$= \left(\begin{array}{c|c} \overline{\overline{B_1}}^{-1} & \bar{\bar{0}} \\ \hline -\overline{\overline{B_3 B_1}}^{-1} & \bar{\bar{\mathbb{I}}} \end{array}\right) \tag{36}$$

To recover sensitivities for the closed loop condition, we then consider:

$$(\bar{\bar{\mathbb{I}}} - \overline{\overline{G}})^{-1}\overline{\overline{S}} = \left(\begin{array}{c|c} \overline{\overline{B_1}}^{-1} & \bar{\bar{0}} \\ \hline -\overline{\overline{B_3 B_1}}^{-1} & \bar{\bar{\mathbb{I}}} \end{array}\right) \left(\begin{array}{c|c} \overline{\overline{\mathscr{A}_{sn,x_E}}} & \overline{\overline{\mathscr{A}_{sn,x_I}}} \\ \hline \overline{\overline{\mathscr{A}_{sg,x_E}}} & \overline{\overline{\mathscr{A}_{sg,x_I}}} \end{array}\right) \tag{37}$$

$$= \left(\begin{array}{c|c} \overline{\overline{B_1}}^{-1}\overline{\overline{\mathscr{A}_{sn,x_E}}} & \overline{\overline{B_1}}^{-1}\overline{\overline{\mathscr{A}_{sn,x_I}}} \\ \hline -\overline{\overline{B_3 B_1}}^{-1}\overline{\overline{\mathscr{A}_{sn,x_E}}} + \overline{\overline{\mathscr{A}_{sg,x_E}}} & -\overline{\overline{B_3 B_1}}^{-1}\overline{\overline{\mathscr{A}_{sn,x_I}}} + \overline{\overline{\mathscr{A}_{sg,x_I}}} \end{array}\right) \tag{38}$$

so that

$$\boxed{\frac{\overrightarrow{\delta s_B}}{\delta B} = (\bar{\bar{\mathbb{I}}} - \overline{\overline{G}})^{-1}\overline{\overline{S}}\frac{\overrightarrow{\partial w_{inter}}}{\partial B}} \tag{39}$$

expresses, in full generality for the three-area system, the matrix of sensitivities to a perturbation upon either forcing B. They are expressed as functions of the sensitivities in single-area system, which are in turn expressed as functions of the sensitivities in the single-pool system.

1.5 Sensitivities in a Control-Relay-Target system

We now focus on the question of how sensitivities would drive the response of the excitatory pool of one area to the activation of the excitatory pool of the other one, depending on the connectivity between the three areas. Hence, we attribute a role to each area: the area 1 which excitatory pool is positively perturbed will be called "Control" area (denoted by C), the area 2 will be called "Target area" (denoted by T) and the area 3 will be called "Relay area" (denoted by R).

From now on, the observable will then be denoted as:

$$\vec{s} = \begin{pmatrix} sn_C \\ sn_T \\ sn_R \\ sg_C \\ sg_T \\ sg_R \end{pmatrix} \tag{40}$$

and the transfer variables as:

$$\vec{x} = \begin{pmatrix} xn_{intra,C} & = & B_{E_C} & + & k_{E_{CT}} \kappa_{CT} sn_T + & k_{E_{CR}} \kappa_{CR} sn_R \\ xn_{intra,T} & = & B_{E_T} & + & k_{E_{TC}} \kappa_{TC} sn_C + & k_{E_{TR}} \kappa_{TR} sn_R \\ xn_{intra,R} & = & B_{E_R} & + & k_{E_{RC}} \kappa_{RC} sn_C + & k_{E_{RT}} \kappa_{RT} sn_T \\ xg_{intra,C} & = & B_{I_C} & + & (1 - k_{E_{CT}}) \kappa_{CT} sn_T + & (1 - k_{E_{CR}}) \kappa_{CR} sn_R \\ xg_{intra,T} & = & B_{I_T} & + & (1 - k_{E_{TC}}) \kappa_{TC} sn_C + & (1 - k_{E_{TR}}) \kappa_{TR} sn_R \\ xg_{intra,R} & = & B_{I_R} & + & (1 - k_{E_{RC}}) \kappa_{RC} sn_C + & (1 - k_{E_{RT}}) \kappa_{RT} sn_T \end{pmatrix} \tag{41}$$

and we focus on:

$$\begin{pmatrix} \delta sn_T \\ \delta sn_C \end{pmatrix} \tag{42}$$

in response to δB_{E_C}.

To extract the situation of interest, from the general result above, we then pick the case:

$$\overrightarrow{\delta s}_{B_{E_C}} = \left(\bar{\bar{\mathbb{I}}} - \overline{\overline{G}}\right)^{-1} \overline{\overline{S}} \overrightarrow{\frac{\partial w_{inter}}{\partial B_{E_C}}} \delta B_{E_C} \tag{43}$$

$$= \left(\begin{array}{c|c} \overline{\overline{B_1}}^{-1} \overline{\overline{\mathscr{A}_{sn,x_E}}} & \overline{\overline{B_1}}^{-1} \overline{\overline{\mathscr{A}_{sn,x_I}}} \\ \hline -\overline{\overline{B_3 B_1}}^{-1} \overline{\overline{\mathscr{A}_{sn,x_E}}} + \overline{\overline{\mathscr{A}_{sg,x_E}}} & -\overline{\overline{B_3 B_1}}^{-1} \overline{\overline{\mathscr{A}_{sn,x_I}}} + \overline{\overline{\mathscr{A}_{sg,x_I}}} \end{array} \right) \left(\begin{array}{c} 1 \\ 0 \\ 0 \\ \hline 0 \\ 0 \\ 0 \end{array} \right) \delta B_{E_C} \tag{44}$$

$$= \left(\begin{array}{c} \overline{\overline{B_1}}^{-1} \overline{\overline{\mathscr{A}_{sn,x_E}}} \begin{pmatrix} 1 \\ 0 \\ 0 \end{pmatrix} \\ \hline \left(-\overline{\overline{B_3 B_1}}^{-1} \overline{\overline{\mathscr{A}_{sn,x_E}}} + \overline{\overline{\mathscr{A}_{sg,x_E}}} \right) \begin{pmatrix} 1 \\ 0 \\ 0 \end{pmatrix} \end{array} \right) \delta B_{E_C} \tag{45}$$

$$\equiv \left(\begin{array}{c} \delta sn_C \\ \delta sn_T \\ \delta sn_R \\ \hline \delta sg_C \\ \delta sg_T \\ \delta sg_R \end{array} \right) \tag{46}$$

where the perturbations of interest are in the upper part, and we have:

$$\begin{pmatrix} \delta sn_C \\ \delta sn_T \\ \delta sn_R \end{pmatrix} = \overline{\overline{B_1}}^{-1} \overline{\overline{\mathscr{A}_{sn,x_E}}} \begin{pmatrix} 1 \\ 0 \\ 0 \end{pmatrix} \delta B_{E_C} \tag{47}$$

From definition 34, we get

$$\overline{\overline{B_1}}^{-1} = \begin{pmatrix} 1 & -G_{12} & -G_{13} \\ -G_{21} & 1 & -G_{23} \\ -G_{31} & -G_{32} & 1 \end{pmatrix}^{-1} \tag{48}$$

$$= \frac{1}{1 - G_{23}G_{32} - G_{12}(G_{21} + G_{23}G_{31}) - G_{13}(G_{21}G_{32} + G_{31})} \tag{49}$$

$$\begin{pmatrix} 1 - G_{23}G_{32} & G_{12} + G_{13}G_{32} & G_{12}G_{23} + G_{13} \\ G_{21} + G_{23}G_{31} & 1 - G_{13}G_{31} & G_{23} + G_{13}G_{21}) \\ G_{21}G_{32} + G_{31} & G_{32} + G_{12}G_{31} & 1 - G_{12}G_{21} \end{pmatrix} \tag{50}$$

hence

$$\begin{pmatrix} \delta sn_C \\ \delta sn_T \\ \delta sn_R \end{pmatrix} = \frac{1}{1 - G_{23}G_{32} - G_{12}(G_{21} + G_{23}G_{31}) - G_{13}(G_{21}G_{32} + G_{31})}$$

$$\begin{pmatrix} 1 - G_{23}G_{32} & G_{12} + G_{13}G_{32} & G_{12}G_{23} + G_{13} \\ G_{21} + G_{23}G_{31} & 1 - G_{13}G_{31} & G_{23} + G_{13}G_{21} \\ G_{21}G_{32} + G_{31} & G_{32} + G_{12}G_{31} & 1 - G_{12}G_{21} \end{pmatrix} \begin{pmatrix} \mathscr{A}_{sn_C, x_{E_C}} & 0 & 0 \\ 0 & \mathscr{A}_{sn_T, x_{E_T}} & 0 \\ 0 & 0 & \mathscr{A}_{sn_R, x_{E_R}} \end{pmatrix} \begin{pmatrix} 1 \\ 0 \\ 0 \end{pmatrix} \delta B_{E_C} \tag{51}$$

$$= \frac{1}{1 - G_{23}G_{32} - G_{12}(G_{21} + G_{23}G_{31}) - G_{13}(G_{21}G_{32} + G_{31})}$$

$$\begin{pmatrix} (1 - G_{23}G_{32})\mathscr{A}_{sn_C, x_{E_C}} & G_{12} + G_{13}G_{32}\mathscr{A}_{sn_T, x_{E_T}} & (G_{12}G_{23} + G_{13})\mathscr{A}_{sn_R, x_{E_R}} \\ (G_{21} + G_{23}G_{31})\mathscr{A}_{sn_C, x_{E_C}} & (1 - G_{13}G_{31})\mathscr{A}_{sn_T, x_{E_T}} & (G_{23} + G_{13}G_{21})\mathscr{A}_{sn_R, x_{E_R}} \\ (G_{21}G_{32} + G_{31})\mathscr{A}_{sn_C, x_{E_C}} & (G_{32} + G_{12}G_{31})\mathscr{A}_{sn_T, x_{E_T}} & (1 - G_{12}G_{21})\mathscr{A}_{sn_R, x_{E_R}} \end{pmatrix} \begin{pmatrix} 1 \\ 0 \\ 0 \end{pmatrix} \delta B_{E_C} \tag{52}$$

$$= \frac{1}{1 - G_{23}G_{32} - G_{12}(G_{21} + G_{23}G_{31}) - G_{13}(G_{21}G_{32} + G_{31})} \begin{pmatrix} (1 - G_{23}G_{32})\mathscr{A}_{sn_C, x_{E_C}} \\ (G_{21} + G_{23}G_{31})\mathscr{A}_{sn_C, x_{E_C}} \\ (G_{21}G_{32} + G_{31})\mathscr{A}_{sn_C, x_{E_C}} \end{pmatrix} \delta B_{E_C} \tag{53}$$

with

$$
\begin{cases}
G_{12} = \mathscr{A}_{sn_C, x_{E_C}} k_{E_{CT}} \kappa_{CT} + \mathscr{A}_{sn_C, x_{I_C}} (1 - k_{E_{CT}}) \kappa_{CT} \\
G_{13} = \mathscr{A}_{sn_C, x_{E_C}} k_{E_{CR}} \kappa_{CR} + \mathscr{A}_{sn_C, x_{I_C}} (1 - k_{E_{CR}}) \kappa_{CR} \\
G_{21} = \mathscr{A}_{sn_T, x_{E_T}} k_{E_{TC}} \kappa_{TC} + \mathscr{A}_{sn_T, x_{I_T}} (1 - k_{E_{TC}}) \kappa_{TC} \\
G_{23} = \mathscr{A}_{sn_T, x_{E_T}} k_{E_{TR}} \kappa_{TR} + \mathscr{A}_{sn_T, x_{I_T}} (1 - k_{E_{TR}}) \kappa_{TR} \\
G_{31} = \mathscr{A}_{sn_R, x_{E_R}} k_{E_{RC}} \kappa_{RC} + \mathscr{A}_{sn_R, x_{I_R}} (1 - k_{E_{RC}}) \kappa_{RC} \\
G_{32} = \mathscr{A}_{sn_R, x_{E_R}} k_{E_{RT}} \kappa_{RT} + \mathscr{A}_{sn_R, x_{I_R}} (1 - k_{E_{RT}}) \kappa_{RT}
\end{cases}
\tag{54}
$$
