# Supplementary material for: Sensitivity analysis enlightens effects of connectivity in a Neural Mass Model under Control-Target mode
Source: PLoS Comput Biol. 2026 Mar 23;22(3):e1014035. doi: 10.1371/journal.pcbi.1014035 (PMC13008111; doi:10.1371/journal.pcbi.1014035)
Supplement: Attachment — Submitted filename: ValletEtAl-R2R.pdf [file pcbi.1014035.s006.pdf]

# Response to Reviewers

## Sensitivity analysis enlightens effects of connectivity in a Neural Mass Model under Control-Target mode

Vallet Anaïs[1], Blanco Stéphane[2], Chevallier Coline[2,3], Eustache Francis[1], Gautrais Jacques[2,3,*], Grandpeix Jean-Yves[4], Joly Jean-Louis[2], Segobin Shailendra[1], Gagnepain Pierre[1]

**1** Normandie Univ, UNICAEN, PSL Research University, EPHE, INSERM, U1077, CHU de Caen, GIP Cyceron, Neuropsychologie et Imagerie de la Mémoire Humaine, 14000 Caen, France

**2** LAPLACE, Université de Toulouse, CNRS, INPT, UPS, Toulouse, France

**3** Centre de Recherches sur la Cognition Animale (CRCA), Centre de Biologie Intégrative (CBI), Université de Toulouse, CNRS, UPS, France

**4** LMD/IPSL, Sorbonne Université, CNRS, École Polytechnique, ENS, Paris, France

* jacques.gautrais@cnrs.fr

To the editor and reviewers :

Thank you for your time expertising our work. Your input has allowed us to improve on our manuscript and its presentation to the readership of PCBi. We have addressed your queries to the best of our abilities and understanding, and we hope to have fulfilled your expectations.

# 1 To the editor

We have a concern about the response by Reviewer 2 on topic : "Have the authors made all data and (if applicable) computational code underlying the findings in their manuscript fully available?" to which Rev2 answers : No: i could not find any git repo linked in the paper.

With regards to 'computational' code, one can consider that all information needed to reproduce the results are indeed made available in the text and SI, since there are no specific nor hidden aspects with numerical implementation for solving the equations we provide: one has just to translate the given equations into a code using the coding language of its choice. As for ourselves, we partly have used python, partly have used C/Fortran based own codes, within our own numerical ecosystem. We certainly do not plan to publish this full ecosystem on any private web-based git platforms (like github/gitlab) as we rely upon our own scholar ssh-only git architecture (disconnected from the web), and even if we did so, a "user" would so need to configure and tune its linux system in order to be able to use it that only some container-based solution would be of use. This is not within our area of expertise. However, at the editor's request and subject to acceptance of the article, we would fully comply with a request to extract only the Python code sections and translate the C/For code into Python in order to submit a standalone Python code as an independent SI.py, which would reproduce the data used in the figures. In terms of availability/reproducibility, this would only duplicate our equations and would only be useful for Python programmers, but we can understand why the editor would request such a numerical implementation archive. Would it be so, we plan an E.T.A. of about two weeks.

Note : line numbers below refer to the revised version (and have been edited in reviewers' responses accordingly).

# 2 Reviewer 1

## 2.1 General comments

Reviewer 1: Thank you of this paper. It discusses a technical but important aspect of understanding the excitatory inhibitory balance of the brain. The analysis is sound and correct. The derivations are easy to follow. There a few comments on the paper.

## 2.2 Major comments

The paper studied how the stationary points of a dynamic system with single and collections of neuronal pools change depending on the internal connectivity and external forcing. The stability of a stationary point was partly explained for the single state system (excitation or inhibition only) but I did not see a stability analysis for the interconnected neural pools. This paper would gain considerably with an analytical section on the stability of the stationary points for each different connectivity set-up.

*We fully agree. An additional section has been added at the end of the SI 1 presenting the requested linear stability analysis. It contains the formal developments we used to construct it from sensitivities expression by leveraging our nested levels methodology. Stability of fixed points has then been checked numerically. The real part of eigenvalues are all negative, so the fixed points presented in the article are stable in all cases.*

## 2.3 Minor comments

1. The notation is not useful for a reader not accustomed to the specific references noted in the paper. The use of e.g. sn and sg for the state of the NMDA or GABA looks more like

a product between s and n or g. It would be useful to use a specific notation for the terms (greek letters or other specific math symbols).

*We completely agree with this point, and in fact, it was discussed to some extent within our own group at the beginning of the work. We ultimately decided to retain the notation adopted by the original community, as they can more easily understand our work if it is expressed in their own notations, the use of which is linked to the meaning that motivates them (sn for synaptic NMDA and sg for synaptic GABA). We do not feel entitled to completely change the notations on our own initiative, especially with regard to the state variables.*

*For readers more accustomed to formally cleaner symbols, the conventions are explicitly given at lines 104/105, 108 and 110.*

2. In equation 1 is $\sigma v_i$ a random noise? and if so how was it dealt with in deriving equation 5. Are there underlying assumptions of stability of the system? Again a stability analysis would be useful.

   $\sigma v_i$ *in Eq 1 is indeed a random noise in the original model. Given the linear stability of fixed points, this term plays no role in the system response (in our range of parameters), and has been discarded to develop the analytical work.*

3. Line 209: What is canonical with equation 10. It is a useful expression, and used repeatedly in the manuscript but I do not see why it is called canonical.

   *We understand the point raised by the reviewer, as we use this term internally, which comes from the feedback vocabulary in signal theory. We replaced it with "legible", which conveys just as well the central role this expression plays in our context, and which is less likely to raise questions...*

4. Line (previously below eq (10)): What does this mean: ...non linearity is the self-amplification of the excitatory pool by the recurrent connectivity, which is expressed in the function wn through the parameter W+.

   *We understand from this comment that the sentence was not placed in the right place. It has*

*been expanded and moved after expression (5), lines 193-200, and serves here to point out when and how the system is nonlinear (from a dynamics perspective, not from an algebra perspective).*

5. line 130: The idea of connection gains having values above and below 0 is a new interesting constraint on the dynamics. This was partly discussed where the connection gain was treated as a gauge field in Cooray GK, Cooray V, Friston KJ. Cortical dynamics of neural-connectivity fields. Journal of Computational Neuroscience. 2025 Apr 10:1-9.

   *We read the referenced paper with interest, and to be honest, with some difficulty as we are not familiar with gauge field theory. As we read it, the word ”connection gains” (more specifically denoted as ’connectivity gain’ in the cited reference, with $W$ as a symbol) refers to what we denote ourselves as ’(synaptic) coupling strength’. To be sincere, we have not identified where in this reference the idea of connection gains having values above and below 0 has been discussed, even after careful reading. Hence, at this stage, we do not see where and how to make a reference to this paper in our text. We would welcome more specific guidance on that topic.*

# 3 Reviewer 2

## 3.1 General comments

Reviewer 2: The paper presents an interesting approach to include and study the effects of inhibitory connectivity in phenomenological models such as neural field/mass models. The authors use the framework of sensitivity analysis to see how perturbation in one area (control) affects the activation in another area (target), where the perturbed parameter is related to external input currents. The equations are modified to allow for both long-range excitatory and inhibitory connections (eq.4), which essentially includes modulating the fraction of long range excitatory connections and rest is projected to the inhibitory population. Overall, a mathematically thorough and well-written paper.

### 3.2 Major comments

While the idea is interesting and probably also has much need to be explored for development of modelling work in neuroscience, there are few major concerns that need to be addressed before I am convinced.

1. I am not very convinced with the idea of using just two-regions to demonstrate the concept. In such scenarios, the simplest case that should be tested is a three region model. May some examples of a structured sub-network involving three regions could be used (for ex. something from frontostriatal circuit ?)

   *We agree of course with the reviewer's idea to extend our framework beyond two-regions scenarios (as announced in the submitted version of Perspectives), and this is actually a work we already have done on our side for scenarios including a third area acting as a 'relay area' (keeping Control and Target area roles), such as conceived in many neural sub-networks (as well as any number of regions actually). As a matter of our answer to this comment however, we would prefer not to extend further the present paper, as it is self-consistent in the present state.*

   *Indeed, with our connectivity between two areas that can be either positive or negative on either direction, we have only four possible connectivity scenarios when considering the two-areas scenarios (the four that are exposed in the paper), namely 4 scenarios per couple of areas (here, one couple).*
   *If we now consider a system with three areas, i.e. three couples of areas, this number of possible connectivity scenarios amounts to* $4^3 = 64$ *scenarios to be considered. A full exploration of system behaviour with three areas hence deserves a full analysis considering all possible scenarios (e.g. to classify them in terms of response sensitivity of Target area to external forcing upon Control Area in presence of Relay area, the role of modulating some forcing upon Relay area, etc...).*

   *In support of our response, we added nonetheless an exposition of such a development for the system Control-Relay-Target in order to illustrate for readers how our nesting approach can be*

*extended to more regions, taking the 3-areas case as a test-bed. This extension is given as an additional SI 3, which is cited in some expansion of our Perspectives, lines 621-627.*

2. Relating the change in sign of the derivative to up or down-regulation is straightforward and trivial in a two region scenario. Again, the issue is that when it is scaled to three or more regions is this interpretation valid ? As the effect could pass through many (intermediate) areas ? This should be discussed and the validity beyond two-region scenario should be thoroughly discussed.

   *If we consider a system composed of two regions C-T and only take into account projections from Control to Target, the change in sign of the derivative in response to an upward or downward regulation of the external forcing exerted on Control would indeed be straightforward to guess, as the fixed points of the system could be causally linked. In such a case, it could even be fairly simple to derive formal expressions for sensitivities...*

   *With the connectivities we are examining in this article, this derivation of sensitivities seems less obvious, as they must incorporate the inter-area feedback coupling between the target and the control, as well as the intra-area feedback couplings within the two regions, all of which in turn influence how external forcing exerted on the Control drive the system toward fixed points, which are less obvious to express than in the causal case. Since these fixed-point values in turn determine the sensitivities, our work shows how to express not only their sign, but also their dependence on the parameter values throughout the system.*

   *As for the effect of more regions, see response above.*

3. The nested sensitivity approach taken by the authors assume separability (if I am not mistaken). What would be the author's view on using Jacobian matrix instead ? Probably, a comparison with Jacobian-based sensitivity would clarify the validity or limits of nested sensitivity?

   *Indeed. We have added an entire paragraph to address this point at the beginning of our new Perspectives section (lines 615-620), as well as another SI 2 to prove the formal equivalence between the two approaches "at the end of the day". See also the following paragraph on their differences (lines 621-627) in terms of legibility, physical interpretation, and practicality.*

4. Regarding the structure of the paper, it would help to have a brief into to sensitivity analysis and its applications to neural mass models as part of lit review (ex. see https://doi.org/10.1103/PhysRevE.110.044208) as well as other approaches to understand parameter importance (PLoS Comput. Biol. 14, e1006009 (2018), PLoS Comput. Biol. 15, e1006694 (2019))

   *We thank the reviewer for pointing us to these references. We have added some perspectives with regards to these approaches at the end of 1.1-Motivation, lines 42-52.*
