# Supplementary material for: Sensitivity analysis enlightens effects of connectivity in a Neural Mass Model under Control-Target mode
Source: PLoS Comput Biol. 2026 Mar 23;22(3):e1014035. doi: 10.1371/journal.pcbi.1014035 (PMC13008111; doi:10.1371/journal.pcbi.1014035)
Supplement: Attachment — Submitted filename: ValletEtAl-R2R_auresp_2.pdf [file pcbi.1014035.s007.pdf]

Response to Reviewers

Sensitivity analysis enlightens effects of connectivity in a Neural Mass Model under Control-Target mode

Vallet Anaïs[1], Blanco Stéphane[2], Chevallier Coline[2,3], Eustache Francis[1], Gautrais Jacques[2,3,*], Grandpeix Jean-Yves[4], Joly Jean-Louis[2], Segobin Shailendra[1], Gagnepain Pierre[1]

1 Normandie Univ, UNICAEN, PSL Research University, EPHE, INSERM, U1077, CHU de Caen, GIP Cyceron, Neuropsychologie et Imagerie de la Mémoire Humaine, 14000 Caen, France

2 LAPLACE, Université de Toulouse, CNRS, INPT, UPS, Toulouse, France

3 Centre de Recherches sur la Cognition Animale (CRCA), Centre de Biologie Intégrative (CBI), Université de Toulouse, CNRS, UPS, France

4 LMD/IPSL, Sorbonne Université, CNRS, École Polytechnique, ENS, Paris, France

* jacques.gautrais@cnrs.fr

To the editor and reviewers :

We thank you for your time and your favorable answers to our revised version.

As requested, the submission now includes all the codes needed to produce all numerical illustrations.

These codes are made available using Python 3, requesting only standard libraries (numpy, matplotlib, sys, os) and should run on any computer with Python installed.

Each figure can be computed using the corresponding ”run.py” script within the relevant sub-directory.

For convenience, we also add a bash script that allows readers to run all Python scripts at once from the terminal (approximately 2 to 3 hours).

Sincerely yours,

Jacques Gautrais
